# Negative psychological and physiological effects of social networking site use: The example of Facebook

**DOI:** 10.3389/fpsyg.2023.1141663

**Published:** 2023-08-03

**Authors:** Fabian J. Stangl, René Riedl, Roman Kiemeswenger, Christian Montag

**Affiliations:** ^1^Digital Business Institute, School of Business and Management, University of Applied Sciences Upper Austria, Steyr, Austria; ^2^Institute of Business Informatics – Information Engineering, Johannes Kepler University Linz, Linz, Austria; ^3^Department of Molecular Psychology, Institute of Psychology and Education, Ulm University, Ulm, Germany

**Keywords:** brain, Facebook, Neuro-Information-Systems, review, social networking sites, stress

## Abstract

Social networking sites (SNS), with Facebook as a prominent example, have become an integral part of our daily lives and more than four billion people worldwide use SNS. However, the (over-)use of SNS also poses both psychological and physiological risks. In the present article, we review the scientific literature on the risk of Facebook (over-)use. Addressing this topic is critical because evidence indicates the development of problematic Facebook use (“Facebook addiction”) due to excessive and uncontrolled use behavior with various psychological and physiological effects. We conducted a review to examine the scope, range, and nature of prior empirical research on the negative psychological and physiological effects of Facebook use. Our literature search process revealed a total of 232 papers showing that Facebook use is associated with eight major psychological effects (perceived anxiety, perceived depression, perceived loneliness, perceived eating disorders, perceived self-esteem, perceived life satisfaction, perceived insomnia, and perceived stress) and three physiological effects (physiological stress, human brain alteration, and affective experience state). The review also describes how Facebook use is associated with these effects and provides additional details on the reviewed literature, including research design, sample, age, and measures. Please note that the term “Facebook use” represents an umbrella term in the present work, and in the respective sections it will be made clear what kind of Facebook use is associated with a myriad of investigated psychological variables. Overall, findings indicate that certain kinds of Facebook use may come along with significant risks, both psychologically and physiologically. Based on our review, we also identify potential avenues for future research.

## 1. Introduction

Social networking sites (SNS) have become an integral part of our daily lives and play an important role in many areas. The main benefits of SNSs include creating connections between people (Hess et al., 2016), supporting collaboration and interpersonal communication (Kane et al., 2014), building social capital (Kwon et al., 2013) and generating marketing opportunities (Schreiner et al., 2021). Thus, SNSs provide a platform for social connection and sense of belonging (Zhao et al., 2012; Sariyska et al., 2019), which is considered a fundamental biological human need (Maslow, 1943; Kunc, 1992; Kenrick et al., 2010; Montag et al., 2020b; Rozgonjuk et al., 2021a). Also, SNSs promote continuous engagement due to their numerous features and functions. Examples include creating and maintaining personal profiles, sharing posts with family and friends, responding to notifications, or playing games (Frost and Rickwood, 2017; Chuang, 2020).

A prominent example of an SNS is Facebook. In fact, it is the most used SNS in the world, with around 2.96 billion active users each month (Statista, 2022d). American users, for example, spend an average of 33 min per day on Facebook (Statista, 2022a). An excessive and uncontrolled use of Facebook, however, also poses risks, both psychologically and physiologically. For example, frequent interaction with Facebook is associated with greater psychological distress (Chen and Lee, 2013). Mabe et al. (2014) found an association between regular social network use and perceived eating disorders. Other negative consequences that may result from excessive and uncontrolled Facebook use include the perception of depressive symptoms and anxiety (e.g., Wright et al., 2018), lower self-esteem (e.g., Hanna et al., 2017), as well as psychological (e.g., Brailovskaia et al., 2019a) and physiological stress (e.g., Campisi et al., 2017). Those who spend several hours a day on Facebook run the risk of losing control over their usage behavior (Brailovskaia and Margraf, 2017) and developing a Facebook addiction (Koc and Gulyagci, 2013). Please note that the addiction term is not officially recognized when discussing social media overuse (for debates, please see Carbonell and Panova, 2017) and it is of importance to not overpathologize everyday life behavior (Billieux et al., 2015).

Considering the potential risks of an excessive and uncontrolled Facebook use, the aim of this paper is to develop a concise and fundamental understanding of the negative psychological and physiological effects of Facebook use by synthesizing the accumulated knowledge of prior research. This review is therefore designed to provide an in-depth comprehension of the scope, range, and nature of the existing literature on the negative effects of Facebook use, including psychological and physiological effects (Hart, 1988). The term ‘Facebook use’ is an umbrella concept in our work. In the literature, different forms of Facebook use have been discussed ranging from overall use in terms of duration or frequency to active/passive use of Facebook (for recent updates, please see Verduyn et al., 2022) to addictive like use (Sindermann et al., 2020). Logically, different forms of Facebook use might be associated with different psychological effects. Therefore, each section will state in detail how Facebook use was operationalized in the different studies. When we speak in the following of “Facebook use,” it should be kept in mind that the term “Facebook use” here describes all kinds of Facebook use investigated in the literature. Accordingly, we address the following research question: **What negative psychological and physiological effects of Facebook use are identified by the current state of scientific research?**

The remainder of this paper is structured as follows. Section 2 describes the methodology of our review. Then, Section 3 follows with a presentation of the review results. We discuss our results in Section 4 by focusing on contributions and potentials for future research activities. Finally, in Section 5, we provide a concluding statement.

## 2. Review methodology

**Figure 1:**
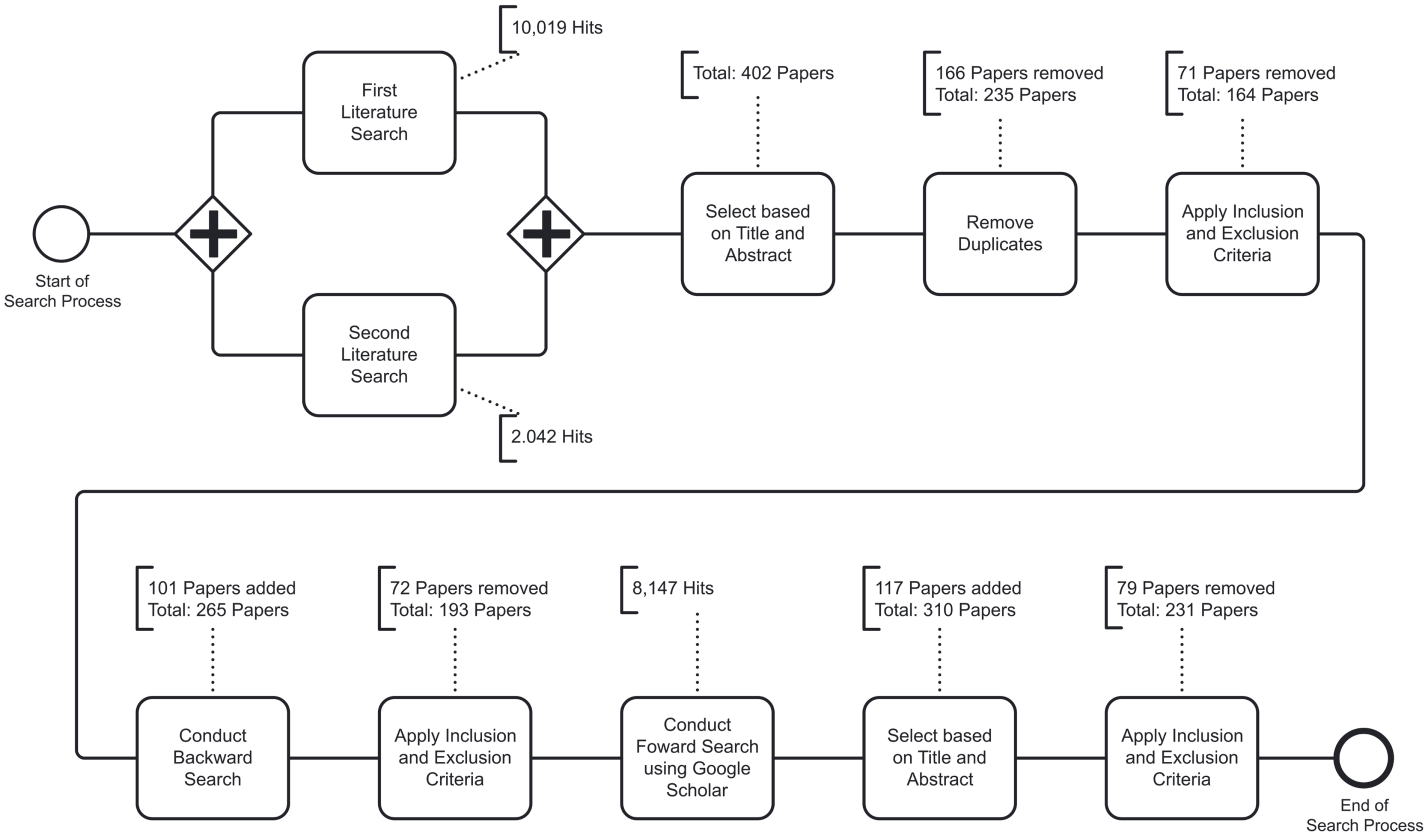
Overview of literature search process.

To examine the scope, range, and nature of prior research on the negative psychological and physiological effects of Facebook use, we conducted a scoping review to determine the extent of existing literature and the topics addressed therein (for an overview of the different literature review types, please see Paré et al., 2015; Schryen et al., 2017, 2020). The literature search process was based on existing methodological recommendations for conducting literature searches (Webster and Watson, 2002; Kitchenham and Charters, 2007; vom Brocke et al., 2009) and considered peer-reviewed journal and conference papers in English with no publication year restriction. As outlined in detail below, the present review includes literature published prior to and in April 2022. Based on primary selected papers after a two-wave literature search, we conducted an initial review, followed by backward search, a second review of the associated results, and a subsequent forward search. Figure 1 graphically summarizes the literature search process.

### 2.1. Search strategy

We conducted a two-wave literature search of five literature databases. We searched ACM Digital Library, IEEE Xplore, Science Direct, Scopus, and Web of Science using a combination of the term “Facebook” in conjunction with terms addressing the negative psychological and physiological effects of Facebook use. This search process yielded a total of 12,061 hits.

The following search term syntax was used to identify empirical studies that addressed the negative effects of Facebook use on a psychological and/or physiological level: (“Facebook”) AND (“psychological” OR “physiological” OR “depress*” OR “anxiety” OR “stress” OR “life satisfaction” OR “self-esteem” OR “loneliness” OR “consequence” OR “outcome” OR “disorder” OR “sleep*”). Note that the asterisk was used to generalize the term for searching when it can have multiple meanings (i.e., depress* includes “depression,” “depressing,” or “depressive” and other terms beginning with “depress”). In the databases IEEE Xplore, Science Direct, Scopus, and Web of Science the search terms could be used by default mode (that covers title, abstract, and keywords) to search for relevant papers. For the ACM database search, the abstract was used to narrow the search for relevant papers.

The first wave of our literature search was conducted in March 2022 and yielded 10,019 hits. The second wave was conducted in April 2022 with the goal of obtaining additional empirical studies on the negative physiological effects of Facebook use. To this end, we repeated our literature search in the mentioned literature databases and included the following physiological keywords [adopted from Riedl et al., 2020], resulting in the following search term syntax: (“Facebook”) AND (“Nervous system” OR “Neuro-Information Systems” OR “NeuroIS” OR “Neuroscience” OR “Brain” OR “Diffusion Tensor” OR “EEG” OR “fMRI” OR “Infared” OR “MEG” OR “Morpho*” OR “NIRS” OR “Positron emission” OR “Transcranial” OR “Dermal” OR “ECG” OR “ECG” OR “Electrocardiogram” OR “Electromyography” OR “Eye” OR “Facial” OR “Galvan*” OR “Heart” OR “HRV” OR “Muscular” OR “Oculo*” OR “Skin” OR “Blood” OR “Hormone” OR “Saliva” OR “Urine”). The second wave of our literature search yielded 2,042 hits. Note that NeuroIS is a scientific field which relies on neuroscience and neurophysiological knowledge and tools to better understand the development, use, and impact of information and communication technologies, including SNSs (Riedl et al., 2020).

In summary, search terms were chosen to reflect the topic of this paper in its entirety (e.g., “psychological” and “physiological”). Additionally, specific search terms were used to refer specifically to the psychological and physiological effects (e.g., “depress*” and “stress”). We also used keywords such as “ECG” that are representative of the data collection methods for measuring physiological effects to identify additional studies. In both waves of our literature search, we focused exclusively on peer-reviewed English-language journal and conference papers with no publication date restriction.

### 2.2. Filtering strategy

The filtering strategy included empirical studies that examined the negative effects of Facebook use on a psychological or physiological level as eligibility criteria. The psychological effects include those that are generally consistent with the 5th edition of the Diagnostic and Statistical Manual of Mental Disorders (DSM-5 Update) published by the American Psychiatric Association (2018). In addition, loneliness, life satisfaction, and self-esteem were also considered, although they are not included in the DSM-5 Update. They are considered as important psychological indicators and are critical for mental and physical well-being (Mann et al., 2004; Mushtaq et al., 2014) and subjective well-being along with life satisfaction (Pavot and Diener, 1993).

“Facebook use” was defined as use of all features of Facebook. Common conceptualizations of Facebook use include time spent on Facebook, number of Facebook friends, number of logins to Facebook, attitudes toward Facebook use, or indicators of an addiction construct consisting of a combination of behavioral and attitudinal variables (Frost and Rickwood, 2017): Therefore, we additionally considered the problematic facets of Facebook use, such as Facebook addiction (Turel et al., 2014) and Facebook intrusion (Cudo et al., 2019). Please note that in the literature Facebook overuse is often assessed via an addiction framework, but as mentioned above, neither Facebook addiction nor problematic Facebook use (the more neutral term) are officially recognized conditions in either DSM-5 (American Psychiatric Association, 2018) or the 11th revision of the International Classification of Diseases (ICD-11; World Health Organization, 2019). We do not want to go deeper into this discussion here but highlight that we aim to review both papers dealing with use *and* overuse of Facebook, independently of how the actual nature of overuse will be seen or characterized in a few years.

To be included in this review, we focused exclusively on peer-reviewed studies that empirically investigated negative effects of Facebook use on a psychological or physiological level. After conducting the two-wave literature search, we removed unrelated papers based on title and abstract, which left us with 402 papers. We then removed duplicates, which left us with 236 unique papers, which were then analyzed in-depth based on the full text. During this process, we also developed and applied the exclusion criteria listed in Table 1 to exclude papers that were not adequate in the light of the goal of this review. Following this filtering strategy, 165 unique papers remained for further analysis.

**Table 1:** Exclusion criteria for literature review.

Exclusion Criterion	Exemplary Source for Exclusion	Total Excluded
It was not possible to access the full text of the paper.	Ghali et al. (2022)	15
The paper was not in English.	Simon (2020)	5
The study examined social media use in general.	Dhir et al. (2019)	2
The study was not empirical.	Frost and Rickwood (2017)	11
The study used a qualitative research design.	Tran et al. (2015)	2
The study examined (non)authentic self-presentation.	Hall and Caton (2017)	2
The study result was not relevant to our review.	Nasr and Ben Rached (2021)	34

### 2.3. Backward and forward search

The 165 identified papers were then used for a backward search (i.e., searching the references), which yielded 101 additional papers, resulting in a total of 266 unique papers. After applying our exclusion criteria, 72 papers were removed, leaving a total of 194 papers. Next, we conducted a forward search (i.e., citation tracking) based on the 194 papers by using Google Scholar. This part of the search process resulted in 5,984 hits, of which 114 papers were selected for further investigation based on title and abstract, yielding a total of 308 papers. As part of this step, we excluded papers that were not peer-reviewed (e.g., Denti et al., 2012; Steggink, 2015). After applying our full list of exclusion criteria, 76 papers were removed, leaving a total of 232 papers which constitute the basis of all analyses in the present review.

Overall, this review includes empirical literature on the negative psychological and physiological effects of Facebook use published before and in April 2022. Specifically, 217 papers deal with the negative *psychological* effects of Facebook use, consisting of 213 journal papers (98%) and 4 conference papers (2%), and the remaining 15 papers (all journal articles) deal with the negative *physiological* effects of Facebook use. The Supplementary material contains an overview of the N = 232 papers.

## 3. Review results

**Figure 2:**
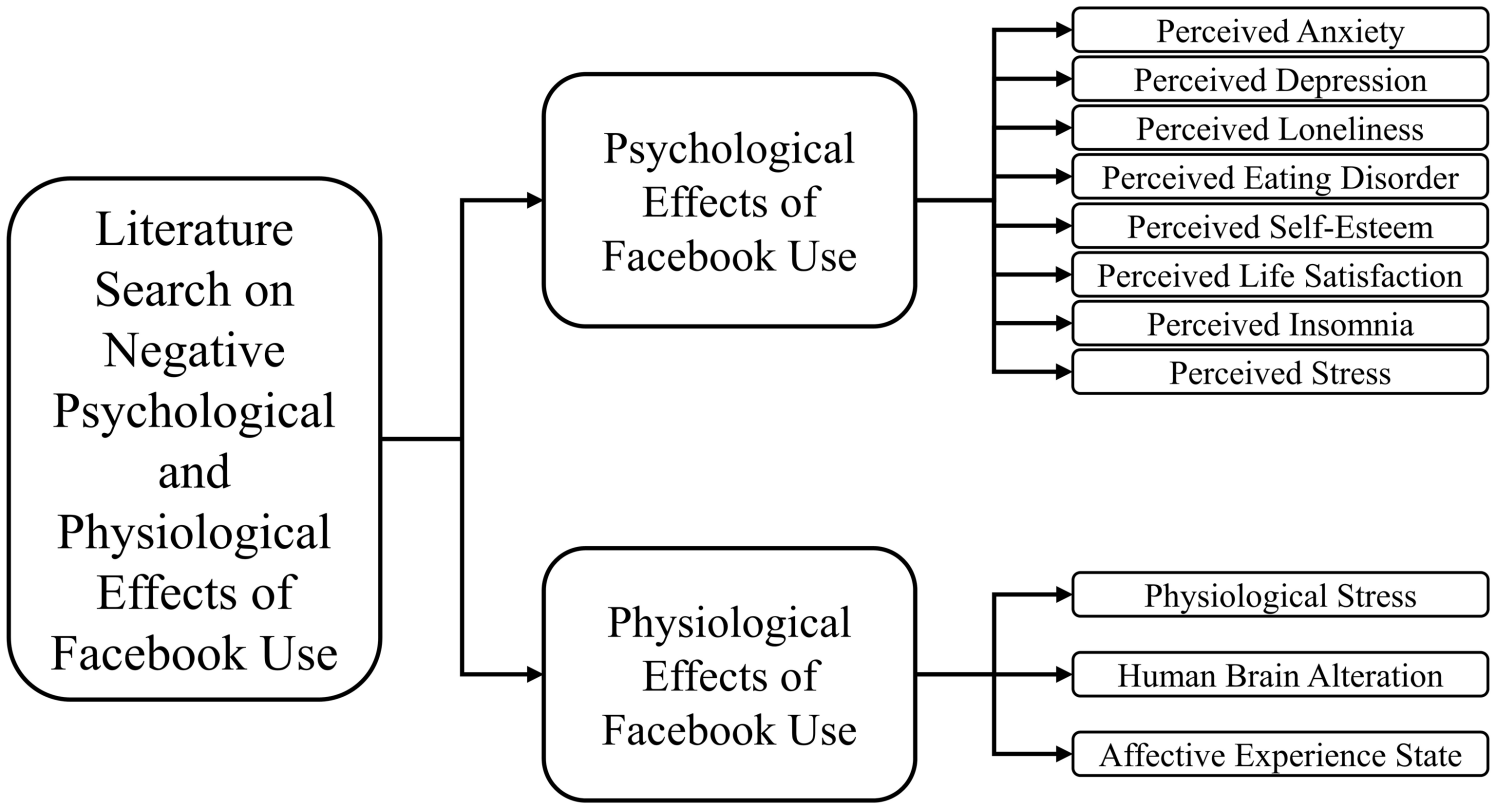
Overview of main findings of literature search process.

In this section, we present the main findings of our review. Our literature search process revealed a total of 232 papers showing that Facebook use is associated with eight psychological effects (perceived anxiety, perceived depression, perceived loneliness, perceived eating disorders, perceived self-esteem, perceived life satisfaction, perceived insomnia, and perceived stress) and three physiological effects (physiological stress, human brain alteration, and affective experience state). Figure 2 graphically summarizes the main findings of our literature search process. The psychological effects of Facebook use are described in detail below, followed by the physiological effects. The Supplementary material provides additional details on the identified studies by construct (i.e., identified psychological and physiological effects), including research design, sample, age, measures, and strength of associations between Facebook use and its effects.

### 3.1. Psychological effects of Facebook Use

We found 217 empirical studies that examined psychological effects of Facebook use. The 217 studies included 183 cross-sectional studies (85%), 24 longitudinal studies (11%), 5 experimental studies (2%), and 5 studies that conducted a multimethod research design (2%). Our analysis revealed that Facebook use is associated with eight major psychological effects, which we discuss in the following. We summarize the identified papers on the psychological effects of Facebook use with their effect type, based on results which are reported as statistically significant (negative [−], positive [+], no effect [∼] in Table 2). To reveal the scope, range, and nature of prior empirical research on how Facebook use is associated with these psychological effects, we considered the research context of the identified studies rather than just the effect direction. For example, we classified the Błachnio et al.’s (2021) paper as a study reporting a negative effect because it found that Facebook intrusion was positively associated with perceived anxiety. Note that we also classified a few papers as “descriptive [/],” referring to studies that reported only descriptive statistics such as frequency distributions associated with Facebook addiction without correlative or more sophisticated statistics (Jha et al., 2016; Norman et al., 2017).

**Table 2:** Studies on psychological effects of Facebook use.

Construct	Details		
Perceived Anxiety	Studies	Atroszko et al. (2018) [−]; Atroszko et al. (2022) [−]; Błachnio et al. (2021) [−]; Brailovskaia and Margraf (2016) [∼]; Brailovskaia and Margraf (2017) [−][∼]; Brailovskaia et al. (2020a) [−]; Chabrol et al. (2017) [−]; Clayton et al. (2013) [−]; Cury et al. (2022) [/]; da Veiga et al. (2019) [−]; Davidson and Farquhar (2014) [∼]; Dempsey et al. (2019) [−][+]; Eşkisu et al. (2020) [−]; Farahani et al. (2011) [−]; Flynn et al. (2018) [−]; Foroughi et al. (2019) [−]; Grieve et al. (2013) [+]; Hanna et al. (2017) [−][∼]; Hanprathet et al. (2015) [/]; Ho et al. (2021a) [−]; Hu et al. (2017) [∼]; Hussain et al. (2019) [∼]; Khalil et al. (2022) [∼]; Kim et al. (2020) [/]; Koc and Gulyagci (2013) [−][∼]; Labrague (2014) [−][∼]; Lee (2014) [−]; Lee-Won et al. (2015) [−][∼]; Louragli et al. (2019) [−]; Marder et al. (2016) [∼]; McCord et al. (2014) [−][∼]; Nasser et al. (2019) [−]; Nazzal et al. (2021) [−]; Ögel-Balaban and Altan (2020) [−][∼]; Pal et al. (2018) [−]; Rae and Lonborg (2015) [∼]; Shaw et al. (2015) [−][∼]; Sillence et al. (2021) [∼]; Soraci et al. (2020) [∼]; Sotero et al. (2019) [−]; Sternberg et al. (2020) [−]; Sternberg et al. (2018) [−][∼]; Vannucci et al. (2019) [∼]; Verseillié et al. (2021) [−]; Wright et al. (2018) [−]; Xie and Karan (2019) [−][∼]; Zaffar et al. (2015) [−]	
	Descriptive Information	Total number of studies	47
		Number of studies reporting a negative effect	33
		Number of studies reporting a positive effect	2
		Number of studies reporting no effect	20
		Number of descriptive studies	3
	Negative Effects	Connection as motive for using Facebook (Clayton et al., 2013), entertainment as motive for using Facebook (Ögel-Balaban and Altan, 2020), Facebook addiction (Koc and Gulyagci, 2013; Zaffar et al., 2015; Brailovskaia and Margraf, 2017; Atroszko et al., 2018, 2022; da Veiga et al., 2019; Foroughi et al., 2019; Louragli et al., 2019; Sotero et al., 2019; Xie and Karan, 2019; Eşkisu et al., 2020; Brailovskaia et al., 2020a; Verseillié et al., 2021), Facebook intensity (Pal et al., 2018; Xie and Karan, 2019; Nazzal et al., 2021), Facebook intrusion (Błachnio et al., 2021), frequency of Facebook use (Sternberg et al., 2020), general Facebook use (Farahani et al., 2011), liking behavior on Facebook (Wright et al., 2018), lying behavior on Facebook (Wright et al., 2018), number of Facebook friends (Flynn et al., 2018; Nazzal et al., 2021), passive Facebook use (Shaw et al., 2015; Hanna et al., 2017), perceived emotional connectedness to Facebook (Clayton et al., 2013), perceived emotional engagement with Facebook (Verseillié et al., 2021), perceived frequency of having a negative feeling from social comparison on Facebook (Lee, 2014), perceived frequency of social comparison on Facebook (Lee, 2014), perceived social comparison on Facebook (Flynn et al., 2018), problematic Facebook use (Lee-Won et al., 2015; Chabrol et al., 2017; Dempsey et al., 2019; Nasser et al., 2019; Ho et al., 2021a), risky and impulsive Facebook use (Flynn et al., 2018), time spent on Facebook (Labrague, 2014; Shaw et al., 2015; Flynn et al., 2018; Sternberg et al., 2018; Nazzal et al., 2021), use of Facebook for broadcasting (Xie and Karan, 2019), use of Facebook for interactive communication (Shaw et al., 2015), and use of socially interactive features of Facebook (McCord et al., 2014)	
	Positive Effects	Frequency of Facebook use (Dempsey et al., 2019) and perceived social connectedness from the use of Facebook (Grieve et al., 2013)	
	No Effects	Academic motive for using Facebook (Koc and Gulyagci, 2013), active Facebook use (Hanna et al., 2017), connection as motive for using Facebook (Rae and Lonborg, 2015), daily informational motive for using Facebook (Koc and Gulyagci, 2013), Facebook account length (Hussain et al., 2019; Ögel-Balaban and Altan, 2020), Facebook addiction (Brailovskaia and Margraf, 2017; Hussain et al., 2019; Soraci et al., 2020; Khalil et al., 2022), Facebook intensity (Davidson and Farquhar, 2014; Labrague, 2014; Marder et al., 2016; Hu et al., 2017), Facebook session length (Hussain et al., 2019), frequency of Facebook use (Lee-Won et al., 2015; Ögel-Balaban and Altan, 2020), friendship as motive for using Facebook (Rae and Lonborg, 2015), general Facebook use (Brailovskaia and Margraf, 2016; Vannucci et al., 2019), information as motive for using Facebook (Rae and Lonborg, 2015), inspection time of Facebook updates (Hussain et al., 2019), inspection time of social updates on Facebook (Hussain et al., 2019), number of activities during Facebook use (Sternberg et al., 2018), number of Facebook friends (Labrague, 2014; Ögel-Balaban and Altan, 2020), passive Facebook use (Shaw et al., 2015), perceived content production on Facebook (Shaw et al., 2015), perceived extent of communication with Facebook friends (Ögel-Balaban and Altan, 2020), perceived frequency of posting on Facebook (Ögel-Balaban and Altan, 2020), social motive for using Facebook (Koc and Gulyagci, 2013), time spent on Facebook (Shaw et al., 2015; Hanna et al., 2017), use of Facebook for bonding social capital (Ögel-Balaban and Altan, 2020), use of Facebook for bridging social capital (Ögel-Balaban and Altan, 2020), use of Facebook for directed communication (Xie and Karan, 2019), use of Facebook for interactive communication (Shaw et al., 2015), use of socially interactive features of Facebook (McCord et al., 2014; Sillence et al., 2021), and weekly time commitment on Facebook (Koc and Gulyagci, 2013)	
	Example	Facebook intrusion has been linked to the negative psychological effects of Facebook use associated with perceived anxiety (Błachnio et al., 2021).	
Perceived Depression	Studies	Ahamed et al. (2021) [−]; Alfasi (2019) [−]; Atroszko et al. (2022) [−]; Bais and Reyes (2020) [−]; Bendayan and Blanca Mena (2019) [−]; Brailovskaia and Margraf (2016) [−]; Brailovskaia and Margraf (2017) [−][∼]; Brailovskaia and Margraf (2019) [+][∼]; Brailovskaia et al. (2019a) [−]; Brailovskaia et al. (2019c) [−][∼]; Brailovskaia et al. (2019b) [−]; Brailovskaia et al. (2019d) [−]; Çakıcı et al. (2020) [∼]; Chabrol et al. (2017) [−]; Chow and Wan (2017) [−][∼]; Cudo et al. (2020a) [−][∼]; Cury et al. (2022) [/]; da Veiga et al. (2019) [−]; Damota (2019) [−]; Datu et al. (2012) [∼]; Dempsey et al. (2019) [−][∼]; Dibb and Foster (2021) [−][∼]; Eşkisu et al. (2020) [−]; Farahani et al. (2011) [∼]; Faranda and Roberts (2019) [−][∼]; Flynn et al. (2018) [−][∼]; Foroughi et al. (2019) [−]; Frison and Eggermont (2015) [−][+][∼]; Frison and Eggermont (2016a) [−][+][∼]; Frison and Eggermont (2020) [−]; Frison et al. (2019) [−][+][∼]; Giota and Kleftaras (2013) [−]; Grieve et al. (2013) [+]; große Deters and Mehl (2013) [∼]; Hanna et al. (2017) [∼]; Hanprathet et al. (2015) [/]; Ho (2021a) [−]; Ho et al. (2021a) [−]; Hong et al. (2014) [−][∼]; Hussain et al. (2019) [−][∼]; Iovu et al. (2020) [−]; Jeri-Yabar et al. (2019) [∼]; Kang et al. (2013) [−][+][∼]; Khalil et al. (2022) [∼]; Khattak et al. (2017) [−]; Kim et al. (2020) [/]; Koc and Gulyagci (2013) [−][∼]; Kulkarni and Deshpande (2019) [−]; Labrague (2014) [−][∼]; Lee (2014) [−]; Locatelli et al. (2012) [∼]; Maglunog and Dy (2019) [∼]; McCloskey et al. (2015) [−][∼]; Michikyan et al. (2015) [−][∼]; Nasser et al. (2019) [−]; Nazzal et al. (2021) [−]; Nisar et al. (2019) [−][+][∼]; Norman et al. (2017) [/]; Ozimek and Bierhoff (2020) [−][∼]; Pal et al. (2018) [∼]; Park et al. (2013) [−][+][∼]; Przepiórka and Błachnio (2020) [−]; Puccio et al. (2016) [−]; Rachubińska et al. (2021) [−]; Rae and Lonborg (2015) [+]; Rosen et al. (2013b) [−][+]; Rosenthal et al. (2016) [+]; Scherr and Brunet (2017) [−][+][∼]; Scherr et al. (2019) [−][∼]; Shaw et al. (2015) [−][∼]; Simoncic et al. (2014) [∼]; Soraci et al. (2020) [∼]; Sotero et al. (2019) [−]; Steers et al. (2014) [−][∼]; Sternberg et al. (2018) [−][∼]; Tandoc Jr. and Goh (2023) [−][∼]; Tandoc Jr. et al. (2015) [∼]; Teo et al. (2019) [−]; Tosun and Kaşdarma (2020) [−][∼]; Türkmen et al. (2022) [∼]; Vannucci et al. (2019) [−]; Verseillié et al. (2021) [−]; Walburg et al. (2016) [−]; Walker et al. (2015) [−][∼]; Wright et al. (2018) [−][∼]; Wright et al. (2013) [−][∼]; Yeshua-Katz and Zilberstein (2021) [−]; Zaffar et al. (2015) [−]; Zhang (2017) [+][∼]	
	Descriptive Information	Total number of studies	89
		Number of studies reporting a negative effect	66
		Number of studies reporting a positive effect	13
		Number of studies reporting no effect	47
		Number of descriptive studies	4
	Negative Effects	Active private Facebook use (Frison and Eggermont, 2020), active public Facebook use (Frison and Eggermont, 2016a, 2020), browsing own Facebook newsfeed (Alfasi, 2019), compare/impress as motive for false self-presentation on Facebook (Michikyan et al., 2015), daily Facebook use (Brailovskaia et al., 2019b), deception as motive for false self-presentation on Facebook (Michikyan et al., 2015), Facebook account length (Hussain et al., 2019), Facebook addiction (Koc and Gulyagci, 2013; Hong et al., 2014; Zaffar et al., 2015; Brailovskaia and Margraf, 2017; Khattak et al., 2017; da Veiga et al., 2019; Damota, 2019; Foroughi et al., 2019; Kulkarni and Deshpande, 2019; Sotero et al., 2019; Brailovskaia et al., 2019b,d; Bais and Reyes, 2020; Eşkisu et al., 2020; Iovu et al., 2020; Rachubińska et al., 2021; Verseillié et al., 2021; Ho, 2021a; Atroszko et al., 2022), Facebook intensity (Iovu et al., 2020; Ahamed et al., 2021; Nazzal et al., 2021), Facebook intrusion (Bendayan and Blanca Mena, 2019; Przepiórka and Błachnio, 2020; Cudo et al., 2020a), Facebook surveillance (Scherr et al., 2019), frequency of Facebook use (Kang et al., 2013; Brailovskaia et al., 2019b), general Facebook use (Rosen et al., 2013a; Brailovskaia and Margraf, 2016; Vannucci et al., 2019; Brailovskaia et al., 2019a; Tandoc Jr. and Goh, 2023), ideal self-presentation on Facebook (Michikyan et al., 2015), impression management as motive for using Facebook (Rosen et al., 2013a), inspection time of social updates on Facebook (Hussain et al., 2019), interpersonal motives for using Facebook (Wright et al., 2013), liking behavior on Facebook (Wright et al., 2018), more frequent in-person social interaction on Facebook (Teo et al., 2019), number of accumulated points in Facebook (Park et al., 2013), number of accumulated tips in Facebook (Park et al., 2013), number of Facebook friends (Rosen et al., 2013a; Nazzal et al., 2021), passive Facebook use (Frison and Eggermont, 2016a, 2020; Dibb and Foster, 2021), perceived attraction to online social support on Facebook (Giota and Kleftaras, 2013), perceived content production on Facebook (Shaw et al., 2015), perceived downward social comparison on Facebook (Steers et al., 2014), perceived downward-identification in social comparison on Facebook (Kang et al., 2013), perceived emotional engagement with Facebook (Verseillié et al., 2021), perceived emotional support on Facebook (McCloskey et al., 2015), perceived frequency of having a negative feeling from social comparison on Facebook (Lee, 2014), perceived frequency of social comparison on Facebook (Lee, 2014), perceived level of activity on Facebook (Michikyan et al., 2015), perceived level of watching on Facebook (Ozimek and Bierhoff, 2020), perceived negative social support on Facebook (McCloskey et al., 2015), perceived non-directional social comparison on Facebook (Steers et al., 2014), perceived non-directional social comparison on Facebook by male (Steers et al., 2014), perceived online physical appearance comparison (Walker et al., 2015), perceived social comparison direction on Facebook (Faranda and Roberts, 2019), perceived social comparison on Facebook (Puccio et al., 2016; Chow and Wan, 2017; Flynn et al., 2018; Alfasi, 2019), perceived social comparison when using Facebook passively (Nisar et al., 2019), perceived social support seeking through Facebook (Frison and Eggermont, 2015), perceived tendency to socially compare on Facebook (Dibb and Foster, 2021), perceived upward social comparison on Facebook (Steers et al., 2014; Tosun and Kaşdarma, 2020; Dibb and Foster, 2021), perceived upward-contrast in social comparison on Facebook (Kang et al., 2013), private Facebook interaction (Frison et al., 2019), problematic Facebook use (Walburg et al., 2016; Chabrol et al., 2017; Dempsey et al., 2019; Nasser et al., 2019; Ho et al., 2021a), reduction in time spent on Facebook (Brailovskaia et al., 2020b), relationship formation as motive for using Facebook (Scherr and Brunet, 2017), risky and impulsive Facebook use (Flynn et al., 2018), social integrative motives for using Facebook (Wright et al., 2013), time spent on Facebook (Kang et al., 2013; Labrague, 2014; Steers et al., 2014; Chow and Wan, 2017; Scherr and Brunet, 2017; Flynn et al., 2018; Sternberg et al., 2018; Frison et al., 2019; Frison and Eggermont, 2020; Nazzal et al., 2021; Yeshua-Katz and Zilberstein, 2021), time spent on Facebook by females (Steers et al., 2014), time spent on Facebook by males (Steers et al., 2014), use of Facebook for interactive communication (Shaw et al., 2015), and weekly time commitment on Facebook (Wright et al., 2013)	
	Positive Effects	Bullying or meanness as type of perceived negative Facebook experience (Rosenthal et al., 2016), misunderstandings as type of perceived negative Facebook experience (Rosenthal et al., 2016), number of Facebook friends (Rosen et al., 2013a; Rae and Lonborg, 2015; Brailovskaia and Margraf, 2019), number of physical locations which a user has tagged on Facebook (Park et al., 2013), perceived negative Facebook experience (Rosenthal et al., 2016), perceived social comparison when using Facebook actively (Nisar et al., 2019), perceived social connectedness from the use of Facebook (Grieve et al., 2013), perceived social support on Facebook (Zhang, 2017), perceived social support through Facebook (Frison and Eggermont, 2015, 2016a; Frison et al., 2019), perceived upward-identification in social comparison on Facebook (Kang et al., 2013), relationship maintenance as motive for using Facebook (Scherr and Brunet, 2017), and unwanted contact as type of perceived negative Facebook experience (Rosenthal et al., 2016)	
	No Effects	Academic motive for using Facebook (Koc and Gulyagci, 2013), active Facebook use (Simoncic et al., 2014; Hanna et al., 2017; Dibb and Foster, 2021), active posting on Facebook (große Deters and Mehl, 2013), active private Facebook use (Frison and Eggermont, 2016a), commenting as motive for using Facebook (Maglunog and Dy, 2019), creating or RSVPing to events as motive for using Facebook (Maglunog and Dy, 2019), daily Facebook use (Simoncic et al., 2014; Brailovskaia et al., 2019b), daily informational motive for using Facebook (Koc and Gulyagci, 2013), entertainment/distraction as motive for using Facebook (Scherr and Brunet, 2017), exploration as motive for false self-presentation on Facebook (Michikyan et al., 2015), Facebook account length (Locatelli et al., 2012; Kang et al., 2013), Facebook addiction (Brailovskaia and Margraf, 2017; Hussain et al., 2019; Brailovskaia et al., 2019b; Çakıcı et al., 2020; Soraci et al., 2020; Khalil et al., 2022), Facebook intensity (Labrague, 2014; Walker et al., 2015; Pal et al., 2018), Facebook network size (Zhang, 2017), Facebook session length (Hussain et al., 2019), Facebook surveillance (Scherr et al., 2019), frequency of Facebook use (Kang et al., 2013; Tandoc Jr. et al., 2015; Dempsey et al., 2019; Maglunog and Dy, 2019; Cudo et al., 2020a; Türkmen et al., 2022), general Facebook use (Farahani et al., 2011; Datu et al., 2012; Faranda and Roberts, 2019; Jeri-Yabar et al., 2019; Tandoc Jr. and Goh, 2023), inspection time of Facebook updates (Hussain et al., 2019), level of interest in Facebook use (Kang et al., 2013), lying behavior on Facebook (Wright et al., 2018), number of activities during Facebook use (Sternberg et al., 2018), number of Facebook friends (Chow and Wan, 2017; Flynn et al., 2018; Labrague, 2014; Locatelli et al., 2012; Park et al., 2013; Tandoc Jr. et al., 2015; Wright et al., 2013), number of Facebook logins (Steers et al., 2014), number of Facebook pages a user has marked as like (Park et al., 2013), number of groups on Facebook for which a user is an administrator (Park et al., 2013), number of groups on Facebook to which a user belongs (including groups of which a user is an administrator) (Park et al., 2013), number of interest items listed on the user’s Facebook profile (Park et al., 2013), number of pending incoming friend requests on Facebook (Park et al., 2013), passive Facebook use (Shaw et al., 2015; Frison and Eggermont, 2016a; Hanna et al., 2017; Tosun and Kaşdarma, 2020), perceived downward social comparison on Facebook (Dibb and Foster, 2021), perceived downward-contrast in social comparison on Facebook (Kang et al., 2013), perceived enacted social support on Facebook (Zhang, 2017), perceived frequency of commenting status updates on Facebook (Brailovskaia and Margraf, 2019), perceived frequency of writing in discussion groups on Facebook (Brailovskaia and Margraf, 2019), perceived frequency of writing negative status updates on Facebook (Locatelli et al., 2012), perceived frequency of writing online messages on Facebook (Brailovskaia and Margraf, 2019), perceived frequency of writing positive status updates on Facebook (Locatelli et al., 2012), perceived frequency of writing status updates on Facebook (Locatelli et al., 2012; Brailovskaia and Margraf, 2019), perceived instrumental social support on Facebook (McCloskey et al., 2015), perceived level of acting on Facebook (Ozimek and Bierhoff, 2020), perceived level of activity on Facebook (Ozimek and Bierhoff, 2020), perceived level of impressing on Facebook (Ozimek and Bierhoff, 2020), perceived non-directional social comparison on Facebook by female (Steers et al., 2014), perceived social comparison direction on Facebook (Faranda and Roberts, 2019), perceived social comparison on Facebook (Chow and Wan, 2017; Nisar et al., 2019), perceived social comparison orientation on Facebook (Faranda and Roberts, 2019), perceived social support on Facebook (McCloskey et al., 2015), perceived social support through Facebook (Frison and Eggermont, 2015, 2016a; Frison et al., 2019), perceived upward social comparison on Facebook (Tosun and Kaşdarma, 2020), perceived upward-identification in social comparison on Facebook (Kang et al., 2013), playing games as motive for using Facebook (Maglunog and Dy, 2019), posting photos as motive for using Facebook (Maglunog and Dy, 2019), posting status updates as motive for using Facebook (Maglunog and Dy, 2019), posting videos as motive for using Facebook (Maglunog and Dy, 2019), private Facebook interaction (Frison et al., 2019), real self-presentation on Facebook (Michikyan et al., 2015), sending private messages as motive for using Facebook (Maglunog and Dy, 2019), sharing links as motive for using Facebook (Maglunog and Dy, 2019), social motive for using Facebook (Koc and Gulyagci, 2013), tagging photos as motive for using Facebook (Maglunog and Dy, 2019), tagging videos as motive for using Facebook (Maglunog and Dy, 2019), time spent on Facebook (Locatelli et al., 2012; Steers et al., 2014; Michikyan et al., 2015; Shaw et al., 2015; Tandoc Jr. et al., 2015; Hanna et al., 2017; Zhang, 2017; Frison et al., 2019; Maglunog and Dy, 2019; Nisar et al., 2019), time spent on Facebook apps (including games) (Hong et al., 2014), time spent on Facebook chat rooms (Hong et al., 2014), time spent on Facebook newsfeeds (Hong et al., 2014), viewing other Facebook profiles as motive for using Facebook (Maglunog and Dy, 2019), viewing videos as motive for using Facebook (Maglunog and Dy, 2019), and weekly time commitment on Facebook (Koc and Gulyagci, 2013)	
	Example	Facebook intrusion has been linked to the negative psychological effects of Facebook use associated with perceived depression (Bendayan and Blanca Mena, 2019; Przepiórka and Błachnio, 2020; Cudo et al., 2020a).	
Perceived Loneliness	Studies	Ahmed (2018) [−][∼]; Atroszko et al. (2018) [−]; Aung and Tin (2020) [−]; Aydın et al. (2013) [∼]; Baker and Oswald (2010) [∼]; Biolcati et al. (2018) [−]; Błachnio and Przepiórka (2019) [−]; Błachnio et al. (2018) [−][∼]; Błachnio et al. (2016b) [−][∼]; Brown et al. (2021) [+][∼]; Chavez and Chavez Jr. (2017) [−]; Clayton et al. (2013) [−][∼]; Dibb and Foster (2021) [−][∼]; Francis (2022) [+][∼]; Frison and Eggermont (2020) [−][∼]; Goljović (2017) [−]; große Deters and Mehl (2013) [+]; Ho (2021a) [−]; Ho et al. (2021a) [−]; Ho et al. (2021b) [−]; Jin (2013) [−][+][∼]; Karakose et al. (2016) [∼]; Kross et al. (2013) [−]; Kumar et al. (2019) [−]; Lemieux et al. (2013) [−]; Lim and Yang (2019) [−]; Lou et al. (2012) [+][∼]; Omar and Subramanian (2013) [−]; Phu and Gow (2019) [−][+][∼]; Primi et al. (2021) [−]; Rachubińska et al. (2021) [+]; Rahman and Zakaria (2021) [−]; Rajesh and Rangaiah (2020) [−][∼]; Ryan and Xenos (2011) [−][+][∼]; Salem et al. (2016) [−]; Satici (2019) [−][∼]; Shettar et al. (2017) [−]; Skues et al. (2012) [−][+][∼]; Smith and Short (2022) [−]; Stieger (2019) [∼]; Teppers et al. (2014) [−][∼]; Türkmen et al. (2022) [∼]; Uram and Skalski (2022) [∼]; Wang et al. (2018) [−][∼]; Ye et al. (2021) [∼]; Zaffar et al. (2015) [∼]	
	Descriptive Information	Total number of studies	46
		Number of studies reporting a negative effect	33
		Number of studies reporting a positive effect	9
		Number of studies reporting no effect	25
		Number of descriptive studies	0
	Negative Effects	Active public Facebook use (Frison and Eggermont, 2020; Wang et al., 2018), browsing own Facebook newsfeed (Ahmed, 2018), compensatory Facebook use (Goljović, 2017), connection as motive for using Facebook (Clayton et al., 2013; Jin, 2013), decrease loneliness as motive for using Facebook (Teppers et al., 2014), entertainment as motive for using Facebook (Teppers et al., 2014; Błachnio et al., 2016b), Facebook addiction (Omar and Subramanian, 2013; Saleem et al., 2016; Błachnio et al., 2016b; Chavez and Chavez Jr., 2017; Goljović, 2017; Shettar et al., 2017; Atroszko et al., 2018; Biolcati et al., 2018; Satici, 2019; Aung and Tin, 2020; Rajesh and Rangaiah, 2020; Ho, 2021a; Ho et al., 2021b; Smith and Short, 2022), Facebook intrusion (Błachnio et al., 2018; Błachnio and Przepiórka, 2019), general Facebook use (Kross et al., 2013), maintaining relationships as motive for using Facebook (Teppers et al., 2014), passive engagement on Facebook (Ryan and Xenos, 2011), passive Facebook use (Frison and Eggermont, 2020; Dibb and Foster, 2021), perceived persistence of use or overuse of Facebook (Phu and Gow, 2019), perceived positive attitude towards Facebook (Teppers et al., 2014), perceived tendency to socially compare on Facebook (Dibb and Foster, 2021), perceived upward social comparison on Facebook (Lim and Yang, 2019; Dibb and Foster, 2021), personal contact as motive for using Facebook (Teppers et al., 2014), problematic Facebook use (Primi et al., 2021; Ho et al., 2021a), social inclusion as motive for using Facebook (Teppers et al., 2014), social skills compensation as motive for using Facebook (Teppers et al., 2014), time spent on Facebook (Frison and Eggermont, 2020; Kumar et al., 2019; Lemieux et al., 2013; Rahman and Zakaria, 2021; Skues et al., 2012; Teppers et al., 2014; Wang et al., 2018), use of Facebook for news and information (Ryan and Xenos, 2011), and use of Facebook for real-time social interaction (Ryan and Xenos, 2011)	
	Positive Effects	Active posting on Facebook (große Deters and Mehl, 2013), active social contributions on Facebook (Ryan and Xenos, 2011), active use of Facebook (Jin, 2013), Facebook addiction (Rachubińska et al., 2021), Facebook intensity (Lou et al., 2012), Facebook network size (Brown et al., 2021), initiating of communication on Facebook (Jin, 2013), number of activities during Facebook use (Francis, 2022), number of Facebook friends (Skues et al., 2012; Jin, 2013; Phu and Gow, 2019), perceived persistence of use or overuse of Facebook (Phu and Gow, 2019), perceived satisfaction of Facebook use (Jin, 2013), and use of Facebook for news and information (Ryan and Xenos, 2011)	
	No Effects	Active Facebook use (Dibb and Foster, 2021), active private Facebook use (Frison and Eggermont, 2020), active public Facebook use (Wang et al., 2018), active social contributions on Facebook (Ryan and Xenos, 2011), active use of Facebook (Jin, 2013), communication as motive for using Facebook (Aydın et al., 2013), contacting old friends as motive for using Facebook (Aydın et al., 2013), decrease loneliness as motive for using Facebook (Teppers et al., 2014), entertainment as motive for using Facebook (Teppers et al., 2014), Facebook access time via PC (Ye et al., 2021), Facebook access time via smartphone (Ye et al., 2021), Facebook addiction (Zaffar et al., 2015; Karakose et al., 2016; Satici, 2019; Uram and Skalski, 2022), Facebook intensity (Phu and Gow, 2019; Rajesh and Rangaiah, 2020; Francis, 2022), Facebook intrusion (Błachnio et al., 2018), Facebook network cluster (Brown et al., 2021), Facebook network density (Brown et al., 2021), Facebook network path length (Brown et al., 2021), following photos, videos, status, comments as motive for using Facebook (Aydın et al., 2013), frequency of Facebook use (Türkmen et al., 2022), general Facebook use (Baker and Oswald, 2010; Błachnio et al., 2016b), maintaining relationships as motive for using Facebook (Teppers et al., 2014), motive for using Facebook (Lou et al., 2012), new acquaintance as motive for using Facebook (Aydın et al., 2013), number of Facebook friends (Stieger, 2019), number of Facebook logins (Skues et al., 2012), passive engagement on Facebook (Ryan and Xenos, 2011), perceived boredom of use of Facebook (Phu and Gow, 2019), perceived downward social comparison on Facebook (Dibb and Foster, 2021), perceived emotional connectedness to Facebook (Clayton et al., 2013), perceived frequency of posting on Facebook (Ye et al., 2021), perceived overuse of Facebook (Phu and Gow, 2019), perceived positive attitude towards Facebook (Teppers et al., 2014), perceived self-expression on Facebook (Phu and Gow, 2019), perceived use experience of Facebook (Jin, 2013), personal contact as motive for using Facebook (Teppers et al., 2014), playing games on Facebook as motive for using Facebook (Aydın et al., 2013), sharing photos, videos, and notifications on Facebook as motive for using Facebook (Aydın et al., 2013), social inclusion as motive for using Facebook (Teppers et al., 2014), social skills compensation as motive for using Facebook (Teppers et al., 2014), time spent on Facebook (Jin, 2013; Phu and Gow, 2019; Teppers et al., 2014; Wang et al., 2018), time spent on Facebook for private purposes (Stieger, 2019), use of Facebook chat (Ahmed, 2018), use of Facebook for news and information (Ryan and Xenos, 2011), and use of Facebook for real-time social interaction (Ryan and Xenos, 2011)	
	Example	Problematic Facebook use has been linked to the negative psychological effects of Facebook use associated with perceived loneliness (Primi et al., 2021; Ho et al., 2021a).	
Perceived Eating Disorder	Studies	González-Nuevo et al. (2021) [+][∼]; Hummel and Smith (2015) [−][∼]; Mabe et al. (2014) [−][∼]; Mannino et al. (2021) [−][+][∼]; Puccio et al. (2016) [−][+]; Smith et al. (2013) [−]; Walker et al. (2015) [−][+][∼]	
	Descriptive Information	Total number of studies	7
		Number of studies reporting a negative effect	6
		Number of studies reporting a positive effect	4
		Number of studies reporting no effect	5
		Number of descriptive studies	0
	Negative Effects	Duration of Facebook use (Mabe et al., 2014), maladaptive Facebook use (Mannino et al., 2021; Smith et al., 2013), passive use of Facebook for social comparison (Mannino et al., 2021), perceived negative feedback seeking on Facebook (Hummel and Smith, 2015), perceived online physical appearance comparison (Walker et al., 2015), perceived social comparison on Facebook (Puccio et al., 2016), personal status updates on Facebook (Hummel and Smith, 2015), and time spent on Facebook (Mannino et al., 2021)	
	Positive Effects	Facebook intensity (Walker et al., 2015), general Facebook use (González-Nuevo et al., 2021), passive use of Facebook for social connection (Mannino et al., 2021), and perceived social comparison on Facebook (Puccio et al., 2016)	
	No Effects	Facebook intensity (Walker et al., 2015), general Facebook use (González-Nuevo et al., 2021), maladaptive Facebook use (Mannino et al., 2021), passive use of Facebook for social comparison (Mannino et al., 2021), passive use of Facebook for social connection (Mannino et al., 2021), perceived negative feedback seeking on Facebook (Hummel and Smith, 2015), personal status updates on Facebook (Hummel and Smith, 2015), and time spent on Facebook (Mabe et al., 2014; Mannino et al., 2021)	
	Example	Maladaptive Facebook use has been linked to the negative psychological effects of Facebook use associated with perceived eating disorders (Smith et al., 2013).	
Perceived Self-Esteem	Studies	Ahamed et al. (2021) [−][∼]; Alfasi (2019) [−]; Atroszko et al. (2018) [−]; Awobamise et al. (2022) [−]; Bais and Reyes (2020) [−]; Baturay and Toker (2017) [−][∼]; Bergagna and Tartaglia (2018) [−][∼]; Błachnio and Przepiórka (2019) [−]; Błachnio et al. (2016c) [−]; Błachnio et al. (2016d) [−][+][∼]; Błachnio et al. (2019) [−]; Brailovskaia and Margraf (2016) [+]; Castillo de Mesa et al. (2020) [+][∼]; Chen and Lee (2013) [−]; Cingel and Olsen (2018) [−][∼]; Cramer et al. (2016) [−][∼]; Cudo et al. (2020a) [∼]; Cury et al. (2022) [/]; Errasti et al. (2017) [−][∼]; Eşkisu et al. (2020) [−]; Eşkisu et al. (2017) [−][∼]; Faraon and Kaipainen (2014) [−][∼]; Flynn et al. (2018) [−][∼]; Goljović (2017) [−]; Gonzales and Hancock (2011) [+]; Hanna et al. (2017) [−][∼]; Hong et al. (2014) [−][∼]; Hussain et al. (2019) [∼]; Jenkins-Guarnieri et al. (2012) [∼]; Jang et al. (2016) [∼]; Kalpidou et al. (2011) [∼]; Kanat-Maymon et al. (2018) [−]; Lee et al. (2012) [∼]; Lee (2020) [−]; Lee (2014) [−]; Longua Peterson et al. (2017) [−][∼]; Malik and Khan (2015) [−]; Manago et al. (2012) [∼]; Marengo et al. (2021) [+][∼]; Metzler and Scheithauer (2017) [+][∼]; Michikyan et al. (2015) [−][+][∼]; Nizami et al. (2017) [−]; O’Sullivan and Hussain (2017) [+][∼]; Omolayo et al. (2013) [+]; Ozimek and Bierhoff (2020) [−][+][∼]; Ozimek et al. (2021) [−][∼]; Primi et al. (2021) [−]; Przepiórka et al. (2021) [−]; Schmuck et al. (2019) [∼]; Sehar et al. (2022) [+]; Seran et al. (2020) [−]; Skues et al. (2012) [∼]; Smith and Short (2022) [−]; Soraci et al. (2020) [∼]; Stănculescu and Griffiths (2021) [−]; Stieger (2019) [−][∼]; Tazghini and Siedlecki (2013) [−][+][∼]; Tobin and Graham (2020) [−]; Triệu et al. (2021) [/]; Türkmen et al. (2022) [∼]; Uram and Skalski (2022) [−]; Uttravanich and Blauw (2018) [∼]; Vogel et al. (2015) [∼]; Vogel et al. (2014) [−]; Whitman and Gottdiener (2016) [+]; Wright et al. (2018) [+][∼]; Ye et al. (2021) [∼];	
	Descriptive Information	Total number of studies	67
		Number of studies reporting a negative effect	41
		Number of studies reporting a positive effect	14
		Number of studies reporting no effect	37
		Number of descriptive studies	2
	Negative Effects	Acquaintance as intended purpose for using Facebook (Eşkisu et al., 2017), browsing own Facebook newsfeed (Alfasi, 2019), compare/impress as motive for false self-presentation on Facebook (Michikyan et al., 2015), compensatory Facebook use (Goljović, 2017), deception as motive for false self-presentation on Facebook (Michikyan et al., 2015), exploration as motive for false self-presentation on Facebook (Michikyan et al., 2015), Facebook addiction (Atroszko et al., 2018; Awobamise et al., 2022; Bais and Reyes, 2020; Baturay and Toker, 2017; Błachnio et al., 2016c; Eşkisu et al., 2020; Goljović, 2017; Hong et al., 2014; Kanat-Maymon et al., 2018; Malik and Khan, 2015; Nizami et al., 2017; Seran et al., 2020; Smith and Short, 2022; Stănculescu and Griffiths, 2021; Uram and Skalski, 2022), Facebook fatigue (Cramer et al., 2016), Facebook intensity (Błachnio et al., 2016c; Ahamed et al., 2021), Facebook intrusion (Błachnio et al., 2019; Błachnio and Przepiórka, 2019; Przepiórka et al., 2021), frequency of Facebook use (Vogel et al., 2014; Kanat-Maymon et al., 2018), general Facebook use (Cingel and Olsen, 2018), ideal self-presentation on Facebook (Michikyan et al., 2015), interaction on Facebook (Chen and Lee, 2013), nightly time spent on Facebook (Longua Peterson et al., 2017), passive Facebook use (Hanna et al., 2017), perceived downward social comparison on Facebook (Vogel et al., 2014), perceived feeling of connectedness to Facebook (Tazghini and Siedlecki, 2013), perceived frequency of having a negative feeling from social comparison on Facebook (Lee, 2014), perceived frequency of posting information and updating Facebook page (Errasti et al., 2017), perceived frequency of social comparison on Facebook (Lee, 2014), perceived frequency of untagging oneself from in photos on Facebook (Tazghini and Siedlecki, 2013), perceived level of accepting friend requests from unknown people on Facebook (Tazghini and Siedlecki, 2013), perceived level of activity on Facebook (Ozimek et al., 2021), perceived level of belief that Facebook is too invasive (Tazghini and Siedlecki, 2013), perceived level of easier communication on Facebook (Tazghini and Siedlecki, 2013), perceived level of Facebook integration into daily activities (Faraon and Kaipainen, 2014), perceived level of Facebook integration into daily routines (Faraon and Kaipainen, 2014), perceived level of impressing on Facebook (Ozimek et al., 2021), perceived level of social comparison perception on Facebook (Cramer et al., 2016), perceived level of watching on Facebook (Ozimek and Bierhoff, 2020; Ozimek et al., 2021), perceived negative activities on Facebook (Tazghini and Siedlecki, 2013), perceived social comparison on Facebook (Flynn et al., 2018; Alfasi, 2019), perceived upward social comparison on Facebook (Lee, 2020; Vogel et al., 2014), personal importance of Facebook use (Błachnio et al., 2016d), problematic Facebook use (Tobin and Graham, 2020; Primi et al., 2021), risky and impulsive Facebook use (Flynn et al., 2018), text contribution on Facebook (Cingel and Olsen, 2018), time spent on Facebook (Faraon and Kaipainen, 2014; Hanna et al., 2017; Bergagna and Tartaglia, 2018), time spent on Facebook for private purposes (Stieger, 2019), use of Facebook for new acquaintance (Tazghini and Siedlecki, 2013), use of Facebook for simulation (Bergagna and Tartaglia, 2018), and use of Facebook for social comparison (Ozimek and Bierhoff, 2020)	
	Positive Effects	Facebook addiction (Sehar et al., 2022), Facebook intensity (Whitman and Gottdiener, 2016), general Facebook use (Omolayo et al., 2013; Brailovskaia and Margraf, 2016), initiating of online relationships on Facebook (Metzler and Scheithauer, 2017), instrumental Facebook use (Błachnio et al., 2016d), intensity of receiving positive feedback on Facebook (Marengo et al., 2021), liking behavior on Facebook (Wright et al., 2018), number of Facebook friends (Metzler and Scheithauer, 2017), perceived level of activity on Facebook (Ozimek and Bierhoff, 2020), perceived level of happiness on personal Facebook page (Tazghini and Siedlecki, 2013), perceived level of impressing on Facebook (Ozimek and Bierhoff, 2020), positive self-presentation on Facebook (Metzler and Scheithauer, 2017), real self-presentation on Facebook (Michikyan et al., 2015), strategic digital skills on Facebook (Castillo de Mesa et al., 2020), temporary break from Facebook use (O’Sullivan and Hussain, 2017), use of socially interactive features of Facebook (Błachnio et al., 2016d), and viewing own Facebook profile (Gonzales and Hancock, 2011)	
	No Effects	Actions toward maintaining relations on Facebook (Castillo de Mesa et al., 2020), active Facebook use (Hanna et al., 2017), active hours on Facebook (Baturay and Toker, 2017), connection as motive for using Facebook (Tazghini and Siedlecki, 2013), digital skills on Facebook (Castillo de Mesa et al., 2020), education as intended purpose for using Facebook (Eşkisu et al., 2017), expression of empathy regarding the emotions of others on Facebook (Errasti et al., 2017), expression of personal emotions on Facebook (Errasti et al., 2017), Facebook access time via PC (Ye et al., 2021), Facebook access time via smartphone (Ye et al., 2021), Facebook account length (Hussain et al., 2019; Lee et al., 2012), Facebook addiction (Hussain et al., 2019; Soraci et al., 2020), Facebook intensity (Jenkins-Guarnieri et al., 2012; Błachnio et al., 2016d; O’Sullivan and Hussain, 2017; Ahamed et al., 2021), Facebook intrusion (Cudo et al., 2020a), Facebook network size (Manago et al., 2012), Facebook session length (Hussain et al., 2019), frequency of Facebook updates (Marengo et al., 2021), frequency of Facebook use (Cudo et al., 2020a; Türkmen et al., 2022), frequency of receiving positive feedback on Facebook (Marengo et al., 2021), general Facebook use (Cramer et al., 2016; Eşkisu et al., 2017; Jang et al., 2016; Uttravanich and Blauw, 2018), information search on Facebook (Castillo de Mesa et al., 2020), initiating of online relationships on Facebook (Metzler and Scheithauer, 2017), inspection time of Facebook updates (Hussain et al., 2019), inspection time of social updates on Facebook (Hussain et al., 2019), lying behavior on Facebook (Wright et al., 2018), mobile Facebook use (Schmuck et al., 2019), nightly time spent on updating Facebook status (Longua Peterson et al., 2017), number of Facebook friends (Cingel and Olsen, 2018; Errasti et al., 2017; Eşkisu et al., 2017; Faraon and Kaipainen, 2014; Flynn et al., 2018; Kalpidou et al., 2011; Lee et al., 2012; Skues et al., 2012; Stieger, 2019), number of Facebook logins (Skues et al., 2012), passive engagement on Facebook (Cingel and Olsen, 2018), perceived appearance self-esteem state (Ozimek et al., 2021), perceived frequency of commenting on statuses on Facebook (Tazghini and Siedlecki, 2013), perceived frequency of posting on Facebook (Tazghini and Siedlecki, 2013; Ye et al., 2021), perceived frequency of posting YouTube clips on Facebook (Tazghini and Siedlecki, 2013), perceived frequency of putting a lot of thought into one posts on Facebook (Tazghini and Siedlecki, 2013), perceived frequency of tagging people in statuses on Facebook (Tazghini and Siedlecki, 2013), perceived frequency of updating Facebook status (Eşkisu et al., 2017), perceived level of acting on Facebook (Ozimek and Bierhoff, 2020; Ozimek et al., 2021), perceived level of activity on Facebook (Michikyan et al., 2015), perceived level of awareness when using Facebook (Tazghini and Siedlecki, 2013), perceived level of clicking “like” on photos on Facebook (Tazghini and Siedlecki, 2013), perceived level of Facebook dependency (Lee et al., 2012), perceived level of feeling judged by what one posts on Facebook (Tazghini and Siedlecki, 2013), perceived level of free expression on Facebook (Tazghini and Siedlecki, 2013), perceived level of networking on Facebook (Tazghini and Siedlecki, 2013), perceived level of posting activities, feelings, or photos on Facebook (Tazghini and Siedlecki, 2013), perceived level of praise on Facebook (Tazghini and Siedlecki, 2013), perceived level of regret if Facebook shuts down (Tazghini and Siedlecki, 2013), perceived level of social comparison activity on Facebook (Cramer et al., 2016), perceived level spending more time viewing a person’s Facebook page than commenting (Tazghini and Siedlecki, 2013), perceived performance self-esteem state (Ozimek et al., 2021), perceived self-esteem state (Ozimek et al., 2021), perceived social comparison orientation on Facebook (Jang et al., 2016; Vogel et al., 2015), perceived social self-esteem state (Ozimek et al., 2021), positive self-presentation on Facebook (Metzler and Scheithauer, 2017), posting on Facebook (Cramer et al., 2016), prevalence of self-generated content in Facebook update (Marengo et al., 2021), private communication with Facebook friends (Manago et al., 2012), public communication with Facebook friends (Manago et al., 2012), reading on Facebook (Cramer et al., 2016), social interaction as intended purpose for using Facebook (Eşkisu et al., 2017), strategic digital skills on Facebook (Castillo de Mesa et al., 2020), time spent on Facebook (Eşkisu et al., 2017; Flynn et al., 2018; Kalpidou et al., 2011; Michikyan et al., 2015; Skues et al., 2012), time spent on Facebook apps (including apps) (Hong et al., 2014), time spent on Facebook chat rooms (Hong et al., 2014), time spent on Facebook newsfeeds (Hong et al., 2014), tolerance of diversity on Facebook (Castillo de Mesa et al., 2020), use and presence of Facebook in life (Castillo de Mesa et al., 2020), use of Facebook for search for relations (Bergagna and Tartaglia, 2018), use of Facebook for social interaction (Bergagna and Tartaglia, 2018), and visual contribution on Facebook (Cingel and Olsen, 2018)	
	Example	Facebook intrusion has been linked to the negative psychological effects of Facebook use associated with perceived self-esteem (Błachnio and Przepiórka, 2019; Błachnio et al., 2019; Przepiórka et al., 2021).	
Perceived Life Satisfaction	Studies	Adnan and Mavi (2015) [+][∼]; Akın and Akın (2015) [−]; Basilisco and Cha (2015) [+]; Biolcati et al. (2018) [−]; Błachnio and Przepiórka (2018) [∼]; Błachnio and Przepiórka (2019) [∼]; Błachnio et al. (2016c) [−][∼]; Błachnio et al. (2019) [−][+][∼]; Brailovskaia and Margraf (2016) [+]; Brailovskaia et al. (2020b) [+]; Castillo de Mesa et al. (2020) [∼]; Chen and Bello (2017) [∼]; Choi (2022) [+][∼]; Cudo et al. (2020b) [+][∼]; Dempsey et al. (2019) [∼]; Frison and Eggermont (2016b) [−][∼]; Gerson et al. (2016) [−]; Giagkou et al. (2018) [∼]; Goljović (2017) [−][∼]; Grieve et al. (2013) [+]; Hu et al. (2017) [+]; Kang et al. (2013) [−][+][∼]; Kross et al. (2013) [∼]; Lee (2020) [+]; Locatelli et al. (2012) [∼]; Lönnqvist and große Deters (2016) [+][∼]; Manago et al. (2012) [+][∼]; Masciantonio et al. (2021) [−][+][∼]; Nabi et al. (2013) [+]; Park and Baek (2018) [∼]; Rae and Lonborg (2015) [∼]; Satici and Uysal (2015) [−]; Satici (2019) [−]; Schmuck et al. (2019) [∼]; Shakya and Christakis (2017) [∼]; Srivastava (2015) [+][∼]; Stieger (2019) [−][∼]; Tromholt (2016) [+]; Uram and Skalski (2022) [∼]; Valenzuela et al. (2009) [+]; Vigil and Wu (2015) [−][+][∼]; Wang (2013) [+]; Wenninger et al. (2014) [−][+][∼]; Zhang (2017) [+][∼]	
	Descriptive Information	Total number of studies	44
		Number of studies reporting a negative effect	14
		Number of studies reporting a positive effect	22
		Number of studies reporting no effect	29
		Number of descriptive studies	0
	Negative Effects	Compensatory Facebook Use (Goljović, 2017), Facebook addiction (Akın and Akın, 2015; Biolcati et al., 2018; Satici, 2019), Facebook intensity (Błachnio et al., 2016c), Facebook intrusion (Błachnio et al., 2019), looking at other’s photos/videos on Facebook (Vigil and Wu, 2015), passive Facebook use (Frison and Eggermont, 2016b), passive following on Facebook (Wenninger et al., 2014), perceived downward-identification in social comparison on Facebook (Kang et al., 2013), perceived negative social comparison on Facebook (Frison and Eggermont, 2016b), perceived social comparison on Facebook (Gerson et al., 2016), perceived upward social comparison on Facebook (Lee, 2020; Masciantonio et al., 2021), perceived upward-contrast in social comparison on Facebook (Kang et al., 2013), problematic Facebook use (Satici and Uysal, 2015), tagging photos on Facebook (Vigil and Wu, 2015), time spent looking at others’ photos/videos on Facebook (Vigil and Wu, 2015), time spent on Facebook (Frison and Eggermont, 2016b; Stieger, 2019; Vigil and Wu, 2015), time spent tagging photos on Facebook (Vigil and Wu, 2015), uploading photos on Facebook (Vigil and Wu, 2015), and use of Facebook chat (Vigil and Wu, 2015)	
	Positive Effects	Active Facebook use (Choi, 2022), Facebook check-in intensity (Wang, 2013), Facebook intensity (Hu et al., 2017; Valenzuela et al., 2009), Facebook intrusion (Błachnio et al., 2019), Facebook network size (Manago et al., 2012), general Facebook use (Basilisco and Cha, 2015; Brailovskaia and Margraf, 2016; Srivastava, 2015), number of Facebook friends (Lönnqvist and große Deters, 2016; Nabi et al., 2013; Srivastava, 2015; Vigil and Wu, 2015), number of Facebook hours per week (Cudo et al., 2020b), perceived enacted social support on Facebook (Zhang, 2017), perceived social attention on Facebook (Adnan and Mavi, 2015), perceived social connectedness from the use of Facebook (Grieve et al., 2013), perceived social support on Facebook (Masciantonio et al., 2021; Zhang, 2017), perceived upward social comparison on Facebook (Lee, 2020), perceived upward-identification in social comparison on Facebook (Kang et al., 2013), posting on Facebook (Wenninger et al., 2014), reduction in time spent on Facebook (Brailovskaia, Ströse, et al., 2020b), shared identity as motive for using Facebook (Adnan and Mavi, 2015), sharing information on Facebook (Wang, 2013), temporary absence from Facebook (Tromholt, 2016), and use of Facebook chat (Wenninger et al., 2014)	
	No Effects	Actions toward maintaining relations on Facebook (Castillo de Mesa et al., 2020), active Facebook use (Masciantonio et al., 2021), commenting on Facebook (Wenninger et al., 2014), communication as motive for using Facebook (Adnan and Mavi, 2015), connection as motive for using Facebook (Rae and Lonborg, 2015), digital skills on Facebook (Castillo de Mesa et al., 2020), entertainment as motive for using Facebook (Adnan and Mavi, 2015), Facebook account length (Kang et al., 2013; Locatelli et al., 2012), Facebook addiction (Błachnio et al., 2016c; Goljović, 2017; Uram and Skalski, 2022), Facebook intrusion (Błachnio and Przepiórka, 2018, 2019; Błachnio et al., 2019), Facebook network size (Zhang, 2017), frequency of Facebook use (Dempsey et al., 2019; Kang et al., 2013), friendship as motive for using Facebook (Rae and Lonborg, 2015), general Facebook use (Kross et al., 2013; Shakya and Christakis, 2017; Srivastava, 2015; Stieger, 2019), information as motive for using Facebook (Adnan and Mavi, 2015; Rae and Lonborg, 2015), information search on Facebook (Castillo de Mesa et al., 2020), level of interest in Facebook use (Kang et al., 2013), liking on Facebook (Wenninger et al., 2014), mobile Facebook use (Schmuck et al., 2019), number of Facebook friends (Adnan and Mavi, 2015; Locatelli et al., 2012; Lönnqvist and große Deters, 2016; Srivastava, 2015; Vigil and Wu, 2015; Wenninger et al., 2014), passive Facebook use (Choi, 2022; Frison and Eggermont, 2016b; Masciantonio et al., 2021),passive following on Facebook (Giagkou et al., 2018), perceived ability-based social comparison orientation on Facebook (Park and Baek, 2018), perceived downward-contrast in social comparison on Facebook (Kang et al., 2013), perceived frequency of writing negative status updates on Facebook (Locatelli et al., 2012), perceived frequency of writing positive status updates on Facebook (Locatelli et al., 2012), perceived frequency of writing status updates on Facebook (Locatelli et al., 2012), perceived opinion-based social comparison orientation on Facebook (Park and Baek, 2018), perceived social support provided on Facebook (Chen and Bello, 2017), perceived social support received on Facebook (Chen and Bello, 2017), private communication with Facebook friends (Manago et al., 2012), problematic Facebook use (Cudo, Wojtasiński, et al., 2020; Dempsey et al., 2019), public communication with Facebook friends (Manago et al., 2012), social investigation as motive for using Facebook (Adnan and Mavi, 2015), strategic digital skills on Facebook (Castillo de Mesa et al., 2020), time spent on Facebook (Adnan and Mavi, 2015; Kang et al., 2013; Locatelli et al., 2012; Zhang, 2017), tolerance of diversity on Facebook (Castillo de Mesa et al., 2020), and use and presence of Facebook in life (Castillo de Mesa et al., 2020)	
	Example	Time spent on Facebook has been linked to the negative psychological effects of Facebook use associated with perceived life satisfaction (Frison and Eggermont, 2016b; Stieger, 2019; Vigil and Wu, 2015).	
Perceived Insomnia	Studies	Atroszko et al. (2018) [−]; Błachnio et al. (2021) [−]; Brailovskaia et al. (2019a) [−]; Brailovskaia et al. (2019b) [−][∼]; Hanprathet et al. (2015) [/]; Ho (2021a) [−]; Ho (2021b) [−]; Ho et al. (2021a) [−]; Ho et al. (2021b) [−]; Hosen et al. (2021) [−]; Jha et al. (2016) [/]; Koc and Gulyagci (2013) [−][∼]; Przepiórka and Błachnio (2020) [−]; Rahman and Zakaria (2021) [−]; Wang et al. (2021) [−]; Wolniczak et al. (2013) [−]	
	Descriptive Information	Total number of studies	16
		Number of studies reporting a negative effect	14
		Number of studies reporting a positive effect	0
		Number of studies reporting no effect	2
		Number of descriptive studies	2
	Negative Effects	Daily Facebook use (Brailovskaia et al., 2019b), duration of daily Facebook use (Brailovskaia et al., 2019a), Facebook addiction (Koc and Gulyagci, 2013; Atroszko et al., 2018; Brailovskaia et al., 2019b; Wang et al., 2021; Ho, 2021a,b; Ho et al., 2021b), Facebook dependence (Wolniczak et al., 2013), Facebook intrusion (Błachnio et al., 2021; Przepiórka and Błachnio, 2020), general Facebook use (Brailovskaia et al., 2019a), problematic Facebook use (Ho et al., 2021a), and time spent on Facebook (Hosen et al., 2021; Rahman and Zakaria, 2021)	
	Positive Effects	N/A	
	No Effects	Academic motive for using Facebook (Koc and Gulyagci, 2013), daily Facebook use (Brailovskaia et al., 2019b), daily informational motive for using Facebook (Koc and Gulyagci, 2013), Facebook addiction (Brailovskaia et al., 2019b), social motive for using Facebook (Koc and Gulyagci, 2013), and weekly time commitment on Facebook (Koc and Gulyagci, 2013)	
	Example	Facebook intrusion has been linked to the negative psychological effects of Facebook use associated with perceived insomnia (Błachnio et al., 2021).	
Perceived Stress	Studies	Atroszko et al. (2018) [−]; Atroszko et al. (2022) [−]; Balcerowska et al. (2022) [−]; Bevan et al. (2014) [∼]; Brailovskaia and Margraf (2016) [∼]; Brailovskaia and Margraf (2017) [−][∼]; Brailovskaia et al. (2019a) [−]; Brailovskaia et al. (2019c) [−]; Brailovskaia et al. (2018b) [−]; Brailovskaia et al. (2019d) [−]; Çakıcı et al. (2020) [−]; Eşkisu et al. (2020) [−]; Farahani et al. (2011) [−]; Flynn et al. (2018) [−][∼]; Frison and Eggermont (2015) [∼]; Ho (2021b) [−]; Ho et al. (2021a) [−]; Hussain et al. (2019) [∼]; Labrague (2014) [∼]; Luqman et al. (2017) [−]; Nabi et al. (2013) [+]; Nasser et al. (2019) [−]; Nazzal et al. (2021) [−]; O’Sullivan and Hussain (2017) [−]; Pal et al. (2018) [−]; Verseillié et al. (2021) [−]; Wright et al. (2018) [−][∼]	
	Descriptive Information	Total number of studies	27
		Number of studies reporting a negative effect	21
		Number of studies reporting a positive effect	1
		Number of studies reporting no effect	8
		Number of descriptive studies	0
	Negative Effects	Excessive cognitive use of Facebook (Luqman et al., 2017), excessive hedonic use of Facebook (Luqman et al., 2017), excessive social use of Facebook (Luqman et al., 2017), Facebook addiction (Brailovskaia and Margraf, 2017; Atroszko et al., 2018, 2022; Brailovskaia et al., 2018a,b, 2019c,d; Çakıcı et al., 2020; Eşkisu et al., 2020; Verseillié et al., 2021; Ho, 2021b; Balcerowska et al., 2022), Facebook intensity (O’Sullivan and Hussain, 2017; Pal et al., 2018; Brailovskaia et al., 2019c; Nazzal et al., 2021), general Facebook use (Farahani et al., 2011; Brailovskaia et al., 2019a), liking behavior on Facebook (Wright et al., 2018), number of Facebook friends (Nazzal et al., 2021), perceived emotional engagement with Facebook (Verseillié et al., 2021), perceived online social support received from other Facebook users (Brailovskaia et al., 2019c), perceived social comparison on Facebook (Flynn et al., 2018), problematic Facebook use (Nasser et al., 2019; Ho et al., 2021a), risky and impulsive Facebook use (Flynn et al., 2018), and time spent on Facebook (Flynn et al., 2018; Nazzal et al., 2021)	
	Positive Effect	Number of Facebook friends (Nabi et al., 2013)	
	No Effects	Facebook account length (Bevan et al., 2014; Hussain et al., 2019), Facebook addiction (Brailovskaia and Margraf, 2017; Hussain et al., 2019), Facebook intensity (Labrague, 2014), Facebook session length (Hussain et al., 2019), general Facebook use (Brailovskaia and Margraf, 2016), inspection time of Facebook updates (Hussain et al., 2019), inspection time of social updates on Facebook (Hussain et al., 2019), lying behavior on Facebook (Wright et al., 2018), number of Facebook friends (Bevan et al., 2014; Labrague, 2014; Flynn et al., 2018), perceived social support seeking through Facebook (Frison and Eggermont, 2015), perceived social support through Facebook (Frison and Eggermont, 2015), temporary break from Facebook use (O’Sullivan and Hussain, 2017), and time spent on Facebook (Bevan et al., 2014; Labrague, 2014)	
	Example	Problematic Facebook use has been linked to the negative psychological effects of Facebook use associated with perceived stress (Nasser et al., 2019; Ho et al., 2021a).	

#### 3.1.1. Perceived anxiety

Forty-seven studies were found that examined the psychological effects of Facebook use on perceived (social) anxiety. Results varied widely, ranging from no effect to a strong effect. The 47 studies included 43 cross-sectional studies (42 surveys and 1 case–control survey), 2 longitudinal studies (2 panel studies), 1 experimental study (1 quasi-experiment), and 1 study that applied a multimethod research design (1 study was a longitudinal panel study and another one an experimental study with a randomized controlled trial (RCT) design).

The results of the review revealed that Facebook addiction was slightly to strongly positively correlated with perceived (social) anxiety (Koc and Gulyagci, 2013; Zaffar et al., 2015; Brailovskaia and Margraf, 2017; Atroszko et al., 2018, 2022; da Veiga et al., 2019; Foroughi et al., 2019; Louragli et al., 2019; Sotero et al., 2019; Xie and Karan, 2019; Eşkisu et al., 2020; Brailovskaia et al., 2020a,b; Verseillié et al., 2021). Results also suggest that individuals with Facebook addiction are at high risk of developing anxiety (Hanprathet et al., 2015). Further examples of positive effects on perceived (social) anxiety include, for example, Facebook intrusion (Błachnio et al., 2021), lying and liking behavior on Facebook (Wright et al., 2018), number of Facebook friends (Flynn et al., 2018; Nazzal et al., 2021), perceived emotional connectedness to Facebook (Clayton et al., 2013), perceived emotional engagement with Facebook (Verseillié et al., 2021), risky and impulsive Facebook use (Flynn et al., 2018), time spent on Facebook (Labrague, 2014; Shaw et al., 2015; Flynn et al., 2018; Sternberg et al., 2018; Nazzal et al., 2021), and use of socially interactive features of Facebook (McCord et al., 2014). For individuals who make social comparisons on Facebook, which can lead to a perceived frequency of a negative feeling from social comparisons on Facebook (Lee, 2014), there was a medium positive effect for perceived anxiety. Positive correlations with perceived anxiety were also found to a small to moderate extent for users with passive Facebook use (Shaw et al., 2015; Hanna et al., 2017) or problematic Facebook use (Lee-Won et al., 2015; Chabrol et al., 2017; Dempsey et al., 2019; Nasser et al., 2019; Ho et al., 2021a). Examples of negative effects on perceived (social) anxiety are frequency of Facebook use (Dempsey et al., 2019) or perceived social connectedness from the use of Facebook (Grieve et al., 2013).

No statistically significant effect was found between the following types of Facebook use and perceived (social) anxiety, among others: academic motive for using Facebook (Koc and Gulyagci, 2013), active Facebook use (Hanna et al., 2017), connection as motive for using Facebook (Rae and Lonborg, 2015), daily informational motive for using Facebook (Koc and Gulyagci, 2013), Facebook account length (Hussain et al., 2019; Ögel-Balaban and Altan, 2020), friendship as motive for using Facebook (Rae and Lonborg, 2015), information as motive for using Facebook (Rae and Lonborg, 2015), inspection time of Facebook updates (Hussain et al., 2019), inspection time of social updates on Facebook (Hussain et al., 2019), number of activities during Facebook use (Sternberg et al., 2018), perceived frequency of posting on Facebook (Ögel-Balaban and Altan, 2020), social motive for using Facebook (Koc and Gulyagci, 2013), use of Facebook for interactive communication (Shaw et al., 2015), use of socially interactive features of Facebook (McCord et al., 2014; Sillence et al., 2021), and weekly time commitment on Facebook (Koc and Gulyagci, 2013). A summary of all effects of the forty-seven studies that examined the psychological effects of Facebook use on perceived (social) anxiety can be found in Table 2.

#### 3.1.2. Perceived depression

Eighty-nine studies were found that examined the psychological effects of Facebook use on perceived depression. Results varied widely, ranging from no effect to a strong effect. The 89 studies included 76 cross-sectional studies (75 surveys and 1 case–control survey), 10 longitudinal studies (8 panel studies and 2 longitudinal randomized experiments), 2 experimental studies (1 quasi-experiment and 1 experimental study with an RCT design), and 1 study that applied a multimethod research design (1 study was a cross-sectional survey study and another one was a longitudinal study with a time-series design).

Low to high positive effects on perceived depression have been found among individuals who are addicted to Facebook (Koc and Gulyagci, 2013; Hong et al., 2014; Zaffar et al., 2015; Brailovskaia and Margraf, 2017; Khattak et al., 2017; da Veiga et al., 2019; Damota, 2019; Foroughi et al., 2019; Kulkarni and Deshpande, 2019; Sotero et al., 2019; Brailovskaia et al., 2019b,d; Bais and Reyes, 2020; Eşkisu et al., 2020; Iovu et al., 2020; Rachubińska et al., 2021; Verseillié et al., 2021; Ho, 2021a; Atroszko et al., 2022) or through perceived social comparisons on Facebook, such as the perceived upward social comparison on Facebook (Steers et al., 2014; Tosun and Kaşdarma, 2020; Dibb and Foster, 2021). Further positive effects on perceived depression include active private or public Facebook use (Frison and Eggermont, 2016a, 2020), Facebook intensity (Iovu et al., 2020; Ahamed et al., 2021; Nazzal et al., 2021), Facebook intrusion (Bendayan and Blanca Mena, 2019; Przepiórka and Błachnio, 2020; Cudo et al., 2020a,b), Facebook surveillance (Scherr et al., 2019), liking behavior on Facebook (Wright et al., 2018), passive Facebook use (Frison and Eggermont, 2016a, 2020; Dibb and Foster, 2021), perceived negative social support on Facebook (McCloskey et al., 2015), problematic Facebook use (Walburg et al., 2016; Chabrol et al., 2017; Dempsey et al., 2019; Nasser et al., 2019; Ho et al., 2021a), and time spent on Facebook (Kang et al., 2013; Labrague, 2014; Steers et al., 2014; Chow and Wan, 2017; Scherr and Brunet, 2017; Flynn et al., 2018; Sternberg et al., 2018; Frison et al., 2019; Frison and Eggermont, 2020; Nazzal et al., 2021; Yeshua-Katz and Zilberstein, 2021). Also, results suggest that general Facebook use predicts bipolar disorder (Rosen et al., 2013a,b).

Examples of negative effects on perceived depression include perceived social comparison when using Facebook actively (Nisar et al., 2019), perceived social connectedness from the use of Facebook (Grieve et al., 2013), perceived social support through Facebook (Frison and Eggermont, 2015, 2016a; Frison et al., 2019), perceived upward-identification in social comparison on Facebook (Kang et al., 2013), and relationship maintenance as motive for using Facebook (Scherr and Brunet, 2017). The number of Facebook friends, for example, was both negatively (Rae and Lonborg, 2015; Brailovskaia and Margraf, 2019) and positively (Nazzal et al., 2021) associated with perceived depression.

No statistically significant effect was found between the following types of Facebook use and perceived loneliness, among others: Facebook account length (Locatelli et al., 2012; Kang et al., 2013), Facebook network size (Zhang, 2017), Facebook session length (Hussain et al., 2019), level of interest in Facebook use (Kang et al., 2013), lying behavior on Facebook (Wright et al., 2018), number of activities during Facebook use (Sternberg et al., 2018), number of Facebook pages a user has marked as like (Park et al., 2013), number of groups on Facebook for which a user is an administrator (Park et al., 2013), number of groups on Facebook to which a user belongs (including groups of which a user is an administrator) (Park et al., 2013), number of interest items listed on the user’s Facebook profile (Park et al., 2013), number of pending incoming friend requests on Facebook (Park et al., 2013), perceived downward social comparison on Facebook (Dibb and Foster, 2021), perceived frequency of writing in discussion groups on Facebook (Brailovskaia and Margraf, 2019), perceived frequency of writing negative status updates on Facebook (Locatelli et al., 2012), perceived frequency of writing online messages on Facebook (Brailovskaia and Margraf, 2019), perceived frequency of writing positive status updates on Facebook (Locatelli et al., 2012), perceived frequency of writing status updates on Facebook (Locatelli et al., 2012; Brailovskaia and Margraf, 2019), time spent on Facebook apps (including games) (Hong et al., 2014), time spent on Facebook chat rooms (Hong et al., 2014), time spent on Facebook newsfeeds (Hong et al., 2014), and viewing other Facebook profiles as motive for using Facebook (Maglunog and Dy, 2019). A summary of all effects of the eighty-nine studies that examined the psychological effects of Facebook use on perceived depression can be found in Table 2.

#### 3.1.3. Perceived loneliness

Forty-six studies were found that examined the psychological effects of Facebook use on perceived loneliness. Results varied widely, ranging from no effect to a strong effect. The 46 studies included 41 cross-sectional studies (40 surveys) and 5 longitudinal studies (4 panel studies and 1 longitudinal randomized experiment).

Very strong positive effects on perceived loneliness were found for perceived upward social comparison on Facebook (Lim and Yang, 2019; Dibb and Foster, 2021). Also, a positive medium-strong correlation was found between compensatory Facebook use (Goljović, 2017) or connection as motive for using Facebook (Clayton et al., 2013; Jin, 2013) and perceived loneliness. A medium-weak correlation was found between time spent on Facebook (Skues et al., 2012; Lemieux et al., 2013; Teppers et al., 2014; Kumar et al., 2019; Frison and Eggermont, 2020; Rahman and Zakaria, 2021) and perceived loneliness. Furthermore, Facebook addiction correlates positively with perceived loneliness to a low to moderate level (Omar and Subramanian, 2013; Saleem et al., 2016; Błachnio et al., 2016a; Chavez and Chavez Jr., 2017; Goljović, 2017; Shettar et al., 2017; Atroszko et al., 2018; Biolcati et al., 2018; Satici, 2019; Aung and Tin, 2020; Rajesh and Rangaiah, 2020; Ho et al., 2021a Ho, 2021a; Smith and Short, 2022). However, Rachubińska et al. (2021) also found a negative correlation between Facebook addiction and perceived loneliness.

A negative effect was found between the number of Facebook friends and perceived loneliness (Skues et al., 2012; Jin, 2013; Phu and Gow, 2019). That is, the more Facebook friends one has, the lower the feeling of perceived loneliness. Results also indicate that active use of Facebook (Jin, 2013), including connection (Clayton et al., 2013; Jin, 2013), maintaining relationships (Teppers et al., 2014), or personal contact (Teppers et al., 2014) as motive for using Facebook can reduce perceived loneliness. Also, results suggest that active posting on Facebook can reduce perceived loneliness (große Deters and Mehl, 2013).

No statistically significant effect was found between the following types of Facebook use and perceived depression, among others: communication as motive for using Facebook (Aydın et al., 2013), Facebook access time via PC (Ye et al., 2021), Facebook access time via smartphone (Ye et al., 2021), following photos, videos, status, comments as motive for using Facebook (Aydın et al., 2013), frequency of Facebook use (Türkmen et al., 2022), new acquaintance as motive for using Facebook (Aydın et al., 2013), number of Facebook logins (Skues et al., 2012), passive engagement on Facebook (Ryan and Xenos, 2011), perceived boredom of use of Facebook (Phu and Gow, 2019), perceived downward social comparison on Facebook (Dibb and Foster, 2021), perceived use experience of Facebook (Jin, 2013), personal contact as motive for using Facebook (Teppers et al., 2014), playing games on Facebook as motive for using Facebook (Aydın et al., 2013), sharing photos, videos, and notifications on Facebook as motive for using Facebook (Aydın et al., 2013), time spent on Facebook for private purposes (Stieger, 2019), use of Facebook chat (Ahmed, 2018), and use of Facebook for news and information (Ryan and Xenos, 2011). A summary of all effects of the forty-six that examined the psychological effects of Facebook use on perceived loneliness can be found in Table 2.

#### 3.1.4. Perceived eating disorder

Seven studies were found that examined the psychological effects of Facebook use on perceived eating disorder. Results varied widely, ranging from no effect to a strong effect. The 7 studies included 4 longitudinal studies (4 panel studies), 2 cross-sectional studies (2 surveys), and 1 study that applied a multimethod research design (1 study was a cross-sectional survey study and another one was a matched-pair experimental study).

Maladaptive Facebook use was found to be a significant predictor of increases in perceived bulimic symptoms, perceived body dissatisfaction, perceived shape concerns, and perceived episodes of overeating (Smith et al., 2013). Results further indicate that maladaptive Facebook use had moderately strong positive effects on perceived concern about physical shape and weight (Mannino et al., 2021). When Facebook was used to make online comparisons of physical appearance, it had large effects on perceived eating disorder, which means the more comparisons, the more likely the perceived eating disorder (Walker et al., 2015). Perceptions of social comparison on Facebook also correlated significantly positively with perceived food restraint and perceived bulimic symptoms, although perceptions of social comparison on Facebook suggested that perceived bulimic symptoms decreased over time (Puccio et al., 2016). Passive use of Facebook for social comparison (Mannino et al., 2021), perceived negative feedback seeking on Facebook (Hummel and Smith, 2015), personal status updates on Facebook (Hummel and Smith, 2015), and time spent on Facebook (Mannino et al., 2021) showed little to no effect on perceived physical shape concern, perceived concern about weight, or perceived concern about eating. Individuals who spent 20 min on Wikipedia showed greater decreases in perceived concerns about weight and shape than those individuals who spent 20 min on Facebook (Mabe et al., 2014).

Facebook use was not significantly related to the “Eating Attitudes Test-26 (EAT-26)” (González-Nuevo et al., 2021), a screening instrument for eating disorders, dieting, and bulimia (Garner et al., 1982). Similarly, perceived negative feedback seeking on Facebook (Hummel and Smith, 2015) was not associated with perceived dietary restraint (Hummel and Smith, 2015). Also, time spent on Facebook did not significantly correlate with disordered eating behaviors (Mabe et al., 2014). A summary of all effects of the seven that examined the psychological effects of Facebook use on perceived eating disorder can be found in Table 2.

#### 3.1.5. Perceived self-esteem

Sixty-seven studies were found that examined the psychological effects of Facebook use on perceived self-esteem. Results varied widely, ranging from no effect to a strong effect. The 67 studies included 58 cross-sectional studies (57 surveys and 1 case–control survey), 4 experimental studies (3 experimental studies with an RCT design and 1 quasi-experiment), 3 longitudinal studies (2 panel studies and 1 longitudinal study with a time-series design), and 2 studies that conducted a multimethod research design (specifically a cross-sectional survey study with an experimental study with an RCT design).

Perceptions of social comparison on Facebook, especially perceived upward social comparison on Facebook (Vogel et al., 2014; Lee, 2020) and perceived frequency of a negative feeling from social comparisons on Facebook (Lee, 2014) had a strong negative effect on perceived self-esteem (Lee, 2014, 2020). Facebook addiction also had a particularly negative effect on perceived self-esteem (Hong et al., 2014; Malik and Khan, 2015; Błachnio et al., 2016b; Baturay and Toker, 2017; Goljović, 2017; Nizami et al., 2017; Atroszko et al., 2018; Kanat-Maymon et al., 2018; Bais and Reyes, 2020; Eşkisu et al., 2020; Seran et al., 2020; Stănculescu and Griffiths, 2021; Awobamise et al., 2022; Smith and Short, 2022; Uram and Skalski, 2022). However, different results could be found in this regard. Namely, Sehar et al. (2022) found a strong positive relationship between Facebook addiction and perceived self-esteem. Facebook intensity also had a positive (Whitman and Gottdiener, 2016) and negative (Błachnio et al., 2016c; Ahamed et al., 2021) effect on perceived self-esteem. Further examples of negative effects on perceived self-esteem include compensatory Facebook use (Goljović, 2017), Facebook fatigue (Cramer et al., 2016), Facebook intrusion (Błachnio et al., 2019; Błachnio and Przepiórka, 2019; Przepiórka et al., 2021), perceived feeling of connectedness to Facebook (Tazghini and Siedlecki, 2013), perceived frequency of untagging oneself from in photos on Facebook (Tazghini and Siedlecki, 2013), perceived level of Facebook integration into daily activities (Faraon and Kaipainen, 2014), perceived negative activities on Facebook (Tazghini and Siedlecki, 2013), problematic Facebook use (Tobin and Graham, 2020; Primi et al., 2021), risky and impulsive Facebook use (Flynn et al., 2018), time spent on Facebook (Faraon and Kaipainen, 2014; Hanna et al., 2017; Bergagna and Tartaglia, 2018), and use of Facebook for simulation (Bergagna and Tartaglia, 2018). Research also suggests that browsing own Facebook newsfeed (Alfasi, 2019), passive Facebook use (Hanna et al., 2017), and use of Facebook for social comparison (Ozimek and Bierhoff, 2020) are associated with lower perceived self-esteem.

Positive effects on perceived self-esteem included, for example, initiating of online relationships as motive for using Facebook (Metzler and Scheithauer, 2017), liking behavior on Facebook (Wright et al., 2018), number of Facebook friends (Metzler and Scheithauer, 2017), temporary break from Facebook use (O’Sullivan and Hussain, 2017), or use of socially interactive features of Facebook (Błachnio et al., 2016d), Facebook users had significantly higher mean score for perceived self-esteem compared to non-Facebook users (Brailovskaia and Margraf, 2016). Individuals who viewed only their own profile reported higher self-esteem than those who viewed other profiles in addition to their own (Gonzales and Hancock, 2011).

No statistically significant effect was found between the following types of Facebook use and perceived self-esteem, among others: active Facebook use (Hanna et al., 2017), active hours on Facebook (Baturay and Toker, 2017), education as intended purpose for using Facebook (Eşkisu et al., 2017), frequency of Facebook use (Cudo et al., 2020a,b; Türkmen et al., 2022), information search on Facebook (Castillo de Mesa et al., 2020), inspection time of social updates on Facebook (Hussain et al., 2019), lying behavior on Facebook (Wright et al., 2018), mobile Facebook use (Schmuck et al., 2019), number of Facebook logins (Skues et al., 2012), perceived level of activity on Facebook (Michikyan et al., 2015), perceived level of awareness when using Facebook (Tazghini and Siedlecki, 2013), public communication with Facebook friends (Manago et al., 2012), reading on Facebook (Cramer et al., 2016), social interaction as intended purpose for using Facebook (Eşkisu et al., 2017), tolerance of diversity on Facebook (Castillo de Mesa et al., 2020), use and presence of Facebook in life (Castillo de Mesa et al., 2020), and use of Facebook for search for relations (Bergagna and Tartaglia, 2018). A summary of all effects of the sixty-six studies that examined the psychological effects of Facebook use on perceived self-esteem can be found in Table 2.

#### 3.1.6. Perceived life satisfaction

Forty-four studies were found that examined the psychological effects of Facebook use on perceived life satisfaction. Results varied widely, ranging from no effect to a strong effect. The 44 studies included 37 cross-sectional studies (37 surveys) and 7 longitudinal studies (4 panel studies, 2 longitudinal randomized experiments, and 1 longitudinal study with a time-series design).

Examples of negative effects on perceived life satisfaction at a low to moderate level include various Facebook activities such as looking at other’s photos/videos on Facebook (Vigil and Wu, 2015), tagging photos on Facebook (Vigil and Wu, 2015), or uploading photos on Facebook (Vigil and Wu, 2015). Compensatory Facebook Use (Goljović, 2017), Facebook addiction (Akın and Akın, 2015; Biolcati et al., 2018; Satici, 2019), Facebook intrusion (Błachnio et al., 2019), passive Facebook use (Frison and Eggermont, 2016b), passive following on Facebook (Wenninger et al., 2014), or time spent on Facebook (Vigil and Wu, 2015; Frison and Eggermont, 2016b; Stieger, 2019) were also negatively associated with perceived life satisfaction.

Positive effects on perceived life satisfaction were mainly due to active Facebook use (Choi, 2022), Facebook check-in intensity (Wang, 2013), and general Facebook use (Basilisco and Cha, 2015; Srivastava, 2015; Brailovskaia and Margraf, 2016). Facebook network size (Manago et al., 2012), number of Facebook friends (Nabi et al., 2013; Srivastava, 2015; Vigil and Wu, 2015; Lönnqvist and große Deters, 2016), number of Facebook hours per week (Cudo et al., 2020a,b), perceived social attention on Facebook (Adnan and Mavi, 2015), or perceived social connectedness from the use of Facebook (Grieve et al., 2013) also influenced perceived life satisfaction in positive ways. A 20-min reduction in daily Facebook time produced a steady increase in perceived life satisfaction scores over a three-month period (Brailovskaia et al., 2020a, 2020b). Furthermore, one study showed that increasing Facebook use over time is associated with lower perceived life satisfaction (Kross et al., 2013). This finding is consistent with another study that found perceived life satisfaction increased after a one-week absence from Facebook (Tromholt, 2016). In contrast to these results, Facebook users had significantly higher mean scores for perceived life satisfaction compared to non-Facebook users (Brailovskaia and Margraf, 2016).

No statistically significant effect was found between the following types of Facebook use and perceived life satisfaction, among others: commenting on Facebook (Wenninger et al., 2014), communication as motive for using Facebook (Adnan and Mavi, 2015), connection as motive for using Facebook (Rae and Lonborg, 2015), Facebook account length (Locatelli et al., 2012; Kang et al., 2013), friendship as motive for using Facebook (Rae and Lonborg, 2015), information as motive for using Facebook (Adnan and Mavi, 2015; Rae and Lonborg, 2015), information search on Facebook (Castillo de Mesa et al., 2020), level of interest in Facebook use (Kang et al., 2013), liking on Facebook (Wenninger et al., 2014), mobile Facebook use (Schmuck et al., 2019), perceived frequency of writing status updates on Facebook (Locatelli et al., 2012), private communication with Facebook friends (Manago et al., 2012), and use and presence of Facebook in life (Castillo de Mesa et al., 2020). A summary of all effects of the forty-four studies that examined the psychological effects of Facebook use on perceived life satisfaction can be found in Table 2.

#### 3.1.7. Perceived insomnia

Sixteen studies were found that examined the psychological effects of Facebook use on perceived insomnia. Results varied slightly, ranging from no effect to a small effect. The 16 studies included 15 cross-sectional studies (15 surveys) and 1 longitudinal study (1 panel study).

Facebook addiction was significantly positively associated with perceived poorer sleep quality (Wang et al., 2021; Ho, 2021a; Ho et al., 2021a), perceived insomnia (Koc and Gulyagci, 2013; Brailovskaia et al., 2019a), and perceived sleep disturbance (Ho, 2021b). Furthermore, research showed that problematic Facebook use was significantly positively correlated with perceived poorer sleep quality (Ho et al., 2021a). Indeed, daily Facebook use was significantly positively correlated with perceived insomnia over time (Brailovskaia et al., 2019a). Such findings are supported by other research, which found that Facebook intrusion was positively associated with perceived sleep problems (Przepiórka and Błachnio, 2020) and perceived insomnia (Błachnio et al., 2021). Additionally, one study showed that Facebook addiction was also significantly negatively associated with perceived sleep quality (Atroszko et al., 2018), and another study concluded that individuals with a Facebook addiction were at high risk of developing insomnia (Hanprathet et al., 2015).

No statistically significant effect was found between the following types of Facebook use and perceived insomnia, among others: academic motive for using Facebook (Koc and Gulyagci, 2013), daily Facebook use (Brailovskaia et al., 2019a), daily informational motive for using Facebook (Koc and Gulyagci, 2013), social motive for using Facebook (Koc and Gulyagci, 2013), and weekly time commitment on Facebook (Koc and Gulyagci, 2013). A summary of all effects of the sixteen studies that examined the psychological effects of Facebook use on perceived insomnia can be found in Table 2.

#### 3.1.8. Perceived stress

Twenty-seven studies were found that examined the psychological effects of Facebook use on perceived stress. Results varied widely, ranging from no effect to a strong effect. The 27 studies included 24 cross-sectional studies (24 surveys) and 3 longitudinal studies (3 panel studies).

Results show that perceived stress was primarily very strongly associated with Facebook addiction. For example, Brailovskaia et al. (2019a) found a very strong correlation between Facebook addiction and daily stress in both the U.S. and German samples. A strong positive correlation was also found in the study by Brailovskaia et al. (2019c). Moreover, Facebook addiction correlated with stress at low (Brailovskaia and Margraf, 2017; Atroszko et al., 2018, 2022; Eşkisu et al., 2020; Verseillié et al., 2021; Balcerowska et al., 2022) and medium (Brailovskaia et al., 2018b; Ho, 2021b) levels. Further positive effects on perceived stress at low and/or moderate levels include Facebook intensity (O’Sullivan and Hussain, 2017; Pal et al., 2018; Brailovskaia et al., 2019c; Nazzal et al., 2021), perceived emotional engagement with Facebook (Verseillié et al., 2021), perceived online social support received from other Facebook users (Brailovskaia et al., 2019a), perceived social comparison on Facebook (Flynn et al., 2018), problematic Facebook use (Nasser et al., 2019; Ho et al., 2021a), and risky and impulsive Facebook use (Flynn et al., 2018). However, one study found a significant negative correlation between the number of Facebook friends and perceived stress (Nabi et al., 2013), albeit at a low level.

No statistically significant effect was found between the following types of Facebook use and perceived stress, among others: Facebook account length (Bevan et al., 2014; Hussain et al., 2019), Facebook session length (Hussain et al., 2019), inspection time of Facebook updates (Hussain et al., 2019), inspection time of social updates on Facebook (Hussain et al., 2019), lying behavior on Facebook (Wright et al., 2018), and temporary break from Facebook use (O’Sullivan and Hussain, 2017). A summary of all effects of the twenty-seven studies that examined the psychological effects of Facebook use on perceived stress can be found in Table 2.

### 3.2. Physiological effects of Facebook Use

We found 15 empirical studies that examined physiological effects of Facebook use. The 15 studies included 7 experimental studies (47%), 6 longitudinal studies (40%), and 2 cross-sectional studies (13%). Our analysis revealed that Facebook use is associated with three major physiological effects, which we discuss in the following. We summarize the identified papers on the physiological effects of Facebook use with their effect type, based on results which are reported as statistically significant (negative [−], positive [+], no effect [∼] in Table 3). To reveal the scope, range, and nature of prior empirical research on how Facebook use is associated with these physiological effects, we considered the research context of the identified studies rather than just the effect direction. For example, we classified the studies by Campisi et al. (2012, 2017) as reporting negative effects because they found that increasing Facebook network size was positively associated with an increasing upper respiratory infections rate. Note that we also classified one paper as “descriptive [/]” (He et al., 2017).

**Table 3:** Studies on physiological effects of Facebook use.

Construct	Details		
Physiological Stress	Studies	Afifi et al. (2018) [−]; Campisi et al. (2012) [−]; Campisi et al. (2017) [−]; Cipresso et al. (2019) [−]; Moreno et al. (2014) [∼]; Morin-Major et al. (2016) [−][∼]; Rus and Tiemensma (2017) [−]; Rus and Tiemensma (2018) [+]; Vanman et al. (2018) [∼]	
	Descriptive Information	Total number of studies	9
		Number of studies reporting a negative effect	6
		Number of studies reporting a positive effect	1
		Number of studies reporting no effect	3
		Number of descriptive studies	0
	Negative Effects	Increased cognitive stress when looking at own Facebook profile (Cipresso et al., 2019), increased level of Facebook-induced anxiety or stress corresponds with a higher number of upper respiratory infections (Campisi et al., 2017), increased level of subjective and physiological stress when engaging with own Facebook profile after experiencing an acute social stressor (Rus and Tiemensma, 2017), increasing Facebook network diversity and feelings associated with being defriended on Facebook with increased incidence of upper respiratory infections (Campisi et al., 2012), increasing Facebook network size with an increase in cortisol awakening response (Morin-Major et al., 2016), increasing Facebook network size with an increasing upper respiratory infections rate (Campisi et al., 2012, 2017), increasing Facebook use with an increase in cortisol awakening response (Afifi et al., 2018), and increasing Facebook use with an increase in inflammation (Afifi et al., 2018)	
	Positive Effect	Decreased level of psychosocial stress when engaging with own Facebook profile before experiencing an acute social stressor (Rus and Tiemensma, 2018)	
	No Effects	Cortisol level and pulse changes during Facebook use (Moreno et al., 2014), cortisol level decline and temporary absence from Facebook (Vanman et al., 2018), cortisol systemic output and decline from supper time to bedtime (Morin-Major et al., 2016), Facebook peer-interactions and cortisol systemic output (Morin-Major et al., 2016), frequency of Facebook use and cortisol systemic output (Morin-Major et al., 2016), and self-presentation on Facebook and cortisol systemic output (Morin-Major et al., 2016)	
	Example	Increasing Facebook network size has been linked to the negative physiological effects of Facebook use associated with physiological stress (Campisi et al., 2012, 2017).	
Human Brain Alteration	Studies	He et al. (2017) [/]; He et al. (2018) [−][∼]; Montag et al. (2017) [−][∼]	
	Descriptive Information	Total number of studies	3
		Number of studies reporting a negative effect	2
		Number of studies reporting a positive effect	0
		Number of studies reporting no effect	2
		Number of descriptive studies	1
	Negative Effects	Duration of daily Facebook use and association with gray matter volume of left accumbens (Montag et al., 2017), excessive Facebook use with fractional anisotropy of the right corticospinal tract (He et al., 2018), excessive Facebook use with mean diffusivity in the splenium of corpus callosum (He et al., 2018), excessive Facebook use with mean diffusivity of the left forceps minor (He et al., 2018), excessive Facebook use with mean diffusivity of the left inferior longitudinal fasciculus (He et al., 2018), excessive Facebook use with mean diffusivity of the left superior longitudinal fasciculus (He et al., 2018), frequency of Facebook use and association with gray matter volume of left accumbens (Montag et al., 2017), and frequency of Facebook use and association with gray matter volume of right accumbens (Montag et al., 2017)	
	Positive Effects	N/A	
	No Effects	Duration of daily Facebook use and association with gray matter volume of left accumbens (Montag et al., 2017), excessive Facebook use with fractional anisotropy of the body of corpus callosum (He et al., 2018), excessive Facebook use with fractional anisotropy of the genu of corpus callosum (He et al., 2018), excessive Facebook use with fractional anisotropy of the splenium of corpus callosum (He et al., 2018), excessive Facebook use with mean diffusivity in the body of corpus callosum (He et al., 2018), and excessive Facebook use with mean diffusivity in the genu of corpus callosum (He et al., 2018)	
	Example	Excessive Facebook use has been linked to the negative physiological effects of Facebook use associated with human brain alteration (He et al., 2018).	
Affective Experience State	Studies	Cipresso et al. (2015) [−][+]; Mauri et al. (2011) [+]; Rauch et al. (2014) [−][+]	
	Descriptive Information	Total number of studies	3
		Number of studies reporting a negative effect	2
		Number of studies reporting a positive effect	3
		Number of studies reporting no effect	0
		Number of descriptive studies	0
	Negative Effects	Increased anxiety when navigating Facebook (Cipresso et al., 2015) and increased physiological arousal during a face-to-face encounter with prior Facebook exposure (Rauch et al., 2014)	
	Positive Effects	Increased emotional valence when navigating Facebook (Mauri et al., 2011; Cipresso et al., 2015), increased physiological arousal when navigating Facebook (Mauri et al., 2011; Cipresso et al., 2015), and increased sustained attention when navigating Facebook navigation (Cipresso et al., 2015)	
	No Effects	N/A	
	Example	Increased anxiety when navigating Facebook has been linked to the negative physiological effects of Facebook use associated with affective experience states (Cipresso et al., 2015).	

#### 3.2.1. Physiological stress

Nine studies examined the effects of Facebook use on physiological stress. Results varied widely, ranging from no effect to a strong effect. The 9 studies included 5 longitudinal studies (4 longitudinal studies with a time-series design and 1 longitudinal randomized experiment) and 4 experimental studies (3 experimental studies with an RCT design and 1 quasi-experiment).

The aim of the study by Afifi et al. (2018) was to determine the effects of technology and media use on stress and inflammation. At the beginning of the study, each participant completed a questionnaire and kept a diary of technology and media use, nighttime technology use, and hours of sleep from Monday to Friday. Saliva samples were used to determine cortisol and inflammation levels. Saliva samples were collected immediately after waking in the morning, 30 min after waking, at noon, and immediately before bedtime. Two main effects of Facebook use on stress and inflammation were found in the adolescents. With increasing Facebook use, cortisol awakening response and inflammation levels increased.

Campisi et al. (2012) investigated the association between Facebook use and upper respiratory infections (URI). Survey analysis revealed that most participants had difficulty completing their study assignments due to the high levels of stress they had experienced in the previous 3 months. The average number of infections during the 10-week period was 2.5 infections per person. The results also suggest that the Facebook network size (i.e., number of Facebook friends) had an impact on the frequency of URIs, and also on the average number of URIs per person. In addition, there was a significant relationship between the occurrence of URIs and the feeling of anger or sadness when someone ended their Facebook friendship. Facebook-induced stress had no significant effect on the frequency of URIs or on the average number of URIs per individual. Campisi et al. (2012) argued that chronic stress can affect the immune system. Users who are stressed by Facebook use may therefore have a weakened immune system.

In another study, Campisi et al. (2017) examined whether the interaction between Facebook use and stress can be explained by Facebook users’ behavior. To record the occurrence of URIs, participants had to keep a weekly diary for 10 weeks. Analysis of the data revealed a strong influence of social network size on the average number of URIs per person. Participants who experienced anxiety or stress due to Facebook use had a significantly higher number of URIs compared to individuals who did not experience Facebook-induced anxiety or stress. Also, there was a significant positive correlation between the number of Facebook logins per day and the number of URIs.

The study by Cipresso et al. (2019) sought to determine whether the psychological stress of navigating one’s own Facebook profile was higher, lower, or the same as navigating the profiles of other users. Physiological measurements were used to assess participants’ psychophysiological state. Participants were instructed to move freely on Facebook for 5 min. This allowed them, for example, to click on anything and go to any page within their own Facebook account. Eye-tracking data was collected to determine whether participants were viewing content that was related to themselves or to content that was related to others. Results showed that psychological stress increased significantly when viewing content that is related to oneself compared to viewing content that is related to others. Cipresso et al. (2019) reached this conclusion based on decreased heart rate variability, increased sympathetic component, and increased sympathovagal balance.

Moreno et al. (2014) investigated whether the biological response to stress is influenced by Facebook use and undertook a characterization of participants’ Facebook use during a stressful event. The biological response was measured using salivary cortisol samples and a radial pulse measurement. The cortisol level increased in the Facebook group, while it decreased in the control group. In the Facebook group, the pulse increased more compared to the control group and stabilized toward the end of the experimental session. However, there were no significant differences in either the Facebook group or the control group with respect to the change in cortisol level or pulse. The male participants in the Facebook group had above-average pulse values and showed increased biological signs of stress during a stressful event, which were predominantly attributed to the distracting use of Facebook.

The aim of the study by Morin-Major et al. (2016) was to examine the relationships between adolescents’ basal levels of diurnal cortisol and various Facebook behaviors, specifically frequency of use, self-expression, peer interaction, and network size. Cortisol levels were measured on two nonconsecutive weekdays over a three-week period. Significant correlations existed between Facebook network size and cortisol awakening response, systemic cortisol output, and perceived stress. In addition, frequency of Facebook use correlated with perceived stress, and perceived stress correlated with cortisol awakening response and systemic cortisol output. Sensitivity analyses were also conducted to examine which diurnal cortisol timeframe was most strongly associated with Facebook behavior. Morin-Major et al. (2016) found that Facebook network size was significantly positively associated with cortisol awakening response, which included changes from awakening to 30 min after. However, no associations were found between Facebook behavior and the decline in cortisol levels from supper time to bedtime.

Rus and Tiemensma (2017) investigated the influence of Facebook in terms of reactivity to an acute social stressor. They used both physiological (saliva samples, blood pressure, and heart rate) and psychosocial measures (Facebook use, mood, well-being, and subjective stress) to measure changes in physiological and subjective stress, as well as use behavior. As a result of the acute stressor (Trier Social Stress Test, TSST; Kirschbaum et al., 1993), participants experienced changes in both physiological and subjective stress. However, individuals who belonged to the Facebook user group surprisingly responded to the stressor with lower levels of physiological stress (systolic blood pressure) as well as lower levels of psychosocial stress. The same outcome was observed in the recovery phase. Based on the results, Rus and Tiemensma (2017) concluded that Facebook use prior to experiencing an acute stressor may have a buffering effect, particularly with respect to psychosocial stress.

In another study, Rus and Tiemensma (2018) examined how Facebook use affects recovery from stress (induced by the TSST; Kirschbaum et al., 1993). At the beginning of the study, participants completed a questionnaire about the intensity of Facebook use (measured with the Facebook Intensity Scale; Ellison et al., 2007). To examine the effect of Facebook use on a stress response, participants were then randomly assigned to either use their own Facebook account (experimental condition) or to use optional digital reading material for 20 min (control condition) before subsequently undergoing a TSST. To measure physiological markers of stress in response to the TSST, saliva samples were collected at baseline and at various time points during the study, blood pressure and heart rate were measured continuously, and psychosocial stress was assessed in the form of self-reports at various time points during the study. Upon completion of the TSST, all participants had 30 min of recovery as well as access to the digital reading material provided in the control condition. During the recovery phase, participants in both groups experienced similar changes in psychosocial stress. However, physiological recovery was inhibited in the Facebook group. This group had higher cortisol levels compared to the control group. Effects of Facebook use on blood pressure, heart rate, and psychosocial stress were not detected despite the elevated cortisol levels. Although individuals in the experimental group showed a sustained physiological stress response, participants in this group reported recovering as well as the subjects in the control group. Altogether, Rus and Tiemensma (2018) showed that Facebook use can delay or impair recovery after a stressor.

Vanman et al. (2018) determined whether a five-day Facebook break would reduce both stress and subjective well-being. Participants filled out surveys at the beginning of the study to assess stress and well-being. This was followed by taking the first saliva sample. Next, a program randomly assigned study participants to one of two conditions: One group was instructed to use Facebook as usual until the second session, while the other group was not allowed to use Facebook. At the beginning of the study, there was no difference between the cortisol levels of the two groups. However, later there was a decrease in cortisol levels in the group without Facebook. In contrast, cortisol levels in the Facebook group remained relatively unchanged. Thus, Vanman et al. (2018) showed that even a five-day Facebook break can lead to lower cortisol levels. However, the individuals who abstained from Facebook for 5 days reported lower levels of life satisfaction compared to the Facebook group.

#### 3.2.2. Human brain alteration

Three studies were found that examined the effects of Facebook use on human brain alteration. Results varied widely, ranging from no effect to a strong effect. The 3 studies included 2 cross-sectional studies (1 cross-sectional screening survey study and 1 case–control screening survey study) and 1 longitudinal study (1 longitudinal study with a time-series design).

The aim of the study by He et al. (2017) was to investigate the relationship between excessive social media use and gray matter volume in key neural systems. For this purpose, the behavioral pattern of social media use of the 50 study participants was determined by a Facebook-specific adaptation of the Compulsive Internet Use Instrument (Meerkerk et al., 2009; Turel et al., 2014), and participants were then categorized into a low or high behavior pattern of excessive social media use using a median split. The results of the region-of-interest analysis showed that in the case group (relatively high scores for excessive Facebook use compared to control group with relatively low scores), gray matter volume was decreased in both the bilateral amygdala and the right ventral striatum compared to the control group. There was a negative correlation between excessive Facebook use and the gray matter volume of the left amygdala, right amygdala, and right ventral striatum. No differences or correlations were found in prefrontal regions between the two groups.

The study by He et al. (2018) examined the association between excessive social media use and the impaired integrity of the white matter of the corpus callosum. After participants completed a questionnaire on demographics, data on Facebook use, and excessive Facebook use, as well as a structural magnetic resonance imaging (sMRI) scan was collected. Region-of-interest analysis revealed significant positive correlations between excessive Facebook use and mean diffusivity in both the body and the splenium of corpus callosum. However, the correlation with the mean diffusivity in the body of corpus callosum and excessive Facebook use was no longer significant after FDR correction. Also, fractional anisotropy of the right corticospinal tract and mean diffusivity of the left superior longitudinal fasciculus, inferior longitudinal fasciculus, and left forceps minor correlated positively with excessive Facebook use. Correlations between the mean diffusivity in the genu of corpus callosum and excessive Facebook as well as fractional anisotropy in the body, genu and splenium of corpus callosum and excessive Facebook use were not significant.

Montag et al. (2017) investigated the relationship between actual Facebook use and the nucleus accumbens. The nucleus accumbens, the major component of the ventral striatum, plays an important role in mediating emotion and motivation and modulating reward and pleasure processing, and also functions as an important limbic-motor interface (Cohen et al., 2009; Salgado and Kaplitt, 2015). It has also been linked to numerous neurological and psychiatric disorders, including depression, Parkinson’s disease, anxiety disorders, and substance abuse and dependence (Salgado and Kaplitt, 2015). Participants underwent sMRI at the beginning of the study and completed a questionnaire to determine addictive tendencies when using online social networks. Then, a self-developed application called “Menthal” was installed on the smartphone of all participating subjects to record user behavior on smartphones (for details of the application, please see Andone et al., 2016a,b). This application was used to record the duration of daily Facebook use and the frequency of daily Facebook app use over a five-week period. Significant negative correlations were found between both the duration of Facebook use and the gray matter volume of the left and right nucleus accumbens and between the frequency of Facebook use and the gray matter volume of the left and right nucleus accumbens. To control for brain volume, Montag et al. (2017) performed an additional calculation in which the ratio between the nucleus accumbens of the left/right hemisphere and the gray matter of each hemisphere was calculated. A significant relationship regarding Facebook use duration could only be found for the gray matter volume of left accumbens. The frequency of Facebook use correlated significantly with both the gray matter volume of left accumbens and the right accumbens. No significant correlation was found between the duration and frequency of Facebook use and the gray matter volumes of the left or right amygdala or hippocampus as control regions.

#### 3.2.3. Affective experience state

Three studies were found that examined the physiological effects of Facebook use on affective experience state. Results varied, ranging from a small effect to a strong effect. The 3 studies included 3 experimental studies (2 experimental studies with an RCT design and 1 quasi-experiment).

Cipresso et al. (2015) investigated users’ subjective experience of Facebook navigation via PC and via smartphone using physiological measurements. All participants underwent three conditions, namely relaxation, free navigation on Facebook, and stress (in the form of performing a Stroop task). Results show that Facebook was not perceived as disruptive, rather it was perceived as positive and activating. Facebook was found not to cause stress, instead eliciting positive emotional valence along with increased physiological arousal during Facebook navigation.

Mauri et al. (2011) examined whether Facebook use elicited a specific psychophysiological activation pattern. As an initial stimulus, participants were shown a series of panoramic images for relaxation. They were then allowed to move freely on Facebook for 3 min. This was followed by a stress phase, which included a Stroop task and a math task. The Facebook navigation scores showed different trends, except for the scores related to breathing and EEG beta waves. These were almost exactly between the values for relaxation and stress. Skin conductance values for Facebook navigation were very similar to the stress condition. Moreover, regarding the heart interbeat interval, the relaxation and Facebook conditions were almost identical. The lowest values for pupil dilation (less dilation is interpreted as less activation of the sympathetic part of the autonomic nervous system) and electromyography activity from Corrugator Supercilii were measured during Facebook navigation (note that Corrugator Supercilii muscle activity is considered a measure of emotional valence; it usually decreases in response to positive emotions and it increases in response to negative emotions; e.g., Neta et al., 2009). Thus, this study found that there was a significant difference between the Facebook experience and the relaxation and stress conditions for many indices of somatic activity, and that Facebook use produced a state characterized by positive emotion and high arousal.

The study by Rauch et al. (2014) examined the effects of Facebook exposure through a subsequent face-to-face situation with a stimulus person on physiological arousal levels. Approximately 1 week prior to the experimental session, participants were asked to complete a social anxiety survey. During the experimental session, skin conductance was used to measure physiological arousal levels while exposed to a person via Facebook, face-to-face, or both. Results showed that prior exposure to a Facebook stimulus led to increased physiological arousal during a face-to-face contact, especially in individuals with high social anxiety.

## 4. Review discussion

We contribute to research by providing an in-depth comprehension of the scope, range, and nature of the existing literature on the negative psychological and physiological effects of Facebook use. Specifically, we report evidence on how Facebook use is associated with eight identified psychological (perceived anxiety, perceived depression, perceived loneliness, perceived eating disorders, perceived self-esteem, perceived life satisfaction, perceived insomnia, and perceived stress) and three physiological (physiological stress, human brain alteration, and affective experience state) effects. Overall, the literature search process represents a systematic and methodologically rigorous process for examining the psychological and physiological effects of Facebook use.

The social network of Facebook is used for various reasons, such as communication (Aydın et al., 2013), entertainment (Ögel-Balaban and Altan, 2020), friendship (Rae and Lonborg, 2015), or social inclusion (Teppers et al., 2014). The main implication for research is that the results of this review suggest that the various psychological and physiological effects depend on the type of Facebook use. Facebook addiction, as a negative consequence of an excessive and uncontrolled Facebook use, is highly associated with the identified effects. For example, a significant positive association was found between Facebook addiction and perceived stress (Brailovskaia et al., 2019c). Negative psychological and physiological effects caused by excessive and uncontrolled Facebook use behavior may also develop over time. As evidence for this conclusion, we rely on a longitudinal study by Brailovskaia and Margraf (2017), who found a significant positive association between Facebook addiction and perceived anxiety, perceived depression, *and* perceived stress in a German student sample over a one-year period, although the extent of Facebook use did not change noticeably. The same study also revealed that the number of individuals with problematic Facebook use behavior can increase significantly within 1 year. However, research has also found approaches to counteract the negative effects. For example, a study by Brailovskaia et al. (2020b) found that reducing daily Facebook use even over a 14-day period can significantly reduce depressive symptoms while significantly increasing life satisfaction. This finding is supported by other studies that showed that a temporary absence from Facebook can significantly increase life satisfaction (Tromholt, 2016) and also reduce the cortisol level as indicator of physiological stress (Vanman et al., 2018). Given the potential risks of excessive and uncontrolled Facebook use, this review therefore provides a fundamental understanding of the psychological (see Table 2) and physiological (see Table 3) effects of Facebook use based on empirical research.

From a practical perspective, our paper highlights the importance of the knowledge on the negative psychological and physiological effects of Facebook use. We note, though, that the results are also temporary in nature, as research in this area will also face new challenges. One of these challenges, which has been increasingly observed in scientific research and practice in recent years, is the individual habit of constantly checking IT devices for new information to stay always up-to-date (Stangl and Riedl, 2023c). In this regard, mobile technologies (e.g., smartphone) are particularly problematic, as auditory and/or visual notifications (Tams et al., 2020) have the potential to contribute to the development of addictive behavioral tendencies (e.g., looking at the smartphone every few minutes for a new SNS notification; Sha et al., 2019). Here, insights into the appearance of different modalities of Facebook-induced notifications would also be valuable for interruption science, an interdisciplinary research field that systematically investigates the prevalent phenomenon of interruptions (Stangl and Riedl, 2023b, 2023e). However, research has shown that users turn on their smartphone screens 88 times a day, with SNSs accounting for the majority of the average 2.5 h of mobile phone usage (Markowetz, 2015). Such behavior may be considered as an additional excessive and uncontrolled Facebook use behavior, which Keller et al. (2021) characteristically refers to as “lack of control about one’s smartphone use” (p. 2). As an implication for practice, further research activities and findings on the negative psychological and physiological effects of Facebook use, including a focus on mobile technologies, are therefore particularly valuable, which in turn will lead to the discovery of additional SNS-relevant constructs.

### 4.1. Potentials for future research activities

Building on the research results of our scoping review, we derived five major potentials for future research activities.

**Potential 1: Additional Neuroscientific and Neurophysiological Studies** – As first potential for future research activities, we highlight the value of neuroscientific and neurophysiological studies to further investigate Facebook use behavior and the identified psychological and physiological effects of Facebook use. Indeed, to determine how and why certain psychological or physiological effects occur during Facebook use, neuroscience and neurophysiological tools and methods used in the interdisciplinary scientific field of NeuroIS can contribute to enhancing our understanding of human cognition, emotion, and behavior (Riedl et al., 2010, 2014, 2017; Dimoka et al., 2012; Riedl and Léger, 2016). For example, Triệu et al. (2021) used eye-tracking data and found that individuals with more social content on their Facebook newsfeed who spent a longer time viewing other Facebook postings and clicking more on other Facebook postings reported lower self-esteem than individuals who used Facebook less intensively. From a methodological perspective, however, NeuroIS studies usually combine data from neurophysiological measurements with self-report data to investigate underlying effects and users’ cognitive and affective processes in human-computer interaction in more detail (Loos et al., 2010; Riedl et al., 2010, 2014, 2017; Dimoka et al., 2012; Riedl and Léger, 2016). As an example, Morin-Major et al. (2016) examined Facebook use behavior by combining salivary cortisol samples as a physiological measure and self-reported data collected with validated questionnaires measuring psychological measures. Therefore, to better understand Facebook use and its underlying behavior, future research activities using neuroscientific and neurophysiological knowledge and tools seems promising to expand and systematically examine in more detail our understanding of the psychological and physiological effects of Facebook use and its consequences.

**Potential 2: Insights through Digital Phenotyping and Mobile Sensing Principles** – Digital phenotyping and mobile sensing refer to studying a person’s digital footprints as an extended phenotype of a person (Jain et al., 2015) providing insights into diverse psychological characteristics (Baumeister and Montag, 2023). In particular, people’s digital footprints on Facebook, which are produced in the course of creating and maintaining personal profiles, can provide revealing information about many psychologically relevant characteristics such as personality (Marengo and Montag, 2020), perhaps even into human neurobiology (Montag et al., 2021b) and further our understanding of molecular processes in the human brain (Montag and Quintana, 2023), with the latter giving way to digital biomarkers. While the term “digital biomarker” is currently poorly defined in the literature (Montag et al., 2021a), digital biomarkers have the potential to provide direct insights into underlying human neurobiology (Montag et al., 2021b), which is relevant given the increasing importance of the consumer-centric perspective in digital health (Agarwal et al., 2020). For example, it has been shown that Facebook language data can be used to predict and diagnose early stage of depression (Eichstaedt et al., 2018), a condition being critically linked to diverse brain processes (Fries et al., 2023). From a NeuroIS perspective, however, neurophysiological data, such as heart rate and heart rate variability as physiological indicators measuring autonomic nervous system activity, can additionally contribute to a deeper understanding for various measurement purposes, such as perceived anxiety or perceived stress (Stangl and Riedl, 2022b). Here, measures related to the brain and human body in general could also gain relevance in future empirical research on digital detoxing (Stangl and Riedl, 2023d), which is a strategy to counteract the negative effects of digital technology use; this topic has received significant attention in both scientific research and practice in the recent past (Mirbabaie et al., 2022). Digital detoxing involves temporary or complete disengagement from digital technologies (e.g., temporary abstinence from Facebook), along with strategies to reduce exposure to them (e.g., reduction in time spent on Facebook) (Hager et al., 2023; Stangl and Riedl, 2023a,d).Importantly, ongoing technological progress has also opened up many possibilities of mobile measurements for biomarker detection and monitoring (Baumeister and Montag, 2023), such as novel methods (e.g., smart clothing) for data collection of physiological indicators (Stangl and Riedl, 2022a). However, general quality criteria for measurement methods in psychometrics and psychophysiology (Riedl et al., 2014), such as reliability and validity of wearable devices (Stangl and Riedl, 2022c), along with ethical, legal, and societal implications (Dagum and Montag, 2019; Montag et al., 2020a) need to be carefully considered and assessed beforehand. Future research activities using digital biomarkers as part of a neuroscientific study design to establish associations between human neurobiology and the digital footprints generated by users’ interactions to explore negative psychological and physiological effects of Facebook use behavior, though, appear promising for advancing research in this area.

**Potential 3: Insights through Multimethod Research** – As a third potential for future research, we emphasize the possibilities of multimethod research. In fact, the results of our review show that most studies on Facebook use behavior are cross-sectional survey studies (80%), while only a small proportion of all studies are longitudinal (13%), experimental (5%), or studies with a multimethod research design (2%). An example of multimethod research is the study by Ozimek and Bierhoff (2020), who used an experimental study with an RCT design and two survey studies to investigate short-term and long-term effects of using Facebook for comparative social comparison on self-esteem and depressive tendencies. This research approach showed both correlational and experimental evidence of a mediating association between Facebook use and depressive tendencies via ability-related comparisons and lower self-esteem. Another conceivable approach is the use of neurophysiological measures, which can play an important role in research designs as complementary and supplementary measures to gain a deeper understanding of the cognitive and affective processes that occur when individuals interact with Facebook. This perspective is also supported by seminal contributions to the NeuroIS research agenda (e.g., Dimoka et al., 2012). Drawing upon the neuroscience and neurophysiological tools and methods used in NeuroIS, researchers have a variety of measurement approaches at their disposal to study human neurophysiology in the context of Facebook use behavior. The instruments and methods that are applicable in such a research context can be broadly divided into measurement of the central nervous system, measurement of the peripheral nervous system, and measurement of the hormone system (for an overview of neurophysiological tools with a discussion of the strengths and weaknesses of each measurement method per research setting, please see Riedl & Léger (2016, pp. 47-72); for a more detailed discussion of methods used in cognitive neuroscience, please see Senior et al. (2009). However, consistent with the finding of another recent descriptive literature review of neuroscience research on human-smartphone interactions and the digital footprints users leave in their interactions with SNSs (Montag et al., 2021b), neuroscience research tends to be a laggard as a research approach for examining Facebook use behavior. In fact, our review found only 14 studies (i.e., Mauri et al., 2011; Moreno et al., 2014; Rauch et al., 2014; Cipresso et al., 2015, 2019; Morin-Major et al., 2016; He et al., 2017, 2018; Montag et al., 2017; Rus and Tiemensma, 2017, 2018; Afifi et al., 2018; Vanman et al., 2018; Triệu et al., 2021) that applied neurophysiological measurements in their study. Therefore, research that considers neurophysiological measures as an adjunct in the context of multimethod research offers a promising future research activity to examine Facebook use behavior in a more detailed and systematic manner.

**Potential 4: Extension of Review Results** – The fourth potential for future research activities relates to the extension of our review results. In this review, we considered the empirical literature on the negative psychological and physiological effects of Facebook use published before and in April 2022. An extended analysis of empirical studies on other SNSs such as Instagram or Twitter, though, may lead to further insights into the negative psychological and physiological effects of SNSs. This is of particular relevance, because social media platforms differ in their designs/addictive potential and might attract also different user groups (Marengo et al., 2020; Rozgonjuk et al., 2021b): Statistics show that global audiences of SNSs differ by age and gender. For example, 9.3 percent of the Facebook audience was women aged 18 to 24 (Statista, 2022b), with the Instagram audience in that demographic accounting for 13.4% (Statista, 2022c). Extending our review methodology with a focus on other SNSs may reveal additional negative SNS-related constructs, providing a bird’s eye view of negative psychological and physiological effects of SNSs. Another conceivable approach is to replicate our review methodology in the future. As research on Facebook use behavior continues to encounter new aspects over time, even the negative psychological and physiological constructs we identified are to some extent transitory. However, future desktop research that either extends our research findings to other SNSs using our research methodology or replicates our original review methodology may uncover additional SNS-relevant constructs to the negative psychological and physiological effects we identified. Overall, the opportunities highlighted to extend our review findings are another promising activity for future research.

**Potential 5: Considering the Data Business Model** – Much research in the past has not focused on the actual culprit impacting in negative ways upon human behavior and society including development of addictive behaviors when interacting with social media platforms such as Facebook (Montag and Hegelich, 2020): The current prevailing model to pay for use allowance of a social media service foresees that users pay with their data, which in turn is used for microtargeting. This data business model, also named surveillance capitalism (Zuboff, 2015), led to the creation of highly immersive platforms which have been designed over many years via AB-testing (Montag et al., 2019). Understanding why humans act as they do on the social media platforms needs to take into account the design elements in-built on these platforms (Sindermann et al., 2022). This is often very difficult at the moment, as APIs are often closed and so social media remains a black box (Montag et al., 2021a). Studying digital footprints of online users (see also Potential 2) when they are interacting with the platform by also using ecological momentary assessment reports will be of tremendous importance to understand the effects of social media use on well-being and other psychological variables. A meta-analysis showed that assessment of technology use via self-report and objective recordings can differ (Parry et al., 2021). For further complexities in this research area see also the work by Kross et al. (2021) and Montag et al. (2021d). Finally, we mention that Potential 5 - as outlined in this section - will be also of high relevance to understand what healthier social media environments might look like (Dhawan et al., 2022).

### 4.2. Mitigation of validity concerns of research results

The evaluation of the planning process is an essential step in assessing the validity of a research result (Henderson and Sifonis, 1988; Straub, 1989). To validate our scoping review methodology as a data collection method to identify the negative psychological and physiological effects of Facebook use based on the current state of scientific research, we slightly modified the instrumental validity types of Becker et al. (2013) to evaluate potential validity threats of our literature search process. This allowed us to identify four major validity concerns, which we were, however, able to mitigate accordingly in relation to our review and its methodology.

1. **Descriptive Validity:** This validity type indicates the extent to which observations accurately reflect the phenomenon of interest. To mitigate this threat, we consider our applied literature search process to data collection to be as comprehensive as possible. It also enables us to continuously renew data collection. The literature base identified in this way is listed in the Supplementary material to objectify the process of data collection.
2. **Theoretical Validity:** This validity type indicates the extent to which the true scope of a phenomenon of interest has been captured. To mitigate this threat, we carefully designed the search string by systematically combining Facebook with general psychological and physiological as well as field-specific search terms to find empirical studies that addressed the negative effects of Facebook use on a psychological and physiological level, thereby capturing the topic of this paper in its entirety. Also, the identified papers were then analyzed collaboratively by the author team to avoid bias in data extraction and classification.
3. **Interpretive Validity:** This validity type indicates the extent to which the conclusions relate precisely to a phenomenon of interest. To mitigate this threat, we relied on and drew conclusions from data obtained from our literature search. The data obtained in this way is listed in the Supplementary material to objectify the process of data analysis.
4. **Repeatability:** This validity type indicates the extent to which the data of the research process are accurate and consistent when performed repeatedly. To mitigate this threat, we described the research process in detail. We have also transparently presented all the data we received during the literature search process, such as an overview of the identified studies by construct (i.e., identified psychological and physiological effects), including time scale with research design, participants with country, sample size with female share, age, Facebook use measure(s), and strength of associations between Facebook use and its effects.

## 5. Concluding statement

The goal of this scoping review was to examine the scope, range, and nature of prior research on the negative psychological and physiological effects of Facebook use. Our systematic and methodologically rigorous literature search process allowed us to identify eight psychological effects (perceived anxiety, perceived depression, perceived loneliness, perceived eating disorders, perceived self-esteem, perceived life satisfaction, perceived insomnia, and perceived stress) and three physiological effects (physiological stress, human brain alteration, and affective experience state) of Facebook use. Overall, this review lays a valuable foundation for future research activities, as it also captures characteristics of prior empirical research by construct, including research design, sample, age, measures, and strength of associations between Facebook use and its effects for better understanding Facebook use from psychological and physiological perspectives.

Consistent with the findings of a recent article on the influence of SNS use on well-being (Verduyn et al., 2022), our review revealed that Facebook use may be beneficial to some extent on a psychological or physiological level. However, the (over-)use of Facebook also poses a myriad of detrimental and significant risks, both psychologically (see Table 2) and physiologically (see Table 3). It is therefore crucial to study Facebook use behavior in a more detailed and systematic manner, as prior empirical studies have shown that excessive and uncontrolled use behavior can lead to the development of problematic Facebook use with various negative psychological and physiological effects. To this end, we have described potential avenues for future research. Importantly, we anticipate that future research may also identify additional SNS-related constructs and user characteristics (e.g., personality) that moderate these effects. Future research should also consider experimental designs with neurophysiological measurements as complements to self-report and behavioral measures to draw more definitive conclusions about the effects (see Potential 1 and Potential 3). Moreover, future studies must not ignore potential changes in Facebook’s business model, because such changes may have significant effects on addictive behaviors that result from interaction with the specific features of the Facebook app (see Potential 5). Also, technological progress may increasingly allow longitudinal studies to discover and establish associations between human neurobiology and digital footprints generated by user interactions to examine and even detect early negative psychological and physiological effects of Facebook use behavior in a consumer-centric perspective of digital health (see Potential 2). Another promising activity for future research is to extend our findings to other SNSs (e.g., Instagram, Snapchat, or Twitter), which would provide a bird’s eye view of negative psychological and physiological effects that could also lead to the discovery of additional SNS-related constructs (see Potential 4). Thus, it will be interesting to see how scientific research on the psychological and physiological effects of Facebook use will continue to develop.

## Author contributions

RR was responsible for funding acquisition and conceptualized the study. FS and RK reviewed the literature under supervision of RR and CM. All authors wrote the manuscript together, and thus contributed to the manuscript. All authors contributed to the article and approved the submitted version.

## Funding

RR’s research is funded by the Austrian Science Fund (FWF) as part of the project “Technostress in Organizations” (project number: P 30865) and by the Austrian Research Promotion Agency (FFG) as part of the project “Interruption” at the University of Applied Sciences Upper Austria.

## Conflict of interest

The authors declare that the research was conducted in the absence of any commercial or financial relationships that could be construed as a potential conflict of interest.

## Publisher’s note

All claims expressed in this article are solely those of the authors and do not necessarily represent those of their affiliated organizations, or those of the publisher, the editors and the reviewers. Any product that may be evaluated in this article, or claim that may be made by its manufacturer, is not guaranteed or endorsed by the publisher.
