# Supplementary material for: Negative psychological and physiological effects of social networking site use: The example of Facebook
Source: Front Psychol. 2023 Aug 3;14:1141663. doi: 10.3389/fpsyg.2023.1141663 (PMC10435997; doi:10.3389/fpsyg.2023.1141663)
Supplement: Supplementary file 2 [file Data_Sheet_1.docx]

Supplementary Material

Negative Psychological and Physiological Effects of Social Networking Site Use: The Example of Facebook

Fabian J. Stangl, René Riedl*, Roman Kiemeswenger and Christian Montag

*** Correspondence:** René Riedl: Rene.Riedl@fh-steyr.at

# Appendix: Literature on Negative Psychological and Physiological Effects of Facebook Use

This appendix presents the literature base of our scoping review. The keywords used were terms that reflect the negative psychological and physiological effects of Facebook use. The literature search was conducted in two waves and included the literature databases ACM, IEEE Xplore, Science Direct, Scopus, and Web of Science. Our literature search yielded 217 papers on the psychological effects of Facebook use and 15 papers on the physiological effects of Facebook use (see **Table A1**) published before and in April 2022.

Table A1. Overview of Literature on Negative Psychological and Physiological Effects of Facebook Use

| **Negative Effect Type** | **References** |
| --- | --- |
| Psychological Effect | Adnan & Mavi (2015); Ahamed et al. (2021); Ahmed (2018); Akın & Akın (2015); Alfasi (2019); Atroszko et al. (2018, 2022); Aung & Tin (2020); Awobamise et al. (2022); Aydın et al. (2013); Bais & Reyes (2020); Baker & Oswald (2010); Balcerowska et al. (2022); Basilisco & Cha (2015); Baturay & Toker (2017); Bendayan & Blanca Mena (2019); Bergagna & Tartaglia (2018); Bevan et al. (2014); Biolcati et al. (2018); Błachnio & Przepiórka (2018, 2019); Błachnio et al. (2019); Błachnio, Przepiórka, Boruch, et al. (2016); Błachnio et al. (2021); Błachnio, Przepiórka, & Pantic (2016); Błachnio, Przepiórka, & Rudnicka (2016); Błachnio et al. (2018); Brailovskaia & Margraf (2016, 2017, 2019); Brailovskaia, Margraf, et al. (2019); Brailovskaia, Rohmann, et al. (2020); Brailovskaia, Rohmann, Bierhoff, Margraf, et al. (2019); Brailovskaia, Rohmann, Bierhoff, Schillack, et al. (2019); Brailovskaia, Ströse, et al. (2020); Brailovskaia, Teismann, et al. (2018); Brailovskaia, Velten, et al. (2019); Brown et al. (2021); Çakıcı et al. (2020); Castillo de Mesa et al. (2020); Chabrol et al. (2017); Chavez & Chavez Jr. (2017); W. Chen & Lee (2013); Y. Chen & Bello (2017); Choi (2022); Chow & Wan (2017); Cingel & Olsen (2018); Clayton et al. (2013); Cramer et al. (2016); Cudo, Szewczyk, et al. (2020); Cudo, Wojtasiński, et al. (2020); Cury et al. (2022); da Veiga et al. (2019); Damota (2019); Datu et al. (2012); Davidson & Farquhar (2014); Dempsey et al. (2019); Dibb & Foster (2021); Errasti et al. (2017); Eşkisu et al. (2020; 2017); Farahani et al. (2011); Faranda & Roberts (2019); Faraon & Kaipainen (2014); Flynn et al. (2018); Foroughi et al. (2019); Francis (2022); Frison et al. (2019); Frison & Eggermont (2015, 2016a, 2016b, 2020); Gerson et al. (2016); Giagkou et al. (2018); Giota & Kleftaras (2013); Goljović (2017); Gonzales & Hancock (2011); González-Nuevo et al. (2021); Grieve et al. (2013); große Deters & Mehl (2013); Hanna et al. (2017); Hanprathet et al. (2015); Ho (2021a, 2021b); Ho, Huynh, et al. (2021); Ho, Nguyen, et al. (2021); Hong et al. (2014); Hosen et al. (2021); Hu et al. (2017); Hummel & Smith (2015); Hussain et al. (2019); Iovu et al. (2020); K. Jang et al. (2016); Jenkins-Guarnieri et al. (2012); Jeri-Yabar et al. (2019); Jha et al. (2016); Jin (2013); Kalpidou et al. (2011); Kanat-Maymon et al. (2018); Kang et al. (2013); Karakose et al. (2016); Khalil et al. (2022); Khattak et al. (2017); E. S. Kim et al. (2020); Koc & Gulyagci (2013); Kross et al. (2013); Kulkarni & Deshpande (2019); Kumar et al. (2019); Labrague (2014); Lee-Won et al. (2015); J.-E. R. Lee et al. (2012); S. Lee (2020); S. Y. Lee (2014); Lemieux et al. (2013) Lim & Yang (2019); Locatelli et al. (2012); Longua Peterson et al. (2017); Lönnqvist & große Deters (2016); Lou et al. (2012); Louragli et al. (2019); Luqman et al. (2017); Mabe et al. (2014); Maglunog & Dy (2019); Malik & Khan (2015); Manago et al. (2012); Mannino et al. (2021); Marder et al. (2016); Marengo et al. (2021); Masciantonio et al. (2021); McCloskey et al. (2015); McCord et al. (2014); Metzler & Scheithauer (2017); Michikyan et al. (2015); Nabi et al. (2013); Nasser et al. (2019); Nazzal et al. (2021); Nisar et al. (2019); Nizami et al. (2017); Norman et al. (2017); O’Sullivan & Hussain (2017); Ögel-Balaban & Altan (2020); Omar & Subramanian (2013); Omolayo et al. (2013); Ozimek & Bierhoff (2020); Ozimek et al. (2021); Pal et al. (2018); S. Park et al. (2013); S. Y. Park & Baek (2018); Phu & Gow (2019); Primi et al. (2021); Przepiórka & Błachnio (2020); Przepiórka et al. (2021); Puccio et al. (2016); Rachubińska et al. (2021); Rae & Lonborg (2015); Rahman & Zakaria (2021); Rajesh & Rangaiah (2020); Rosen, Whaling, Rab, et al. (2013); Rosenthal et al. (2016); Ryan & Xenos (2011); Saleem et al. (2016); Satici (2019); Satici & Uysal (2015); Scherr & Brunet (2017); Scherr et al. (2019); Schmuck et al. (2019); Sehar et al. (2022); Seran et al. (2020); Shakya & Christakis (2017); Shaw et al. (2015); Shettar et al. (2017); Sillence et al. (2021); Simoncic et al. (2014); Skues et al. (2012); A. R. Smith et al. (2013); T. Smith & Short (2022); Soraci et al. (2020); Sotero et al. (2019); Srivastava (2015); Stănculescu & Griffiths (2021); Steers et al. (2014); Sternberg et al. (2020; 2018); Stieger (2019); Tandoc Jr. et al. (2015); Tandoc Jr. & Goh (2021); Tazghini & Siedlecki (2013); Teo et al. (2019); Teppers et al. (2014); Tobin & Graham (2020); Tosun & Kaşdarma (2020); Triệu et al. (2021); Tromholt (2016); Türkmen et al. (2022); Uram & Skalski (2022); Uttravanich & Blauw (2018); Valenzuela et al. (2009); Vannucci et al. (2019); Verseillié et al. (2021); Vigil & Wu (2015); Vogel et al. (2015; 2014); Walburg et al. (2016); Walker et al. (2015); K. Wang et al. (2018); S. S. Wang (2013); T.-J. Wang et al. (2021); Wenninger et al. (2014); Whitman & Gottdiener (2016); Wolniczak et al. (2013); E. J. Wright et al. (2018); K. B. Wright et al. (2013); Xie & Karan (2019); S. Ye et al. (2021); Yeshua-Katz & Zilberstein (2021); Zaffar et al. (2015); Zhang (2017) |
| Psychological Effect (*continued*) |  |
| Physiological Effect | Afifi et al. (2018); Campisi et al. (2012, 2017); Cipresso et al. (2019, 2015); He et al. (2018; 2017); Mauri et al. (2011); Montag et al. (2017); Moreno et al. (2014); Morin-Major et al. (2016); Rauch et al. (2014); Rus & Tiemensma (2017, 2018); Vanman et al. (2018) |

# References

Adnan, H. M., & Mavi, S. R. (2015). Facebook satisfaction, life satisfaction: Malaysian undergraduate experience. *Malaysian Journal of Communication*, *31*(2), 649–671.

Afifi, T. D., Zamanzadeh, N., Harrison, K., & Acevedo Callejas, M. L. (2018). WIRED: The impact of media and technology use on stress (cortisol) and inflammation (interleukin IL-6) in fast paced families. *Computers in Human Behavior*, *81*, 265–273. https://doi.org/10.1016/j.chb.2017.12.010

Ahamed, A. F. M. J., Limbu, Y. B., & Mamun, M. Al. (2021). Facebook usage intensity and compulsive buying tendency: The mediating role of envy, self-esteem, and self-promotion and the moderating role of depression. *International Journal of Electronic Marketing and Retailing*, *12*(1), 69–88. https://doi.org/10.1504/IJEMR.2021.112255

Ahmed, O. (2018). Relationship between loneliness and mental health among first-year undergraduate students: Mediating role of timeline browsing and chatting on Facebook. *International Journal of Contemporary Education*, *1*(2), 86–94. https://doi.org/10.11114/ijce.v1i2.3625

Akın, A., & Akın, U. (2015). The mediating role of social safeness on the relationship between Facebook® use and life satisfaction. *Psychological Reports*, *117*(2), 341–353. https://doi.org/10.2466/18.07.PR0.117c20z9

Alfasi, Y. (2019). The grass is always greener on my Friends’ profiles: The effect of Facebook social comparison on state self-esteem and depression. *Personality and Individual Differences*, *147*, 111–117. https://doi.org/10.1016/j.paid.2019.04.032

Atroszko, P. A., Balcerowska, J. M., Bereznowski, P., Biernatowska, A., Pallesen, S., & Schou Andreassen, C. (2018). Facebook addiction among Polish undergraduate students: Validity of measurement and relationship with personality and well-being. *Computers in Human Behavior*, *85*, 329–338. https://doi.org/10.1016/j.chb.2018.04.001

Atroszko, P. A., El Abiddine, F. Z., Malik, S., Mamun, M. A., Vally, Z., & Czerwiński, S. K. (2022). Lack of measurement invariance in a widely used Facebook addiction scale may thwart progress in research on social-network-use disorder: A cross-cultural study. *Computers in Human Behavior*, *128*, 107132. https://doi.org/10.1016/j.chb.2021.107132

Aung, E. E. S., & Tin, O. (2020). Facebook addiction and loneliness of university students from Sagaing District. *Journal of the Myanmar Academy of Arts and Science*, *18*(9B), 353–367.

Awobamise, A., Jarrar, Y., & Nweke, G. E. (2022). Social communication apprehension, self-esteem and Facebook addiction among university students in Uganda. *Contemporary Educational Technology*, *14*(2), ep354. https://doi.org/10.30935/cedtech/11542

Aydın, G. S., Muyan, M., & Demir, A. (2013). The investigation of Facebook usage purposes and shyness, loneliness. *Procedia - Social and Behavioral Sciences*, *93*, 737–741. https://doi.org/10.1016/j.sbspro.2013.09.272

Bais, D. M., & Reyes, M. E. S. (2020). Psychological predictors of Facebook addiction tendencies among Filipino millennials in Metro Manila. *IAFOR Journal of Psychology & the Behavioral Sciences*, *5*(2), 37–56. https://doi.org/10.22492/ijpbs.5.2.03

Baker, L. R., & Oswald, D. L. (2010). Shyness and online social networking services. *Journal of Social and Personal Relationships*, *27*(7), 873–889. https://doi.org/10.1177/0265407510375261

Balcerowska, J. M., Bereznowski, P., Biernatowska, A., Atroszko, P. A., Pallesen, S., & Andreassen, C. S. (2022). Is it meaningful to distinguish between Facebook addiction and social networking sites addiction? Psychometric analysis of Facebook addiction and social networking sites addiction scales. *Current Psychology*, *41*(2), 949–962. https://doi.org/10.1007/s12144-020-00625-3

Basilisco, R., & Cha, K. J. (2015). Uses and gratification motivation for using Facebook and the impact of Facebook usage on social capital and life satisfaction among Filipino users. *International Journal of Software Engineering and Its Applications*, *9*(4), 181–194.

Baturay, M. H., & Toker, S. (2017). Self-esteem shapes the impact of GPA and general health on Facebook addiction: A mediation analysis. *Social Science Computer Review*, *35*(5), 555–575. https://doi.org/10.1177/0894439316656606

Bendayan, R., & Blanca Mena, M. J. (2019). Spanish version of the Facebook Intrusion Questionnaire. *Psicothema*, *31*(2), 204–209. https://doi.org/10.7334/psicothema2018.301

Bergagna, E., & Tartaglia, S. (2018). Self-esteem, social comparison, and Facebook use. *Europe’s Journal of Psychology*, *14*(4), 831–845. https://doi.org/10.5964/ejop.v14i4.1592

Bevan, J. L., Gomez, R., & Sparks, L. (2014). Disclosures about important life events on Facebook: Relationships with stress and quality of life. *Computers in Human Behavior*, *39*, 246–253. https://doi.org/10.1016/j.chb.2014.07.021

Biolcati, R., Mancini, G., Pupi, V., & Mugheddu, V. (2018). Facebook addiction: Onset predictors. *Journal of Clinical Medicine*, *7*(6), 118. https://doi.org/10.3390/jcm7060118

Błachnio, A., & Przepiórka, A. (2018). Facebook intrusion, fear of missing out, narcissism, and life satisfaction: A cross-sectional study. *Psychiatry Research*, *259*, 514–519. https://doi.org/10.1016/j.psychres.2017.11.012

Błachnio, A., & Przepiórka, A. (2019). Be aware! If you start using Facebook problematically you will feel lonely: Phubbing, loneliness, self-esteem, and Facebook intrusion. A cross-sectional study. *Social Science Computer Review*, *37*(2), 270–278. https://doi.org/10.1177/0894439318754490

Błachnio, A., Przepiórka, A., Benvenuti, M., Mazzoni, E., & Seidman, G. (2019). Relations between Facebook intrusion, Internet addiction, life satisfaction, and self-esteem: A study in Italy and the USA. *International Journal of Mental Health and Addiction*, *17*(4), 793–805. https://doi.org/10.1007/s11469-018-0038-y

Błachnio, A., Przepiórka, A., Boruch, W., & Bałakier, E. (2016). Self-presentation styles, privacy, and loneliness as predictors of Facebook use in young people. *Personality and Individual Differences*, *94*, 26–31. https://doi.org/10.1016/j.paid.2015.12.051

Błachnio, A., Przepiórka, A., & Cudo, A. (2021). The relations between Facebook intrusion, emotional functioning, and health problems. *Current Psychology*. https://doi.org/10.1007/s12144-021-01374-7

Błachnio, A., Przepiórka, A., & Pantic, I. (2016). Association between Facebook addiction, self-esteem and life satisfaction: A cross-sectional study. *Computers in Human Behavior*, *55*, 701–705. https://doi.org/10.1016/j.chb.2015.10.026

Błachnio, A., Przepiórka, A., & Rudnicka, P. (2016). Narcissism and self-esteem as predictors of dimensions of Facebook use. *Personality and Individual Differences*, *90*, 296–301. https://doi.org/10.1016/j.paid.2015.11.018

Błachnio, A., Przepiórka, A., Wołońciej, M., Mahmoud, A. B., Holdoš, J., & Yafi, E. (2018). Loneliness, friendship, and Facebook intrusion. A study in Poland, Slovakia, Syria, Malaysia, and Ecuador. *Studia Psychologica*, *60*(3), 183–194. https://doi.org/10.21909/sp.2018.03.761

Brailovskaia, J., & Margraf, J. (2016). Comparing Facebook users and Facebook non-users: Relationship between personality traits and mental health variables – an exploratory study. *PLOS ONE*, *11*(12), e0166999. https://doi.org/10.1371/journal.pone.0166999

Brailovskaia, J., & Margraf, J. (2017). Facebook Addiction Disorder (FAD) among German students—A longitudinal approach. *PLOS ONE*, *12*(12), e0189719. https://doi.org/10.1371/journal.pone.0189719

Brailovskaia, J., & Margraf, J. (2019). I present myself and have a lot of Facebook-friends – Am I a happy narcissist!? *Personality and Individual Differences*, *148*, 11–16. https://doi.org/10.1016/j.paid.2019.05.022

Brailovskaia, J., Margraf, J., Schillack, H., & Köllner, V. (2019). Comparing mental health of Facebook users and Facebook non-users in an inpatient sample in Germany. *Journal of Affective Disorders*, *259*, 376–381. https://doi.org/10.1016/j.jad.2019.08.078

Brailovskaia, J., Rohmann, E., Bierhoff, H.-W., & Margraf, J. (2020). The anxious addictive narcissist: The relationship between grandiose and vulnerable narcissism, anxiety symptoms and Facebook addiction. *PLOS ONE*, *15*(11), e0241632. https://doi.org/10.1371/journal.pone.0241632

Brailovskaia, J., Rohmann, E., Bierhoff, H.-W., Margraf, J., & Köllner, V. (2019). Relationships between addictive Facebook use, depressiveness, insomnia, and positive mental health in an inpatient sample: A German longitudinal study. *Journal of Behavioral Addictions*, *8*(4), 703–713. https://doi.org/10.1556/2006.8.2019.63

Brailovskaia, J., Rohmann, E., Bierhoff, H.-W., Schillack, H., & Margraf, J. (2019). The relationship between daily stress, social support and Facebook Addiction Disorder. *Psychiatry Research*, *276*, 167–174. https://doi.org/10.1016/j.psychres.2019.05.014

Brailovskaia, J., Ströse, F., Schillack, H., & Margraf, J. (2020). Less Facebook use – More well-being and a healthier lifestyle? An experimental intervention study. *Computers in Human Behavior*, *108*, 106332. https://doi.org/10.1016/j.chb.2020.106332

Brailovskaia, J., Teismann, T., & Margraf, J. (2018). Physical activity mediates the association between daily stress and Facebook Addiction Disorder (FAD) – A longitudinal approach among German students. *Computers in Human Behavior*, *86*, 199–204. https://doi.org/10.1016/j.chb.2018.04.045

Brailovskaia, J., Velten, J., & Margraf, J. (2019). Relationship between daily stress, depression symptoms, and Facebook addiction disorder in Germany and in the United States. *Cyberpsychology, Behavior, and Social Networking*, *22*(9), 610–614. https://doi.org/10.1089/cyber.2019.0165

Brown, R. M., Roberts, S. G. B., & Pollet, T. V. (2021). Loneliness is negatively related to Facebook network size, but not related to Facebook network structure. *Cyberpsychology: Journal of Psychosocial Research on Cyberspace*, *15*(2). https://doi.org/10.5817/CP2021-2-6

Çakıcı, M., Babayiğit, A., Karaaziz, M., & Cumhur, Ö. (2020). The prevalence and risk factors of Facebook addiction: Does Facebook addiction is related with depression and PTSD? *Anatolian Journal of Psychiatry*, *21*(3), 245–252. https://doi.org/10.5455/apd.2020101

Campisi, J., Bynog, P., McGehee, H., Oakland, J. C., Quirk, S., Taga, C., & Taylor, M. (2012). Facebook, stress, and incidence of upper respiratory infection in undergraduate college students. *Cyberpsychology, Behavior, and Social Networking*, *15*(12), 675–681. https://doi.org/10.1089/cyber.2012.0156

Campisi, J., May, J., Burch, K., Larson, K., Doscher, J., Doherty, S., … Gahan, A. (2017). Anxiety-inducing Facebook behavior is associated with higher rates of upper respiratory infection in college-aged users. *Computers in Human Behavior*, *76*, 211–217. https://doi.org/10.1016/j.chb.2017.07.022

Castillo de Mesa, J., Gómez-Jacinto, L., López Peláez, A., & Erro-Garcés, A. (2020). Social networking sites and youth transition: The use of Facebook and personal well-being of social work young graduates. *Frontiers in Psychology*, *11*. https://doi.org/10.3389/fpsyg.2020.00230

Chabrol, H., Laconi, S., Delfour, M., & Moreau, A. (2017). Contributions of psychopathological and interpersonal variables to problematic Facebook use in adolescents and young adults. *International Journal of High Risk Behaviors and Addiction*, *6*(1), e32773. https://doi.org/10.5812/ijhrba.32773

Chavez, G. B., & Chavez Jr., F. C. (2017). Relationship between Facebook addiction and loneliness of Filipino high school students. *Liceo Journal of Higher Education Research*, *13*(1), 51–60. https://doi.org/10.7828/ljher.v13i1.1008

Chen, W., & Lee, K.-H. (2013). Sharing, liking, commenting, and distressed? The pathway between Facebook interaction and psychological distress. *Cyberpsychology, Behavior, and Social Networking*, *16*(10), 728–734. https://doi.org/10.1089/cyber.2012.0272

Chen, Y., & Bello, R. S. (2017). Does receiving or providing social support on Facebook influence life satisfaction? Stress as mediator and self-esteem as moderator. *International Journal of Communication*, *11*, 2926–2939.

Choi, J. (2022). Do Facebook and Instagram differ in their influence on life satisfaction? A study of college men and women in South Korea. *Cyberpsychology: Journal of Psychosocial Research on Cyberspace*, *16*(1). https://doi.org/10.5817/CP2022-1-2

Chow, T. S., & Wan, H. Y. (2017). Is there any ‘Facebook Depression’? Exploring the moderating roles of neuroticism, Facebook social comparison and envy. *Personality and Individual Differences*, *119*, 277–282. https://doi.org/10.1016/j.paid.2017.07.032

Cingel, D. P., & Olsen, M. K. (2018). Getting over the hump: Examining curvilinear relationships between adolescent self-esteem and Facebook use. *Journal of Broadcasting & Electronic Media*, *62*(2), 215–231. https://doi.org/10.1080/08838151.2018.1451860

Cipresso, P., Mauri, M., Semonella, M., Tuena, C., Balgera, A., Villamira, M., & Riva, G. (2019). Looking at one’s self through Facebook increases mental stress: A computational psychometric analysis by using eye-tracking and psychophysiology. *Cyberpsychology, Behavior, and Social Networking*, *22*(5), 307–314. https://doi.org/10.1089/cyber.2018.0602

Cipresso, P., Serino, S., Gaggioli, A., Albani, G., Mauro, A., & Riva, G. (2015). Psychometric modeling of the pervasive use of Facebook through psychophysiological measures: Stress or optimal experience? *Computers in Human Behavior*, *49*, 576–587. https://doi.org/10.1016/j.chb.2015.03.068

Clayton, R. B., Osborne, R. E., Miller, B. K., & Oberle, C. D. (2013). Loneliness, anxiousness, and substance use as predictors of Facebook use. *Computers in Human Behavior*, *29*(3), 687–693. https://doi.org/10.1016/j.chb.2012.12.002

Cramer, E. M., Song, H., & Drent, A. M. (2016). Social comparison on Facebook: Motivation, affective consequences, self-esteem, and Facebook fatigue. *Computers in Human Behavior*, *64*, 739–746. https://doi.org/10.1016/j.chb.2016.07.049

Cudo, A., Szewczyk, M., Błachnio, A., Przepiórka, A., & Jarząbek-Cudo, A. (2020). The role of depression and self-esteem in Facebook intrusion and gaming disorder among young adult gamers. *Psychiatric Quarterly*, *91*(1), 65–76. https://doi.org/10.1007/s11126-019-09685-6

Cudo, A., Wojtasiński, M., Tużnik, P., Griffiths, M. D., & Zabielska-Mendyk, E. (2020). Problematic Facebook use and problematic video gaming as mediators of relationship between impulsivity and life satisfaction among female and male gamers. *PLOS ONE*, *15*(8), e0237610. https://doi.org/10.1371/journal.pone.0237610

Cury, G. S. A., Takamune, D. M., Herrerias, G. S. P., Rivera-Sequeiros, A., de Barros, J. R., Baima, J. P., … Sassaki, L. Y. (2022). Clinical and psychological factors associated with addiction and compensatory use of Facebook among patients with inflammatory bowel disease: A cross-sectional study. *International Journal of General Medicine*, *15*, 1447–1457. https://doi.org/10.2147/IJGM.S334099

da Veiga, G. F., Sotero, L., Pontes, H. M., Cunha, D., Portugal, A., & Relvas, A. P. (2019). Emerging adults and Facebook use: The validation of the Bergen Facebook Addiction Scale (BFAS). *International Journal of Mental Health and Addiction*, *17*(2), 279–294. https://doi.org/10.1007/s11469-018-0018-2

Damota, M. D. (2019). The relationship between Facebook addiction and depression among Madda Walabu University summer students- Cross sectional survey. *New Media and Mass Communication*, *78*. https://doi.org/10.7176/NMMC/78-01

Datu, J. A. D., Valdez, J. P., & Datu, N. (2012). Does facebooking make us sad? Hunting relationship between Facebook use and depression among Filipino adolescents. *International Journal of Research Studies in Educational Technology*, *1*(2), 83–91. https://doi.org/10.5861/ijrset.2012.202

Davidson, T., & Farquhar, L. K. (2014). Correlates of social anxiety, religion, and Facebook. *Journal of Media and Religion*, *13*(4), 208–225. https://doi.org/10.1080/15348423.2014.971566

Dempsey, A. E., O’Brien, K. D., Tiamiyu, M. F., & Elhai, J. D. (2019). Fear of missing out (FoMO) and rumination mediate relations between social anxiety and problematic Facebook use. *Addictive Behaviors Reports*, *9*, 100150. https://doi.org/10.1016/j.abrep.2018.100150

Dibb, B., & Foster, M. (2021). Loneliness and Facebook use: the role of social comparison and rumination. *Heliyon*, *7*(1), e05999. https://doi.org/10.1016/j.heliyon.2021.e05999

Errasti, J., Amigo, I., & Villadangos, M. (2017). Emotional uses of Facebook and Twitter: Its relation with empathy, narcissism, and self-esteem in adolescence. *Psychological Reports*, *120*(6), 997–1018. https://doi.org/10.1177/0033294117713496

Eşkisu, M., Çam, Z., Gelibolu, S., & Rasmussen, K. R. (2020). Trait mindfulness as a protective factor in connections between psychological issues and Facebook addiction among Turkish university students. *Studia Psychologica*, *62*(3), 213–231. https://doi.org/10.31577/sp.2020.03.801

Eşkisu, M., Hoşoğlu, R., & Rasmussen, K. R. (2017). An investigation of the relationship between Facebook usage, Big Five, self-esteem and narcissism. *Computers in Human Behavior*, *69*, 294–301. https://doi.org/10.1016/j.chb.2016.12.036

Farahani, H. A., Kazemi, Z., Aghamohamadi, S., Bakhtiarvand, F., & Ansari, M. (2011). Examining mental health indices in students using Facebook in Iran. *Procedia - Social and Behavioral Sciences*, *28*, 811–814. https://doi.org/10.1016/j.sbspro.2011.11.148

Faranda, M., & Roberts, L. D. (2019). Social comparisons on Facebook and offline: The relationship to depressive symptoms. *Personality and Individual Differences*, *141*, 13–17. https://doi.org/10.1016/j.paid.2018.12.012

Faraon, M., & Kaipainen, M. (2014). Much more to it: The relation between Facebook usage and self-esteem. *Proceedings of the 15th IEEE International Conference on Information Reuse and Integration*, 87–92. https://doi.org/10.1109/IRI.2014.7051876

Flynn, S., Noone, C., & Sarma, K. M. (2018). An exploration of the link between adult attachment and problematic Facebook use. *BMC Psychology*, *6*(1), 34. https://doi.org/10.1186/s40359-018-0245-0

Foroughi, B., Iranmanesh, M., Nikbin, D., & Hyun, S. S. (2019). Are depression and social anxiety the missing link between Facebook addiction and life satisfaction? The interactive effect of needs and self-regulation. *Telematics and Informatics*, *43*, 101247. https://doi.org/10.1016/j.tele.2019.101247

Francis, J. (2022). Elder orphans on Facebook: Implications for mattering and social isolation. *Computers in Human Behavior*, *127*, 107023. https://doi.org/10.1016/j.chb.2021.107023

Frison, E., Bastin, M., Bijttebier, P., & Eggermont, S. (2019). Helpful or harmful? The different relationships between private Facebook interactions and adolescents’ depressive symptoms. *Media Psychology*, *22*(2), 244–272. https://doi.org/10.1080/15213269.2018.1429933

Frison, E., & Eggermont, S. (2015). The impact of daily stress on adolescents’ depressed mood: The role of social support seeking through Facebook. *Computers in Human Behavior*, *44*, 315–325. https://doi.org/10.1016/j.chb.2014.11.070

Frison, E., & Eggermont, S. (2016a). Exploring the relationships between different types of Facebook use, perceived online social support, and adolescents’ depressed mood. *Social Science Computer Review*, *34*(2), 153–171. https://doi.org/10.1177/0894439314567449

Frison, E., & Eggermont, S. (2016b). “Harder, Better, Faster, Stronger”: Negative comparison on Facebook and adolescents’ life satisfaction are reciprocally related. *Cyberpsychology, Behavior, and Social Networking*, *19*(3), 158–164. https://doi.org/10.1089/cyber.2015.0296

Frison, E., & Eggermont, S. (2020). Toward an integrated and differential approach to the relationships between loneliness, different types of Facebook use, and adolescents’ depressed mood. *Communication Research*, *47*(5), 701–728. https://doi.org/10.1177/0093650215617506

Gerson, J., Plagnol, A. C., & Corr, P. J. (2016). Subjective well-being and social media use: Do personality traits moderate the impact of social comparison on Facebook? *Computers in Human Behavior*, *63*, 813–822. https://doi.org/10.1016/j.chb.2016.06.023

Giagkou, S., Hussain, Z., & Pontes, H. M. (2018). Exploring the interplay between passive following on Facebook, fear of missing out, self-esteem, social comparison, age, and life satisfaction in a community-based sample. *International Journal of Psychology & Behavior Analysis*, *4*(2). https://doi.org/10.15344/2455-3867/2018/149

Giota, K. G., & Kleftaras, G. (2013). Facebook social support: A comparative study on depression and personality characteristics. *Proceedings of IADIS International Conference on ICT, Society and Human Beings*, 37–44.

Goljović, N. (2017). Personality traits, self-concept and life satisfaction in the context of Facebook use. In O. Tošković, K. Damnjanović, & L. Lazarević (Eds.), *Proceedings of the 23rd Scientific Conference on Empirical Studies in Psychology* (pp. 96–102).

Gonzales, A. L., & Hancock, J. T. (2011). Mirror, mirror on my Facebook wall: Effects of exposure to Facebook on self-esteem. *Cyberpsychology, Behavior, and Social Networking*, *14*(1–2), 79–83. https://doi.org/10.1089/cyber.2009.0411

González-Nuevo, C., Cuesta, M., & Muñiz, J. (2021). Concern about appearance on Instagram and Facebook: Measurement and links with eating disorders. *Cyberpsychology: Journal of Psychosocial Research on Cyberspace*, *15*(2). https://doi.org/10.5817/CP2021-2-9

Grieve, R., Indian, M., Witteveen, K., Tolan, G. A., & Marrington, J. (2013). Face-to-face or Facebook: Can social connectedness be derived online? *Computers in Human Behavior*, *29*(3), 604–609. https://doi.org/10.1016/j.chb.2012.11.017

große Deters, F., & Mehl, M. R. (2013). Does posting Facebook status updates increase or decrease loneliness? An online social networking experiment. *Social Psychological and Personality Science*, *4*(5), 579–586. https://doi.org/10.1177/1948550612469233

Hanna, E., Ward, L. M., Seabrook, R. C., Jerald, M., Reed, L., Giaccardi, S., & Lippman, J. R. (2017). Contributions of social comparison and self-objectification in mediating associations between Facebook use and emergent adults’ psychological well-being. *Cyberpsychology, Behavior, and Social Networking*, *20*(3), 172–179. https://doi.org/10.1089/cyber.2016.0247

Hanprathet, N., Manwong, M., Khumsri, J., Yingyeun, R., & Phanasathit, M. (2015). Facebook addiction and its relationship with mental health among Thai high school students. *Journal of the Medical Association of Thailand*, *98*(3), 81–90. Retrieved from http://www.ncbi.nlm.nih.gov/pubmed/26387393

He, Q., Turel, O., & Bechara, A. (2018). Association of excessive social media use with abnormal white matter integrity of the corpus callosum. *Psychiatry Research: Neuroimaging*, *278*, 42–47. https://doi.org/10.1016/j.pscychresns.2018.06.008

He, Q., Turel, O., Brevers, D., & Bechara, A. (2017). Excess social media use in normal populations is associated with amygdala-striatal but not with prefrontal morphology. *Psychiatry Research: Neuroimaging*, *269*, 31–35. https://doi.org/10.1016/j.pscychresns.2017.09.003

Ho, T. T. Q. (2021a). Facebook addiction and depression: Loneliness as a moderator and poor sleep quality as a mediator. *Telematics and Informatics*, *61*, 101617. https://doi.org/10.1016/j.tele.2021.101617

Ho, T. T. Q. (2021b). Facebook addiction partially mediated the association between stress symptoms and sleep disturbance among Facebook users. *International Journal of Mental Health and Addiction*. https://doi.org/10.1007/s11469-021-00619-7

Ho, T. T. Q., Huynh, S. Van, & Tran-Chi, V.-L. (2021). Impact of problematic Facebook use, loneliness, and poor sleep quality on mental health. *International Journal of Advanced and Applied Sciences*, *8*(9), 112–118. https://doi.org/10.21833/ijaas.2021.09.015

Ho, T. T. Q., Nguyen, B. P., Nguyen, T. N. B., Pham, T. T. H., & Mai, T. T. T. (2021). Facebook addiction disorder and sleep quality: Loneliness as a mediator. *Psychology and Education Journal*, *58*(5), 4917–4927.

Hong, F.-Y., Huang, D.-H., Lin, H.-Y., & Chiu, S.-L. (2014). Analysis of the psychological traits, Facebook usage, and Facebook addiction model of Taiwanese university students. *Telematics and Informatics*, *31*(4), 597–606. https://doi.org/10.1016/j.tele.2014.01.001

Hosen, M. J., Eva, S. A., Rahman, M. M., Ibrahim, M., Lira, U. F., Hossain, A. B., … Uddin, M. J. (2021). Health impacts of excessive use of Facebook among university students in Bangladesh. *Heliyon*, *7*(6), e07271. https://doi.org/10.1016/j.heliyon.2021.e07271

Hu, X., Kim, A., Siwek, N., & Wilder, D. (2017). The Facebook paradox: Effects of facebooking on individuals’ social relationships and psychological well-being. *Frontiers in Psychology*, *8*, 87. https://doi.org/10.3389/fpsyg.2017.00087

Hummel, A. C., & Smith, A. R. (2015). Ask and you shall receive: Desire and receipt of feedback via Facebook predicts disordered eating concerns. *International Journal of Eating Disorders*, *48*(4), 436–442. https://doi.org/10.1002/eat.22336

Hussain, Z., Simonovic, B., Stupple, E., & Austin, M. (2019). Using eye tracking to explore Facebook use and associations with Facebook addiction, mental well-being, and personality. *Behavioral Sciences*, *9*(2), 19. https://doi.org/10.3390/bs9020019

Iovu, M.-B., Runcan, R., Runcan, P.-L., & Andrioni, F. (2020). Association between Facebook use, depression and family satisfaction: A cross-sectional study of romanian youth. *Iranian Journal of Public Health*, *49*(11), 2111–2119. https://doi.org/10.18502/ijph.v49i11.4728

Jang, K., Park, N., & Song, H. (2016). Social comparison on Facebook: Its antecedents and psychological outcomes. *Computers in Human Behavior*, *62*, 147–154. https://doi.org/10.1016/j.chb.2016.03.082

Jenkins-Guarnieri, M. A., Wright, S. L., & Hudiburgh, L. M. (2012). The relationships among attachment style, personality traits, interpersonal competency, and Facebook use. *Journal of Applied Developmental Psychology*, *33*(6), 294–301. https://doi.org/10.1016/j.appdev.2012.08.001

Jeri-Yabar, A., Sanchez-Carbonel, A., Tito, K., Ramirez-del Castillo, J., Torres-Alcantara, A., Denegri, D., & Carreazo, Y. (2019). Association between social media use (Twitter, Instagram, Facebook) and depressive symptoms: Are Twitter users at higher risk? *International Journal of Social Psychiatry*, *65*(1), 14–19. https://doi.org/10.1177/0020764018814270

Jha, R. K., Shah, D. K., Basnet, S., Paudel, K. R., Sah, P., Sah, A. K., & Adhikari, K. (2016). Facebook use and its effects on the life of health science students in a private medical college of Nepal. *BMC Research Notes*, *9*(1), 378. https://doi.org/10.1186/s13104-016-2186-0

Jin, B. (2013). How lonely people use and perceive Facebook. *Computers in Human Behavior*, *29*(6), 2463–2470. https://doi.org/10.1016/j.chb.2013.05.034

Kalpidou, M., Costin, D., & Morris, J. (2011). The relationship between Facebook and the well-being of undergraduate college students. *Cyberpsychology, Behavior, and Social Networking*, *14*(4), 183–189. https://doi.org/10.1089/cyber.2010.0061

Kanat-Maymon, Y., Almog, L., Cohen, R., & Amichai-Hamburger, Y. (2018). Contingent self-worth and Facebook addiction. *Computers in Human Behavior*, *88*, 227–235. https://doi.org/10.1016/j.chb.2018.07.011

Kang, S., Chung, W., Mora, A. R., & Chung, Y. (2013). Facebook comparisons among adolescents: How do identification and contrast relate to wellbeing. *Asian Journal of Information and Communications*, *5*(2), 1–21.

Karakose, T., Yirci, R., Uygun, H., & Ozdemir, T. Y. (2016). Relationship between high school students’ Facebook addiction and loneliness status. *Eurasia Journal of Mathematics, Science and Technology Education*, *12*(9), 2419–2429. https://doi.org/10.12973/eurasia.2016.1557a

Khalil, S. A., Kamal, H., & Elkholy, H. (2022). The prevalence of problematic internet use among a sample of Egyptian adolescents and its psychiatric comorbidities. *International Journal of Social Psychiatry*, *68*(2), 294–300. https://doi.org/10.1177/0020764020983841

Khattak, A. F., Ahmad, S., & Mohammad, H. (2017). Facebook addiction and depression: A comparative study of gender differences. *PUTAJ - Humanities and Social Sciences*, *25*(1–2), 55–62.

Kim, E. S., James, P., Zevon, E. S., Trudel-Fitzgerald, C., Kubzansky, L. D., & Grodstein, F. (2020). Social media as an emerging data resource for epidemiologic research: Characteristics of regular and nonregular social media users in nurses’ health study II. *American Journal of Epidemiology*, *189*(2), 156–161. https://doi.org/10.1093/aje/kwz224

Koc, M., & Gulyagci, S. (2013). Facebook addiction among Turkish college students: The role of psychological health, demographic, and usage characteristics. *Cyberpsychology, Behavior, and Social Networking*, *16*(4), 279–284. https://doi.org/10.1089/cyber.2012.0249

Kross, E., Verduyn, P., Demiralp, E., Park, J., Lee, D. S., Lin, N., … Ybarra, O. (2013). Facebook use predicts declines in subjective well-being in young adults. *PLOS ONE*, *8*(8), e69841. https://doi.org/10.1371/journal.pone.0069841

Kulkarni, R., & Deshpande, A. (2019). Relationship between Facebook addiction, depression and shyness among college students in Mumbai. *Indian Journal of Mental Health*, *6*(2), 157–164. https://doi.org/10.30877/IJMH.6.2.2019.157-164

Kumar, B., Banik, P., & Islam, M. A. (2019). Social network, Facebook use and loneliness: A comparative analysis between public and private university students in Bangladesh. *International Journal of Psychological and Brain Sciences*, *4*(2), 20. https://doi.org/10.11648/j.ijpbs.20190402.13

Labrague, L. J. (2014). Facebook use and adolescents’ emotional states of depression, anxiety, and stress. *Health Science Journal*, *8*(1), 80–89.

Lee-Won, R. J., Herzog, L., & Park, S. G. (2015). Hooked on Facebook: The role of social anxiety and need for social assurance in problematic use of Facebook. *Cyberpsychology, Behavior, and Social Networking*, *18*(10), 567–574. https://doi.org/10.1089/cyber.2015.0002

Lee, J.-E. R., Moore, D. C., Park, E.-A., & Park, S. G. (2012). Who wants to be “friend-rich”? Social compensatory friending on Facebook and the moderating role of public self-consciousness. *Computers in Human Behavior*, *28*(3), 1036–1043. https://doi.org/10.1016/j.chb.2012.01.006

Lee, S. (2020). A study on the effect of comparison with others and social support on life satisfaction of Facebook. *Advances in Journalism and Communication*, *8*(1), 1–15. https://doi.org/10.4236/ajc.2020.81001

Lee, S. Y. (2014). How do people compare themselves with others on social network sites?: The case of Facebook. *Computers in Human Behavior*, *32*, 253–260. https://doi.org/10.1016/j.chb.2013.12.009

Lemieux, R., Lajoie, S., & Trainor, N. E. (2013). Affinity-seeking, social loneliness, and social avoidance among Facebook users. *Psychological Reports*, *112*(2), 545–552. https://doi.org/10.2466/07.PR0.112.2.545-552

Lim, M., & Yang, Y. (2019). Upward social comparison and Facebook users’ grandiosity: Examining the effect of envy on loneliness and subjective well-being. *Online Information Review*, *43*(4), 635–652. https://doi.org/10.1108/OIR-04-2017-0137

Locatelli, S. M., Kluwe, K., & Bryant, F. B. (2012). Facebook use and the tendency to ruminate among college students: Testing mediational hypotheses. *Journal of Educational Computing Research*, *46*(4), 377–394. https://doi.org/10.2190/EC.46.4.d

Longua Peterson, J., Giguere, B., & Sherman, J. (2017). Social connection and social networking: Daily conflict increases nightly Facebook use among avoidant participants. *Self and Identity*, *16*(2), 215–230. https://doi.org/10.1080/15298868.2016.1247011

Lönnqvist, J.-E., & große Deters, F. (2016). Facebook friends, subjective well-being, social support, and personality. *Computers in Human Behavior*, *55*, 113–120. https://doi.org/10.1016/j.chb.2015.09.002

Lou, L. L., Yan, Z., Nickerson, A., & McMorris, R. (2012). An examination of the reciprocal relationship of loneliness and Facebook use among first-year college students. *Journal of Educational Computing Research*, *46*(1), 105–117. https://doi.org/10.2190/EC.46.1.e

Louragli, I., Ahami, A., Khadmaoui, A., Aboussaleh, Y., & Chaker Lamrani, A. (2019). Behavioral analysis of adolescent’s students addicted to Facebook and its impact on performance and mental health. *Acta Neuropsychologica*, *17*(4), 427–439. https://doi.org/10.5604/01.3001.0013.6550

Luqman, A., Cao, X., Ali, A., Masood, A., & Yu, L. (2017). Empirical investigation of Facebook discontinues usage intentions based on SOR paradigm. *Computers in Human Behavior*, *70*, 544–555. https://doi.org/10.1016/j.chb.2017.01.020

Mabe, A. G., Forney, K. J., & Keel, P. K. (2014). Do you “like” my photo? Facebook use maintains eating disorder risk. *International Journal of Eating Disorders*, *47*(5), 516–523. https://doi.org/10.1002/eat.22254

Maglunog, G. P., & Dy, M. F. R. (2019). Facebook usage and depressıon levels of selected Filipino college students. *International Journal of Psychology and Educational Studies*, *6*(2), 35–50. https://doi.org/10.17220/ijpes.2019.02.004

Malik, S., & Khan, M. (2015). Impact of Facebook addiction on narcissistic behavior and self-esteem among student. *The Journal of the Pakistan Medical Association*, *65*(3), 260–263. Retrieved from http://www.ncbi.nlm.nih.gov/pubmed/25933557

Manago, A. M., Taylor, T., & Greenfield, P. M. (2012). Me and my 400 friends: The anatomy of college students’ Facebook networks, their communication patterns, and well-being. *Developmental Psychology*, *48*(2), 369–380. https://doi.org/10.1037/a0026338

Mannino, G., Salerno, L., Bonfanti, R. C., Albano, G., & Lo Coco, G. (2021). The impact of Facebook use on self-reported eating disorders during the COVID-19 lockdown. *BMC Psychiatry*, *21*(1), 611. https://doi.org/10.1186/s12888-021-03628-x

Marder, B., Joinson, A., Shankar, A., & Thirlaway, K. (2016). Strength matters: Self-presentation to the strongest audience rather than lowest common denominator when faced with multiple audiences in social network sites. *Computers in Human Behavior*, *61*, 56–62. https://doi.org/10.1016/j.chb.2016.03.005

Marengo, D., Montag, C., Sindermann, C., Elhai, J. D., & Settanni, M. (2021). Examining the links between active Facebook use, received likes, self-esteem and happiness: A study using objective social media data. *Telematics and Informatics*, *58*, 101523. https://doi.org/10.1016/j.tele.2020.101523

Masciantonio, A., Bourguignon, D., Bouchat, P., Balty, M., & Rimé, B. (2021). Don’t put all social network sites in one basket: Facebook, Instagram, Twitter, TikTok, and their relations with well-being during the COVID-19 pandemic. *PLOS ONE*, *16*(3), e0248384. https://doi.org/10.1371/journal.pone.0248384

Mauri, M., Cipresso, P., Balgera, A., Villamira, M., & Riva, G. (2011). Why is Facebook so successful? Psychophysiological measures describe a core flow state while using Facebook. *Cyberpsychology, Behavior, and Social Networking*, *14*(12), 723–731. https://doi.org/10.1089/cyber.2010.0377

McCloskey, W., Iwanicki, S., Lauterbach, D., Giammittorio, D. M., & Maxwell, K. (2015). Are Facebook “friends” helpful? Development of a Facebook-based measure of social support and examination of relationships among depression, quality of life, and social support. *Cyberpsychology, Behavior, and Social Networking*, *18*(9), 499–505. https://doi.org/10.1089/cyber.2014.0538

McCord, B., Rodebaugh, T. L., & Levinson, C. A. (2014). Facebook: Social uses and anxiety. *Computers in Human Behavior*, *34*, 23–27. https://doi.org/10.1016/j.chb.2014.01.020

Metzler, A., & Scheithauer, H. (2017). The long-term benefits of positive self-presentation via profile pictures, number of friends and the initiation of relationships on Facebook for adolescents’ self-esteem and the initiation of offline relationships. *Frontiers in Psychology*, *8*. https://doi.org/10.3389/fpsyg.2017.01981

Michikyan, M., Dennis, J., & Subrahmanyam, K. (2015). Can you guess who I am? Real, ideal, and false self-presentation on Facebook among emerging adults. *Emerging Adulthood*, *3*(1), 55–64. https://doi.org/10.1177/2167696814532442

Montag, C., Markowetz, A., Blaszkiewicz, K., Andone, I., Lachmann, B., Sariyska, R., … Markett, S. (2017). Facebook usage on smartphones and gray matter volume of the nucleus accumbens. *Behavioural Brain Research*, *329*, 221–228. https://doi.org/10.1016/j.bbr.2017.04.035

Moreno, M. A., Stewart, M., Pumper, M., Cox, E., Young, H., Zhang, C., & Eickhoff, J. (2014). Facebook use during a stressful event: A pilot evaluation investigating Facebook use patterns and biologic stress response. *Bulletin of Science, Technology & Society*, *34*(3–4), 94–98. https://doi.org/10.1177/0270467614561674

Morin-Major, J. K., Marin, M.-F., Durand, N., Wan, N., Juster, R.-P., & Lupien, S. J. (2016). Facebook behaviors associated with diurnal cortisol in adolescents: Is befriending stressful? *Psychoneuroendocrinology*, *63*, 238–246. https://doi.org/10.1016/j.psyneuen.2015.10.005

Nabi, R. L., Prestin, A., & So, J. (2013). Facebook friends with (health) benefits? Exploring social network site use and perceptions of social support, stress, and well-being. *Cyberpsychology, Behavior, and Social Networking*, *16*(10), 721–727. https://doi.org/10.1089/cyber.2012.0521

Nasser, N. S., Ling, L. J., Rashid, A. A., Sharifat, H., Hamid, S. A., Rahim, E. A., … Suppiah, S. (2019). Assessment of problematic Facebook use among undergraduate students in UPM correlated with depression, anxiety and stress. *International Journal of Public Health and Clinical Sciences*, *6*(4), 113–132. https://doi.org/10.32827/ijphcs.6.4.113

Nazzal, Z., Rabee, H., Ba’ar, M., & Berte, D. (2021). Virtually alone: Excessive Facebook use and mental health risk in Palestine, a cross sectional study. *Palestinian Medical and Pharmaceutical Journal*, *6*(1), 53–62.

Nisar, T. M., Prabhakar, G., Ilavarasan, P. V., & Baabdullah, A. M. (2019). Facebook usage and mental health: An empirical study of role of non-directional social comparisons in the UK. *International Journal of Information Management*, *48*, 53–62. https://doi.org/10.1016/j.ijinfomgt.2019.01.017

Nizami, G. N., Naeem, Z., Arzoo, K., & Ismail, S. (2017). Impact of Facebook addiction on academic performance among undergraduate students. *Pakistan Journal of Rehabilitation*, *6*(1), 45–50.

Norman, P., Elavarasan, K., & Dhandapani, T. (2017). Facebook addiction and depression in adults [19 years-64 years]. *International Journal Of Community Medicine And Public Health*, *4*(8), 2999–3004. https://doi.org/10.18203/2394-6040.ijcmph20173361

O’Sullivan, A., & Hussain, Z. (2017). An exploratory study of Facebook intensity and its links To narcissism, stress, and self-esteem. *Journal of Addictive Behaviors, Therapy & Rehabilitation*, *6*(1). https://doi.org/10.4172/2324-9005.1000161

Ögel-Balaban, H., & Altan, Ş. (2020). The use of Facebook by Turkish mothers: Its reasons and outcomes. *Journal of Child and Family Studies*, *29*(3), 780–790. https://doi.org/10.1007/s10826-019-01568-5

Omar, B., & Subramanian, K. (2013). Addicted to Facebook: Examining the roles of personality characteristics, gratifications sought and Facebook exposure among youths. *GSTF Journal on Media & Communications*, *1*(1), 54–65. https://doi.org/10.5176/2335-6618_1.1.6

Omolayo, B. O., Balogun, S. K., & Omole, O. C. (2013). Influence of exposure to Facebook on self-esteem. *European Scientific Journal*, *9*(11), 148–159.

Ozimek, P., & Bierhoff, H.-W. (2020). All my online-friends are better than me – three studies about ability-based comparative social media use, self-esteem, and depressive tendencies. *Behaviour & Information Technology*, *39*(10), 1110–1123. https://doi.org/10.1080/0144929X.2019.1642385

Ozimek, P., Bierhoff, H.-W., & Rohmann, E. (2021). How downward and upward comparisons on Facebook influence grandiose and vulnerable narcissists’ self-esteem—A priming study. *Behavioral Sciences*, *11*(3), 39. https://doi.org/10.3390/bs11030039

Pal, A., Shankar, S. H., & Masthi, N. R. R. (2018). Correlation of personality and mental well-being with Facebook use: Does gender play a role? *RGUHS National Journal of Public Health*, *3*(1), 26–34.

Park, S., Lee, S. W., Kwak, J., Cha, M., & Jeong, B. (2013). Activities on Facebook reveal the depressive state of users. *Journal of Medical Internet Research*, *15*(10), e217. https://doi.org/10.2196/jmir.2718

Park, S. Y., & Baek, Y. M. (2018). Two faces of social comparison on Facebook: The interplay between social comparison orientation, emotions, and psychological well-being. *Computers in Human Behavior*, *79*, 83–93. https://doi.org/10.1016/j.chb.2017.10.028

Phu, B., & Gow, A. J. (2019). Facebook use and its association with subjective happiness and loneliness. *Computers in Human Behavior*, *92*, 151–159. https://doi.org/10.1016/j.chb.2018.11.020

Primi, C., Fioravanti, G., Casale, S., & Donati, M. A. (2021). Measuring problematic Facebook use among adolescents and young adults with the Bergen Facebook Addiction Scale: A psychometric analysis by applying item response theory. *International Journal of Environmental Research and Public Health*, *18*(6), 2979. https://doi.org/10.3390/ijerph18062979

Przepiórka, A., & Błachnio, A. (2020). The role of Facebook intrusion, depression, and future time perspective in sleep problems among adolescents. *Journal of Research on Adolescence*, *30*(2), 559–569. https://doi.org/10.1111/jora.12543

Przepiórka, A., Błachnio, A., Sullman, M., Gorbaniuk, O., Siu, N. Y.-F., Hill, T., … Font-Mayolas, S. (2021). Facebook intrusion as a mediator between positive capital and general distress: A cross-cultural study. *Frontiers in Psychiatry*, *12*. https://doi.org/10.3389/fpsyt.2021.667536

Puccio, F., Kalathas, F., Fuller-Tyszkiewicz, M., & Krug, I. (2016). A revised examination of the dual pathway model for bulimic symptoms: The importance of social comparisons made on Facebook and sociotropy. *Computers in Human Behavior*, *65*, 142–150. https://doi.org/10.1016/j.chb.2016.08.018

Rachubińska, K., Cybulska, A. M., & Grochans, E. (2021). The relationship between loneliness, depression, internet and social media addiction among young Polish women. *European Review for Medical and Pharmacological Sciences*, *25*(4), 1982–1989. https://doi.org/10.26355/eurrev_202102_25099

Rae, J. R., & Lonborg, S. D. (2015). Do motivations for using Facebook moderate the association between Facebook use and psychological well-being? *Frontiers in Psychology*, *6*, 771. https://doi.org/10.3389/fpsyg.2015.00771

Rahman, M. M., & Zakaria, M. (2021). Facebook use and its relationship with physical and mental health among university students in Bangladesh. *Communication and Media in Asia Pacific*, *4*(2), 33–44. https://doi.org/10.14456/cmap.2021.9

Rajesh, T., & Rangaiah, B. (2020). Facebook addiction and personality. *Heliyon*, *6*(1), e03184. https://doi.org/10.1016/j.heliyon.2020.e03184

Rauch, S. M., Strobel, C., Bella, M., Odachowski, Z., & Bloom, C. (2014). Face to face versus Facebook: Does exposure to social networking web sites augment or attenuate physiological arousal among the socially anxious? *Cyberpsychology, Behavior, and Social Networking*, *17*(3), 187–190. https://doi.org/10.1089/cyber.2012.0498

Rosen, L. D., Whaling, K., Rab, S., Carrier, L. M., & Cheever, N. A. (2013). Is Facebook creating “iDisorders”? The link between clinical symptoms of psychiatric disorders and technology use, attitudes and anxiety. *Computers in Human Behavior*, *29*(3), 1243–1254. https://doi.org/10.1016/j.chb.2012.11.012

Rosenthal, S. R., Buka, S. L., Marshall, B. D. L., Carey, K. B., & Clark, M. A. (2016). Negative experiences on Facebook and depressive symptoms among young adults. *Journal of Adolescent Health*, *59*(5), 510–516. https://doi.org/10.1016/j.jadohealth.2016.06.023

Rus, H. M., & Tiemensma, J. (2017). Social media under the skin: Facebook use after acute stress impairs cortisol recovery. *Frontiers in Psychology*, *8*. https://doi.org/10.3389/fpsyg.2017.01609

Rus, H. M., & Tiemensma, J. (2018). Social media as a shield: Facebook buffers acute stress. *Physiology & Behavior*, *185*, 46–54. https://doi.org/10.1016/j.physbeh.2017.12.021

Ryan, T., & Xenos, S. (2011). Who uses Facebook? An investigation into the relationship between the Big Five, shyness, narcissism, loneliness, and Facebook usage. *Computers in Human Behavior*, *27*(5), 1658–1664. https://doi.org/10.1016/j.chb.2011.02.004

Saleem, M., Irshad, R., Zafar, M., & Tahi, M. A. (2016). Facebook addiction causing loneliness among higher learning students of Pakistan: A linear relationship. *Journal of Applied and Emerging Sciences*, *5*(1), 26–31.

Satici, S. A. (2019). Facebook addiction and subjective well-being: A study of the mediating role of shyness and loneliness. *International Journal of Mental Health and Addiction*, *17*(1), 41–55. https://doi.org/10.1007/s11469-017-9862-8

Satici, S. A., & Uysal, R. (2015). Well-being and problematic Facebook use. *Computers in Human Behavior*, *49*, 185–190. https://doi.org/10.1016/j.chb.2015.03.005

Scherr, S., & Brunet, A. (2017). Differential Influences of Depression and Personality Traits on the Use of Facebook. *Social Media + Society*, *3*(1), 1–14. https://doi.org/10.1177/2056305117698495

Scherr, S., Toma, C. L., & Schuster, B. (2019). Depression as a predictor of Facebook surveillance and envy: Longitudinal evidence from a cross-lagged panel study in Germany. *Journal of Media Psychology*, *31*(4), 196–202. https://doi.org/10.1027/1864-1105/a000247

Schmuck, D., Karsay, K., Matthes, J., & Stevic, A. (2019). “Looking Up and Feeling Down”. The influence of mobile social networking site use on upward social comparison, self-esteem, and well-being of adult smartphone users. *Telematics and Informatics*, *42*, 101240. https://doi.org/10.1016/j.tele.2019.101240

Sehar, H., Razaq, N., Kanwal, S., Jogezai, A. K., Ashraf, M. T., & Tehzeeb, M. (2022). Relationship of personality traits and self-esteem with Facebook addiction among university students. *Rawal Medical Journal*, *47*(1), 227–230.

Seran, J. A., Lerik, M. D. C., Wijaya, R. P. C., & Adu, A. A. (2020). The relationship between self esteem and Facebook addiction in adolescents in Kupang City. *Journal of Health and Behavioral Science*, *2*(4), 252–263.

Shakya, H. B., & Christakis, N. A. (2017). Association of Facebook use with compromised well-being: A longitudinal study. *American Journal of Epidemiology*, *185*(3), 203–211. https://doi.org/10.1093/aje/kww189

Shaw, A. M., Timpano, K. R., Tran, T. B., & Joormann, J. (2015). Correlates of Facebook usage patterns: The relationship between passive Facebook use, social anxiety symptoms, and brooding. *Computers in Human Behavior*, *48*, 575–580. https://doi.org/10.1016/j.chb.2015.02.003

Shettar, M., Karkal, R., Kakunje, A., Mendonsa, R. D., & Chandran, V. M. (2017). Facebook addiction and loneliness in the post-graduate students of a university in southern India. *International Journal of Social Psychiatry*, *63*(4), 325–329. https://doi.org/10.1177/0020764017705895

Sillence, E., Saxton, T. K., & Pollet, T. V. (2021). Facebook social use and anxiety: A replication attempt. *Human Communication & Technology*, *2*(1), 19–36.

Simoncic, T. E., Kuhlman, K. R., Vargas, I., Houchins, S., & Lopez-Duran, N. L. (2014). Facebook use and depressive symptomatology: Investigating the role of neuroticism and extraversion in youth. *Computers in Human Behavior*, *40*, 1–5. https://doi.org/10.1016/j.chb.2014.07.039

Skues, J. L., Williams, B., & Wise, L. (2012). The effects of personality traits, self-esteem, loneliness, and narcissism on Facebook use among university students. *Computers in Human Behavior*, *28*(6), 2414–2419. https://doi.org/10.1016/j.chb.2012.07.012

Smith, A. R., Hames, J. L., & Joiner Jr., T. E. (2013). Status update: Maladaptive Facebook usage predicts increases in body dissatisfaction and bulimic symptoms. *Journal of Affective Disorders*, *149*(1–3), 235–240. https://doi.org/10.1016/j.jad.2013.01.032

Smith, T., & Short, A. (2022). Needs affordance as a key factor in likelihood of problematic social media use: Validation, latent Profile analysis and comparison of TikTok and Facebook problematic use measures. *Addictive Behaviors*, *129*, 107259. https://doi.org/10.1016/j.addbeh.2022.107259

Soraci, P., Ferrari, A., Barberis, N., Luvarà, G., Urso, A., Del Fante, E., & Griffiths, M. D. (2020). Psychometric analysis and validation of the Italian Bergen Facebook Addiction Scale. *International Journal of Mental Health and Addiction*. https://doi.org/10.1007/s11469-020-00346-5

Sotero, L., da Veiga, G. F., Carreira, D., Portugal, A., & Relvas, A. P. (2019). Facebook addiction and emerging adults: The influence of sociodemographic variables, family communication, and differentiation of self. *Escritos de Psicología*, *12*(2), 81–92. https://doi.org/10.24310/espsiescpsi.v12i2.9986

Srivastava, A. (2015). The effect of Facebook use on life satisfaction and subjective happiness of college students. *International Journal of Indian Psychology*, *2*(4). https://doi.org/10.25215/0204.078

Stănculescu, E., & Griffiths, M. D. (2021). Anxious attachment and Facebook addiction: The mediating role of need to belong, self-esteem, and Facebook use to meet romantic partners. *International Journal of Mental Health and Addiction*. https://doi.org/10.1007/s11469-021-00598-9

Steers, M.-L. N., Wickham, R. E., & Acitelli, L. K. (2014). Seeing everyone else’s highlight reels: How Facebook usage is linked to depressive symptoms. *Journal of Social and Clinical Psychology*, *33*(8), 701–731. https://doi.org/10.1521/jscp.2014.33.8.701

Sternberg, N., Luria, R., Chandhok, S., Vickers, B., Kross, E., & Sheppes, G. (2020). When Facebook and finals collide - procrastinatory social media usage predicts enhanced anxiety. *Computers in Human Behavior*, *109*, 106358. https://doi.org/10.1016/j.chb.2020.106358

Sternberg, N., Luria, R., & Sheppes, G. (2018). For whom is social-network usage associated with anxiety? The moderating role of neural working-memory filtering of Facebook information. *Cognitive, Affective, & Behavioral Neuroscience*, *18*(6), 1145–1158. https://doi.org/10.3758/s13415-018-0627-z

Stieger, S. (2019). Facebook usage and life satisfaction. *Frontiers in Psychology*, *10*. https://doi.org/10.3389/fpsyg.2019.02711

Tandoc Jr., E. C., Ferrucci, P., & Duffy, M. (2015). Facebook use, envy, and depression among college students: Is facebooking depressing? *Computers in Human Behavior*, *43*, 139–146. https://doi.org/10.1016/j.chb.2014.10.053

Tandoc Jr., E. C., & Goh, Z. H. (2021). Is facebooking really depressing? Revisiting the relationships among social media use, envy, and depression. *Information, Communication & Society*, 1–17. https://doi.org/10.1080/1369118X.2021.1954975

Tazghini, S., & Siedlecki, K. L. (2013). A mixed method approach to examining Facebook use and its relationship to self-esteem. *Computers in Human Behavior*, *29*(3), 827–832. https://doi.org/10.1016/j.chb.2012.11.010

Teo, A. R., Chan, B. K., Saha, S., & Nicolaidis, C. (2019). Frequency of social contact in-person vs. on Facebook: An examination of associations with psychiatric symptoms in military veterans. *Journal of Affective Disorders*, *243*, 375–380. https://doi.org/10.1016/j.jad.2018.09.043

Teppers, E., Luyckx, K., Klimstra, T. A., & Goossens, L. (2014). Loneliness and Facebook motives in adolescence: A longitudinal inquiry into directionality of effect. *Journal of Adolescence*, *37*(5), 691–699. https://doi.org/10.1016/j.adolescence.2013.11.003

Tobin, S. J., & Graham, S. (2020). Feedback sensitivity as a mediator of the relationship between attachment anxiety and problematic Facebook use. *Cyberpsychology, Behavior, and Social Networking*, *23*(8), 562–566. https://doi.org/10.1089/cyber.2019.0560

Tosun, L. P., & Kaşdarma, E. (2020). Passive Facebook use and depression: A study of the roles of upward comparisons, emotions, and friendship type. *Journal of Media Psychology*, *32*(4), 165–175. https://doi.org/10.1027/1864-1105/a000269

Triệu, P., Ellison, N. B., Schoenebeck, S. Y., & Brewer, R. N. (2021). Implications of Facebook engagement types and feed’s social content for self-esteem via social comparison processes. *Social Media + Society*, *7*(3). https://doi.org/10.1177/20563051211042400

Tromholt, M. (2016). The Facebook experiment: Quitting Facebook leads to higher levels of well-being. *Cyberpsychology, Behavior, and Social Networking*, *19*(11), 661–666. https://doi.org/10.1089/cyber.2016.0259

Türkmen, O. O., Kavaklı, M., & Ak, M. (2022). The multiple mediating roles of self-esteem and happiness in the relationship between loneliness and depression in Facebook and Instagram users. *Journal of Clinical Psychiatry*, *25*(1), 23–30. https://doi.org/10.5505/kpd.2022.79663

Uram, P., & Skalski, S. (2022). Still logged in? The link between Facebook addiction, FoMO, self-esteem, life satisfaction and loneliness in social media users. *Psychological Reports*, *125*(1), 218–231. https://doi.org/10.1177/0033294120980970

Uttravanich, A., & Blauw, J. N. (2018). Facebook use, appearance comparison, body dissatisfacion, and self-esteem in Thai female Facebook users. *Scholar: Human Sciences*, *10*(1), 201–2013.

Valenzuela, S., Park, N., & Kee, K. F. (2009). Is there social capital in a social network site?: Facebook use and college students’ life satisfaction, trust, and participation. *Journal of Computer-Mediated Communication*, *14*(4), 875–901. https://doi.org/10.1111/j.1083-6101.2009.01474.x

Vanman, E. J., Baker, R., & Tobin, S. J. (2018). The burden of online friends: The effects of giving up Facebook on stress and well-being. *The Journal of Social Psychology*, *158*(4), 496–508. https://doi.org/10.1080/00224545.2018.1453467

Vannucci, A., Ohannessian, C. M., & Gagnon, S. (2019). Use of multiple social media platforms in relation to psychological functioning in emerging adults. *Emerging Adulthood*, *7*(6), 501–506. https://doi.org/10.1177/2167696818782309

Verseillié, E., Laconi, S., Castro-Calvo, J., & Chabrol, H. (2021). Psychometric evaluation of the Bergen Facebook Addiction Scale: One- or two-factor solution? *International Journal of Mental Health and Addiction*. https://doi.org/10.1007/s11469-021-00668-y

Vigil, T. R., & Wu, H. D. (2015). Facebook users’ engagement and perceived life satisfaction. *Media and Communication*, *3*(1), 5–16. https://doi.org/10.17645/mac.v3i1.199

Vogel, E. A., Rose, J. P., Okdie, B. M., Eckles, K., & Franz, B. (2015). Who compares and despairs? The effect of social comparison orientation on social media use and its outcomes. *Personality and Individual Differences*, *86*, 249–256. https://doi.org/10.1016/j.paid.2015.06.026

Vogel, E. A., Rose, J. P., Roberts, L. R., & Eckles, K. (2014). Social comparison, social media, and self-esteem. *Psychology of Popular Media Culture*, *3*(4), 206–222. https://doi.org/10.1037/ppm0000047

Walburg, V., Mialhes, A., & Moncla, D. (2016). Does school-related burnout influence problematic Facebook use? *Children and Youth Services Review*, *61*, 327–331. https://doi.org/10.1016/j.childyouth.2016.01.009

Walker, M., Thornton, L., De Choudhury, M., Teevan, J., Bulik, C. M., Levinson, C. A., & Zerwas, S. (2015). Facebook use and disordered eating in college-aged women. *Journal of Adolescent Health*, *57*(2), 157–163. https://doi.org/10.1016/j.jadohealth.2015.04.026

Wang, K., Frison, E., Eggermont, S., & Vandenbosch, L. (2018). Active public Facebook use and adolescents’ feelings of loneliness: Evidence for a curvilinear relationship. *Journal of Adolescence*, *67*(1), 35–44. https://doi.org/10.1016/j.adolescence.2018.05.008

Wang, S. S. (2013). “I Share, Therefore I Am”: Personality traits, life satisfaction, and Facebook check-ins. *Cyberpsychology, Behavior, and Social Networking*, *16*(12), 870–877. https://doi.org/10.1089/cyber.2012.0395

Wang, T.-J., Kang, C.-L., Tsai, J.-L., Song, W.-T., & Lien, A. S.-Y. (2021). Social media (Facebook) improper use and the influence of sleeping quality in Taiwan’s university students. *Science Progress*, *104*(2). https://doi.org/10.1177/00368504211011878

Wenninger, H., Krasnova, H., & Buxmann, P. (2014). Activity matters: Investigating the influence of Facebook on life satisfaction of teenage users. *Proceedings of the 22nd European Conference on Information Systems*.

Whitman, C. N., & Gottdiener, W. H. (2016). The cyber self: Facebook as a predictor of well‐being. *International Journal of Applied Psychoanalytic Studies*, *13*(2), 142–162. https://doi.org/10.1002/aps.1431

Wolniczak, I., Cáceres-Del Aguila, J. A., Palma-Ardiles, G., Arroyo, K. J., Solís-Visscher, R., Paredes-Yauri, S., … Bernabe-Ortiz, A. (2013). Association between Facebook dependence and poor sleep quality: A study in a sample of undergraduate students in Peru. *PLOS ONE*, *8*(3), e59087. https://doi.org/10.1371/journal.pone.0059087

Wright, E. J., White, K. M., & Obst, P. L. (2018). Facebook false self-presentation behaviors and negative mental health. *Cyberpsychology, Behavior, and Social Networking*, *21*(1), 40–49. https://doi.org/10.1089/cyber.2016.0647

Wright, K. B., Rosenberg, J., Egbert, N., Ploeger, N. A., Bernard, D. R., & King, S. (2013). Communication competence, social support, and depression among college students: A model of Facebook and face-to-face support network influence. *Journal of Health Communication*, *18*(1), 41–57. https://doi.org/10.1080/10810730.2012.688250

Xie, W., & Karan, K. (2019). Predicting Facebook addiction and state anxiety without Facebook by gender, trait anxiety, Facebook intensity, and different Facebook activities. *Journal of Behavioral Addictions*, *8*(1), 79–87. https://doi.org/10.1556/2006.8.2019.09

Ye, S., Ho, K. K. W., & Zerbe, A. (2021). The effects of social media usage on loneliness and well-being: Analysing friendship connections of Facebook, Twitter and Instagram. *Information Discovery and Delivery*, *49*(2), 136–150. https://doi.org/10.1108/IDD-08-2020-0091

Yeshua-Katz, D., & Zilberstein, T. (2021). Facebook use and well-being among army veterans with PTSD. *International Journal of Communication*, *15*, 5029–5050.

Zaffar, M., Mahmood, S., Saleem, M., & Zakaria, E. (2015). Facebook addiction: Relation with depression, anxiety, loneliness and academic performance of Pakistani students. *Science International*, *27*(3), 2469–2475.

Zhang, R. (2017). The stress-buffering effect of self-disclosure on Facebook: An examination of stressful life events, social support, and mental health among college students. *Computers in Human Behavior*, *75*, 527–537. https://doi.org/10.1016/j.chb.2017.05.043
